# A tRNA-derived fragment present in *E*. *coli* OMVs regulates host cell gene expression and proliferation

**DOI:** 10.1371/journal.ppat.1010827

**Published:** 2022-09-15

**Authors:** Idrissa Diallo, Jeffrey Ho, Marine Lambert, Abderrahim Benmoussa, Zeinab Husseini, David Lalaouna, Eric Massé, Patrick Provost

**Affiliations:** 1 CHU de Québec-Université Laval Research Center/CHUL Pavilion, Department of Microbiology, Infectious Diseases and Immunology, Faculty of Medicine, Université Laval, Quebec City, Quebec, Canada; 2 CRCHUS, RNA Group, Department of Biochemistry and Functional Genomics, Faculty of Medicine and Health Sciences, Université de Sherbrooke, Sherbrooke, Quebec, Canada; INSERM, FRANCE

## Abstract

RNA-sequencing has led to a spectacular increase in the repertoire of bacterial sRNAs and improved our understanding of their biological functions. Bacterial sRNAs have also been found in outer membrane vesicles (OMVs), raising questions about their potential involvement in bacteria-host relationship, but few studies have documented this issue. Recent RNA-Sequencing analyses of bacterial RNA unveiled the existence of abundant very small RNAs (vsRNAs) shorter than 16 nt. These especially include tRNA fragments (tRFs) that are selectively loaded in OMVs and are predicted to target host mRNAs. Here, in *Escherichia coli* (*E*. *coli)*, we report the existence of an abundant vsRNA, Ile-tRF-5X, which is selectively modulated by environmental stress, while remaining unaffected by inhibition of transcription or translation. Ile-tRF-5X is released through OMVs and can be transferred to human HCT116 cells, where it promoted MAP3K4 expression. Our findings provide a novel perspective and paradigm on the existing symbiosis between bacteria and human cells.

## Author summary

We previously outlined by RNA-Sequencing (RNA-seq) the existence of abundant very small (<16 nt) bacterial and eukaryote RNA (vsRNA) population with potential regulatory functions. However, it is not exceptional to see vsRNA species removed from the RNA-seq libraries or datasets because being considered as random degradation products. As a proof of concept, we present in this study a 13 nt in length isoleucine tRNA-derived fragment (Ile-tRF-5X) which is selectively modulated by nutritional and thermal stress while remaining unaffected by transcription and translation inhibitions. We also showed that OMVs and their Ile-tRF-5X vsRNAs are delivered into human HCT116 cells and both can promote host cell gene expression and proliferation. Ile-tRF-5X appears to regulate gene silencing properties of miRNAs by competition. Our findings provide a novel perspective and paradigm on the existing symbiosis between hosts and bacteria but also brings a new insight of host-pathogen interactions mediated by tRFs which remain so far poorly characterized in bacteria.

## Introduction

High-throughput sequencing (HTS) techniques have led to an explosion of knowledge on non-coding RNAs (ncRNAs) [1] and, more importantly, they have shed light on the extent to which they are involved in the regulation of gene expression in both prokaryotes and eukaryotes [2–4]. In bacteria, ncRNAs are generally referred to as small non-coding RNAs (sRNAs). The regulatory role of bacterial sRNAs was first demonstrated in extrachromosomal systems [5] and then fortuitously in intrachromosomal systems, in which the sRNA MicF significantly inhibited translation of the *ompF* gene through binding to its 5’ end [6].

Most of the sRNAs rely on base pairing interactions, which in eukaryotes may involve active protein complexes [7, 8] leading to translation blockade and the degradation or stabilization of the target messenger RNA (mRNA) [9]. sRNA:mRNA interactions may also lead to transcriptional or translational activation [10, 11].

sRNAs originate from multiple sources other than the intergenic regions of bacterial genomes [12]. Moreover, the 3’ untranslated region (UTR) of mRNAs emerged as a potential major reservoir of sRNAs [13–15]. Barquist and colleagues [16] have also introduced original evolutionary concepts on transcriptional noise and exaptation as sources of bacterial sRNAs.

With the unprecedented pace of technical advances and sRNA discovery [17, 18], the paradigm of their origin has been extended to two major classes of ncRNAs: rRNAs and tRNAs. Studies in eukaryotes revealed that rRNA fragments (rRFs) and tRNA fragments (tRFs) are not degradation products, but rather functional sRNAs with specific expression patterns and functions [19–22]. Lalaouna et al., [23] have also evidenced a functional bacterial tRF corresponding to an external transcribed spacer (ETS) which acts as a sponge for sRNAs to prevent transcriptional noise.

However, the literature on the existence, characteristics, biogenesis or function of tRFs in bacteria is scarce; the nomenclature not well established, and little is known about their potential role in microbial physiology and host-bacteria relationships, which is likely underappreciated [24]. The biogenesis of tRFs, their cell-autonomous effect, their transfer to host cells and the emergence of bacterial sRNAs as virulence factors in host-bacteria interactions were reviewed recently [25–27].

In bacteria-host interactions that occur in large interface areas, such as the colon, where the bacterial load exceeds other organs by two orders of magnitude [28], it is assumed that the microbiome employs immunostimulatory mechanisms capable of activating the host immune system and afferent pathways [29]. However, these interactions are often seen from the perspective of macromolecules [30], overlooking *de facto* the potential role of small RNAs and that of strategic carriers like outer membrane vesicles (OMVs).

Bacterial extracellular vesicles such as OMVs (50–250 nm) are spherical particles produced and released in all domains of life. These extracellular particles are rich in active biomolecules and perform multiple intercellular functions [31]. OMVs also constitute an attractive "type zero" secretion system capable of delivering their contents to the host without directly exposing the bacteria from which they originate [32]. The RNA content of bacterial OMVs has been long hypothesized [33] and was established much later [34–36] than that of extracellular vesicles derived from eukaryotic cells [37, 38]. The RNA of extracellular vesicles may have the potential to shape microbial communities and host–microbe interactions [39].

Beyond their profile, little is known about the biological role of bacterial RNAs once transferred into human host cells. Recent studies have described the first example of trans-kingdom biological activity of regulatory sRNAs contained in OMVs derived from *Pseudomonas aeruginosa* reference strain PA14 [40], *Legionella pneumophila* strain Paris [41] and periodontal pathogens [42].

Our group recently reported the discovery of abundant, unusually short (12–13 nt) and functional dodecaRNAs (doRNAs) mapping to rRNA 5.8S in eukaryotes [43]. In line with this work, we identified and characterized very small RNAs (vsRNAs) shorter than 16 nt using RNA-Sequencing (RNA-Seq) in *E*. *coli* and five other bacterial strains (*Pseudomonas aeruginosa* PA7, *P*. *aeruginosa* PAO1, *Salmonella enterica* serovar Typhimurium 14028S, *L*. *pneumophila* JR32 Philadelphia-1 and *Staphylococcus aureus* HG001). These vsRNAs were highly abundant in *E*. *coli* and their derived OMVs, with heterogeneous and distinct populations. The loading of *E*. *coli* vsRNAs, especially tRFs, in OMVs seemed to be selective. We also observed that tRFs are probably produced upon specific processing of tRNA species, form thermodynamically stable hairpin structures, and are predicted to target several host mRNAs with diverse functions [44].

**Fig 1:**
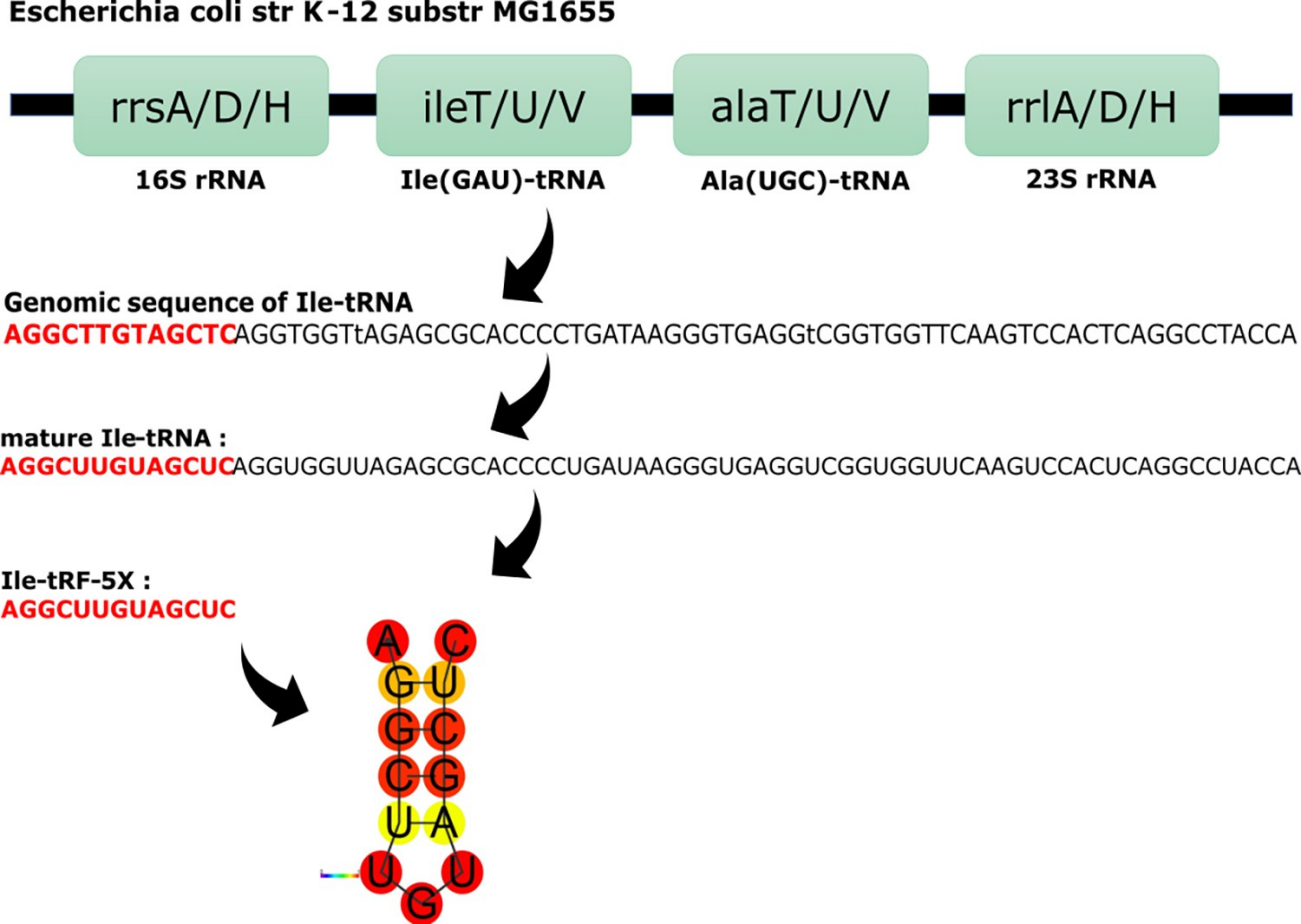
Simplified organization of the 3 operons containing the Ile-tRNA source of Ile-tRF-5X. *isoleucine*(Ile)-tRNA (GAT = anticodon) and *alanine*(Ala)-tRNA (TGC = anticodon) genes are found in the rrnA, rrnD and rrnH operons of *E*. *coli* K-12 genome. The Ile and Ala tRNAs are flanked by the 16S and 23s ribosomal RNAs (rRNAs). *ileT*, *ileU*, *ileV* genes all produce Ile(GAU)-tRNA. *alaT*, *alaU*, *alaV* genes all produce Ala(UGC)-tRNA. This diagram represents the three transcription units in one panel, under which the genomic sequence of Ile-tRNA and its mature sequence are presented. The 13-nt sequence in red represents Ile-tRF-5X, with its predicted secondary structure. The diagram does not show the endo and exonucleases involved in the maturation at each stage. The ribonuclease(s) involved in the cleavage of Ile-tRF-5X 3’ end have yet to be identified. Str, strain; substr, substrain; tRNA, transfer RNA.

We hypothesized that the dynamic host-gut microbiota interaction may involve an exchange mechanism based on OMVs and implicating the transfer of bacterial tRFs to human host cells, with possible implications in pathological and/or homeostasis processes. To test this hypothesis, we studied a tRF belonging to the 5’ end of the mature Isoleucine (Ile)-tRNA (tRF-5, **Fig 1**), which we found to be the most abundant tRF in both *E*. *coli* and their OMVs. The tRF-5s are divided into subclasses: tRF-5a (14–16 nts), tRF-5b (22–24 nts) and tRF-5c (28–30 nts) [45]. Our tRF-5 of interest being 13-nt long was tentatively named Ile-tRF-5X.

Here, we report that bacterial Ile-tRF-5X can be selectively modulated by environmental stress and, once delivered through OMVs to human host HCT116 cells, can regulate expression of a component of the mitogen-activated protein kinases (MAPKs) pathway through competition with miRNA silencing. We also show that OMVs and Ile-tRF-5X enhance proliferation of cultured colorectal carcinoma cells.

## Results

### Ile-tRF-5X levels in *E*. *coli* MG1655 are increased upon nutritional and thermal stresses

Before studying vsRNAs in the bacteria-host relationship, we characterized the level of vsRNA Ile-tRF-5X in reference strain *E*. *coli* MG1655 under different growth conditions. To this end, we designed and used a sensitive and specific RT-qPCR detection method based on splint ligation to monitor Ile-tRF-5X level. The technique is detailed in the Materials and methods section and illustrated in **S1A–S1C Fig**.

**Fig 2:**
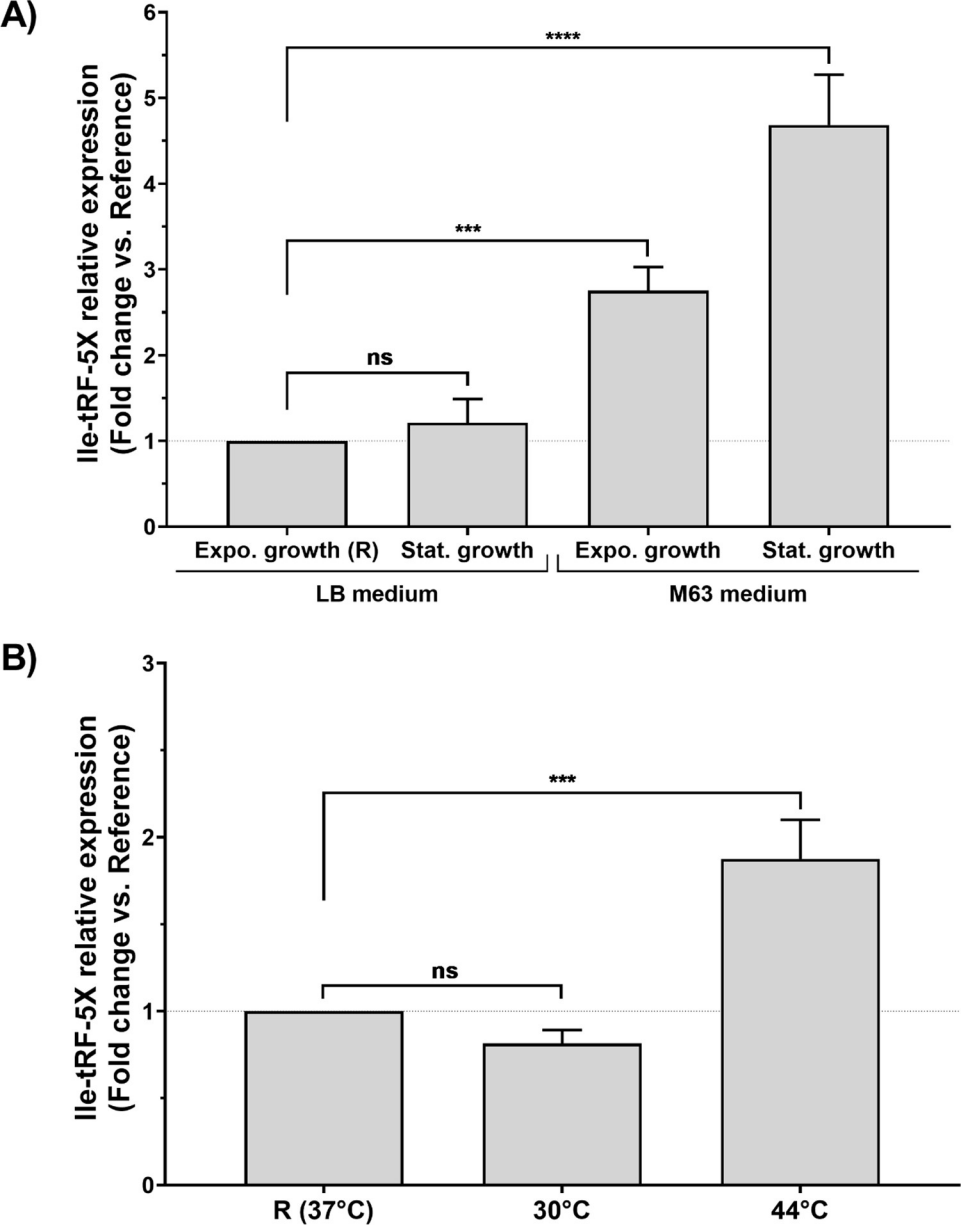
Bacterial Ile-tRF-5X levels under different growth and temperature conditions. (**A**) *E*. *coli* MG1655 bacteria were grown at 37°C, and the level of Ile-tRF-5X was monitored during the exponential and stationary phases of growth in either complete (rich) LB or minimal M63 medium. (**B**) *E*. *coli* MG1655 bacteria were grown in LB medium, and the level of Ile-tRF-5X was monitored during the exponential phase at 30, 37 and or 44°C (for details, see **S1 Table and S2 Fig**). The level of Ile-tRF-5X was measured by LNA RT-qPCR. A spike-in (UniSp6) and reference genes (23S and/or 16S) were used as control and for normalization. The results are reported in fold change compared to the reference condition. **Statistical analysis**. Data were calculated from three biological replicate measurements (n = 3; mean ± SD). One-way analysis of variance (ANOVA) and Holm-Šídák’s multiple comparisons test (post-hoc test) were used for statistical analysis. Statistically significant differences (fold change vs. reference) are indicated by stars (*), * p < 0.05; ** p < 0.01; *** p < 0.001; **** p < 0.0001; ns, not significant. In the M63 medium, the difference between the exponential and stationary phase was statistically significant (***p = 0.0006).

First, we evaluated the level of Ile-tRF-5X expression at the exponential and stationary phases of bacterial growth (**Fig 2A**). In a rich and complete LB medium, although the mature Ile-tRNA appeared to accumulate in the stationary phase (about 3-fold more than Ile-tRF-5X; **S2 Fig**), Ile-tRF-5X level seemed not to be affected by the growth phase, as suggested also by our RNA-Seq data (see **S3 Fig).**

However, in low nutrient culture medium (M63), the level of Ile-tRF-5X was significantly increased in both exponential (by 2-fold) and stationary (by 4-fold) phases compared to exponential growth phase in LB medium, chosen as a reference (**Fig 2A**). Under these respective conditions, the Ile-tRNA was reduced by 20 (3-fold less than Ile-tRF-5X) and 60% (11-fold less than Ile-tRF-5X) compared to the reference (**S2 Fig**).

Next, we investigated the level of Ile-tRF-5X in function of temperature (**Fig 2B**) at 30°C and 44°C, in comparison with exponential growth at the optimal reference temperature of 37°C. Compared to the reference, the level of Ile-tRF-5X significantly increased by 2-fold when raising the temperature to 44°C (+7°C), but remained unchanged when the temperature was lowered to 30°C (-7°C). The level of mature Ile tRNA followed the same pattern as Ile-tRF-5X at both temperatures tested (**S2 Fig)**

Therefore, the level of Ile-tRF-5X appears to be modulated by two major growth conditions (i.e., nutrients availability and temperature) encountered by bacteria.

### Ile-tRF-5X is not affected by inhibition of transcription or translation

We then assessed whether the level of Ile-tRF-5X could be related to transcription or translation, for which we have used rifampicin and chloramphenicol, respectively. Rifampicin inhibits the bacterial RNA polymerase (RNAP) and stops global RNA synthesis at the initiation of transcription, while having no effect on replication elongation [46]. Chloramphenicol, on the other hand, has no effect on transcription and rather targets the 50S ribosomal subunit and inhibits peptidyl transferase activity, resulting in the cessation of protein synthesis [47].

**Fig 3:**
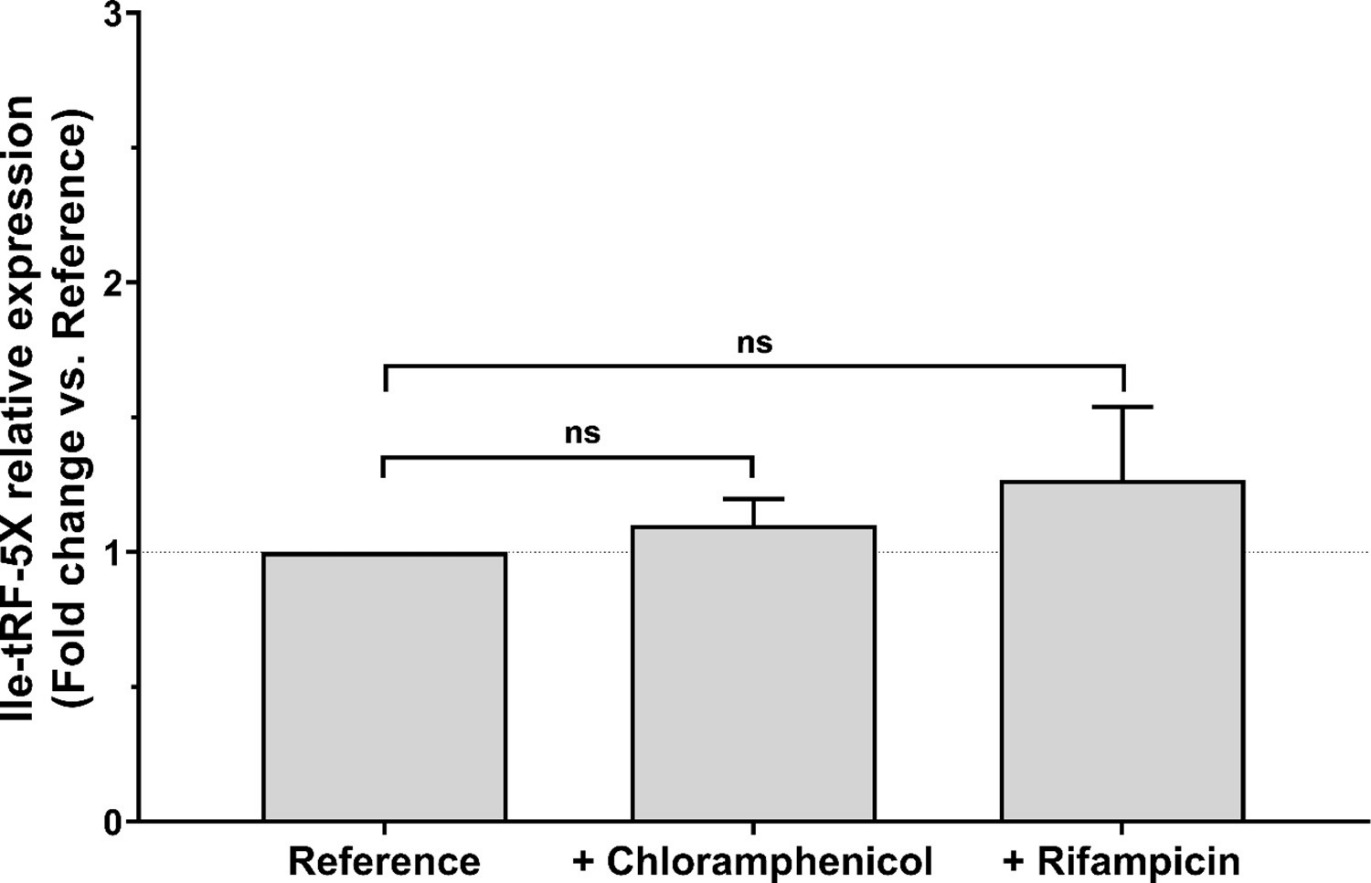
The level of bacterial Ile-tRF-5X level is not modulated by transcription or translation activity. Bacterial mRNA or protein synthesis was inhibited by addition of chloramphenicol or rifampicin, respectively, to cultures of *E*. *coli* MG1655 at 37°C (for details, see **S1 Table and S2 Fig**). The level of Ile-tRF-5X was measured by LNA RT-qPCR. A spike-in (UniSp6) and reference genes (23S and/or 16S) were used as control and for normalization. The results are reported in fold change compared to the reference condition. **Statistical analysis**. Data were calculated from three biological replicate measurements (n = 3; mean ± SD). One-way analysis of variance (ANOVA) and Holm-Šídák’s multiple comparisons test (post-hoc test) were used for statistical analysis. Statistically significant differences (fold change vs. reference) are indicated by stars (*), * p < 0.05; ** p < 0.01; *** p < 0.001; **** p < 0.0001; ns, not significant.

Inhibition of RNA and protein synthesis, with rifampicin and chloramphenicol respectively, did not affect the level of bacterial Ile-tRF-5X (**Fig 3**). These RT-qPCR results confirm our RNA-Seq data suggesting that the level of bacterial Ile-tRF-5X is not affected by inhibition of transcription or translation (**S3 Fig**).

A complementary RT-qPCR analysis (**S2 Fig**) showed that the level of Ile-tRNA was affected by inhibition of RNA (20% decrease) and protein synthesis (70% increase), suggesting a lack of correlation between the level of tRNAs and that of tRFs.

### Regulation of Ile-tRF-5X levels may involve RNase E, but not RNase P

**Fig 4:**
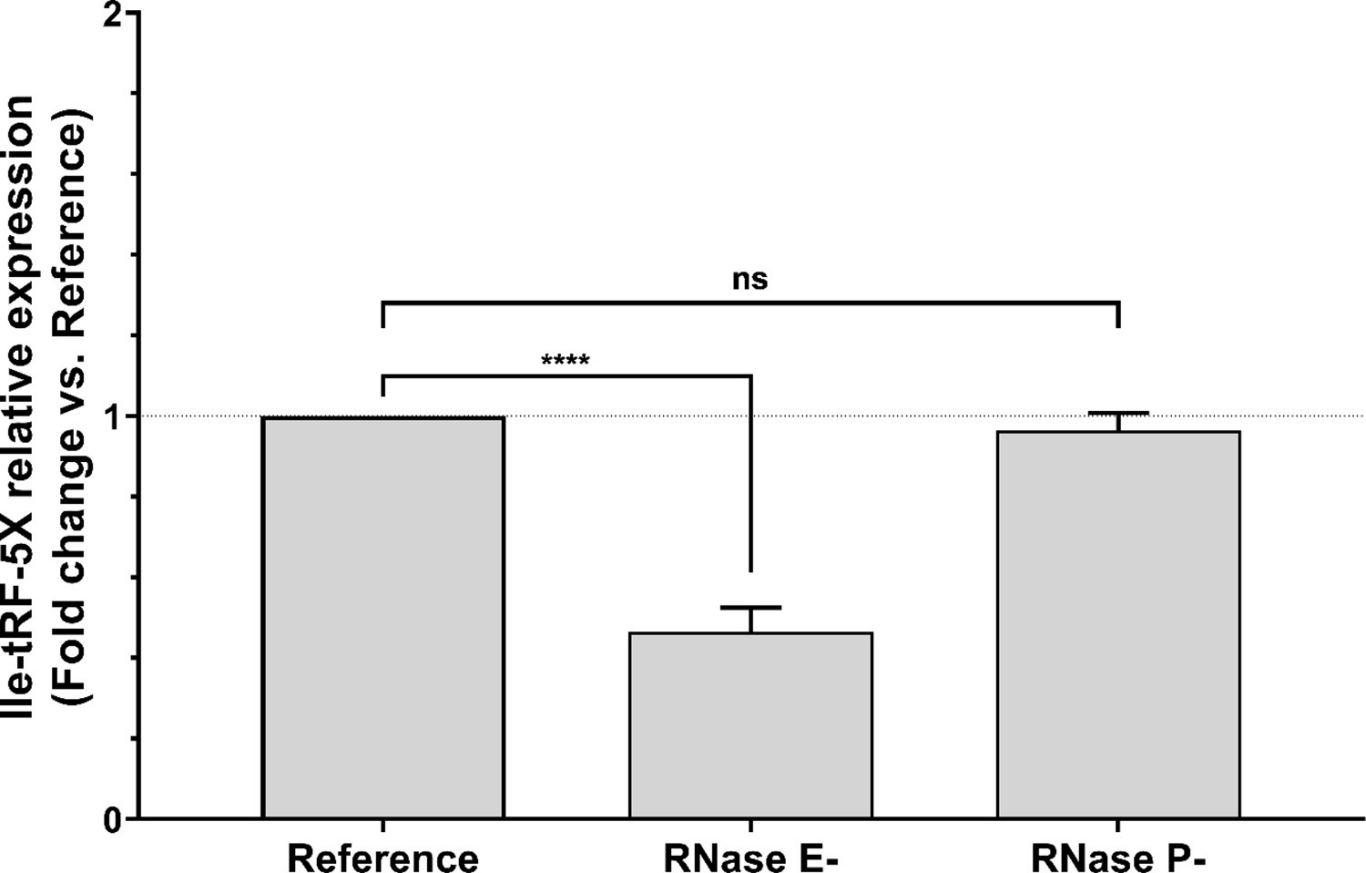
Bacterial RNase E contributes to Ile-tRF-5X biogenesis. *E*. *coli* strains carrying heat-sensitive (hs) mutations in the essential genes *rne-3071-hs* (EM1277) and *rnpA-hs* (KP1036) were grown in LB medium at 30°C and then heat-shocked to transiently inhibit ribonuclease (RNase) P or RNase E, which are involved in tRNA maturation (for details, see **S1 Table and S2 Fig**). The level of Ile-tRF-5X was measured by LNA RT-qPCR. A spike-in (UniSp6) and reference genes (23S and/or 16S) were used as control and for normalization. The results are reported in fold change compared to the reference condition. **Statistical analysis**. Data were calculated from three biological replicate measurements (n = 3; mean ± SD). One-way analysis of variance (ANOVA) and Holm-Šídák’s multiple comparisons test (post-hoc test) were used for statistical analysis. Statistically significant differences (fold change vs. reference) are indicated by stars (*), * p < 0.05; ** p < 0.01; *** p < 0.001; **** p < 0.0001; ns, not significant.

The intriguing invariability of Ile-tRF-5X levels following inhibition of transcription or translation led us to scrutinize its biosynthesis. To do so, the level of Ile-tRF-5X expression was assessed following inhibition of some key enzymes of the tRNA maturation pathway (**Fig 4**).

tRNA processing involves several ribonucleases, such as RNase E and RNase P [48]. Involved in almost all aspects of RNA metabolism, RNase E initiates tRNA maturation and provides substrates to RNase P and other exoribonucleases [49]. RNase P, which exists in all three kingdoms of life, generates the mature 5’ ends of tRNAs [50].

Transient and thermosensitive inhibition of RNase E at 44°C decreased the level of Ile-tRF-5X by half compared to the reference condition, as suggested by our RNA-Seq data (**S3 Fig**). These findings imply an active participation of RNase E in the biosynthesis of vsRNA Ile-tRF-5X. However, inhibition of RNase E did not lower the level of mature Ile-tRNA from which Ile-tRF-5X originated. Ile-tRNA was 30% more abundant than the control and 3-fold more abundant than Ile-tRF-5X (**S2 Fig**).

RNase P, on the other hand, does not seem to be involved in the processing of Ile-tRF-5X, as inhibition of this key tRNA maturation enzyme did not alter Ile-tRF-5X expression. This result leaves open the possibility that Ile-tRF-5X may originate from the mature and/or precursor tRNA. Unlike RNase E, the absence of RNase P did not significantly remodulate the level of Ile-tRNA (**S2 Fig**).

Taken together, these results suggest that Ile-tRF-5X is not only modulated by growth conditions, but also by general RNA metabolism mediated by RNase E. Ile-tRF-5X might therefore be considered as a product of a specific process.

### Bacterial OMVs are internalized by HCT116 cells

We have previously shown that a group of thermodynamically stable bacterial vsRNAs, including Ile-tRF-5X (**Fig 1**), were selectively enriched and loaded into OMVs ([44]; **S4 Fig**), suggesting a potential role in bacteria-host cell communications.

**Fig 5:**
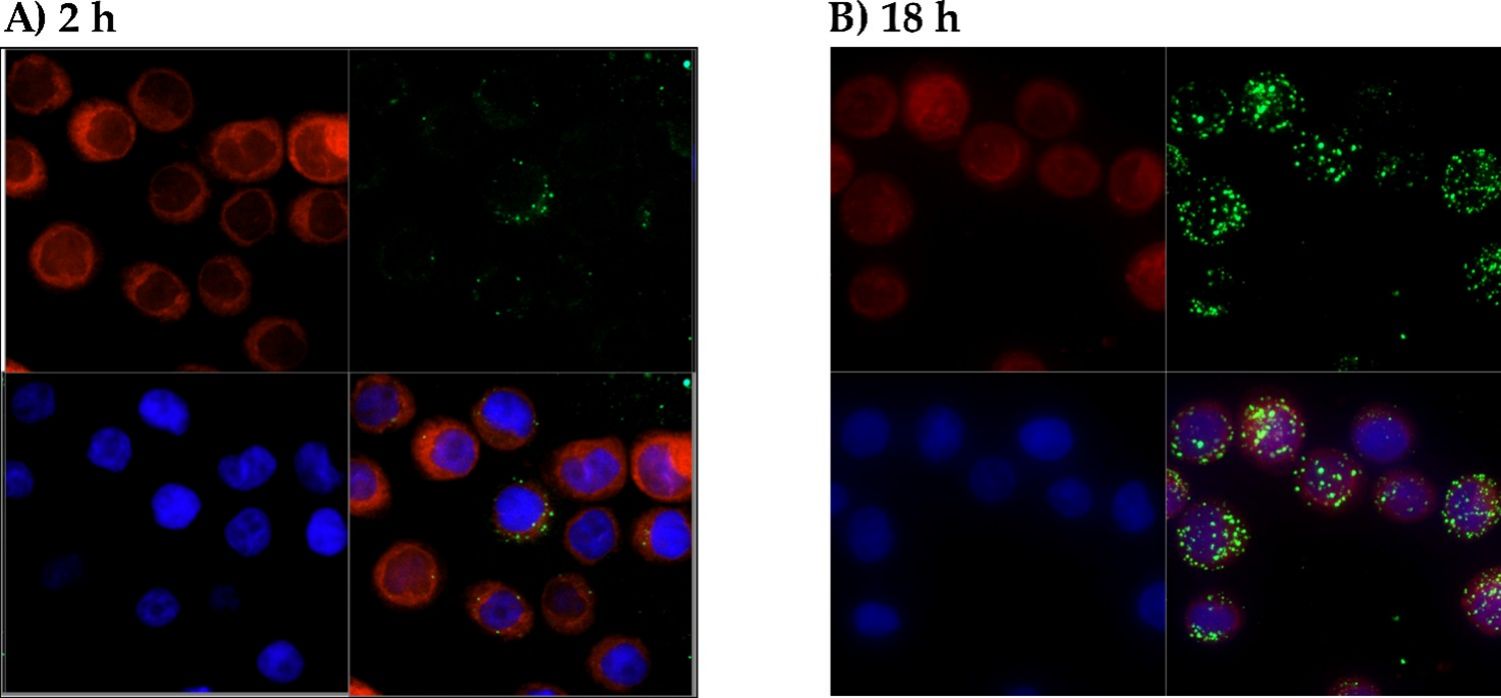
Human HCT116 cells internalize bacterial OMVs. Confocal microscopy imaging of human HCT116 cells stained with cell tracker CMTPX (in red) and incubated with bacterial OMVs labelled with PKH67 (in green) for 2 h (**A**) or 18 h (**B**). Nuclei were stained with DAPI (in blue). The XZ and YZ projection of volocity shows that bacterial OMVs were internalized by HCT116 cells and localized mainly to the cytoplasm (see **S5 Fig**).

To investigate that possibility, we performed a confocal fluorescence microscopy analysis of labeled human colorectal carcinoma cell line HCT116 incubated with PKH67-labeled bacterial OMVs (**Fig 5**). Volocity’s XZ and XZ projection features were used to verify the localization (membrane, cytoplasm, nuclei, etc.) of fluorophores (**S5 Fig**).

After 2 h of incubation, a small proportion of bacterial OMVs were already found inside the cells, while the majority were still located on the cell surface. After 18 h of incubation, most of the bacterial OMVs were internalized and scattered in the cytoplasm. Some cells did not take up bacterial OMVs, suggesting a selective adhesion and internalization process.

### Bacterial Ile-tRF-5X is transferred to human HCT116 cells

After demonstrating that bacterial OMVs could be internalized by human host cells, we verified whether their content, especially Ile-tRF-5X, which is the most abundant vsRNA, could be transferred to host cells.

**Fig 6:**
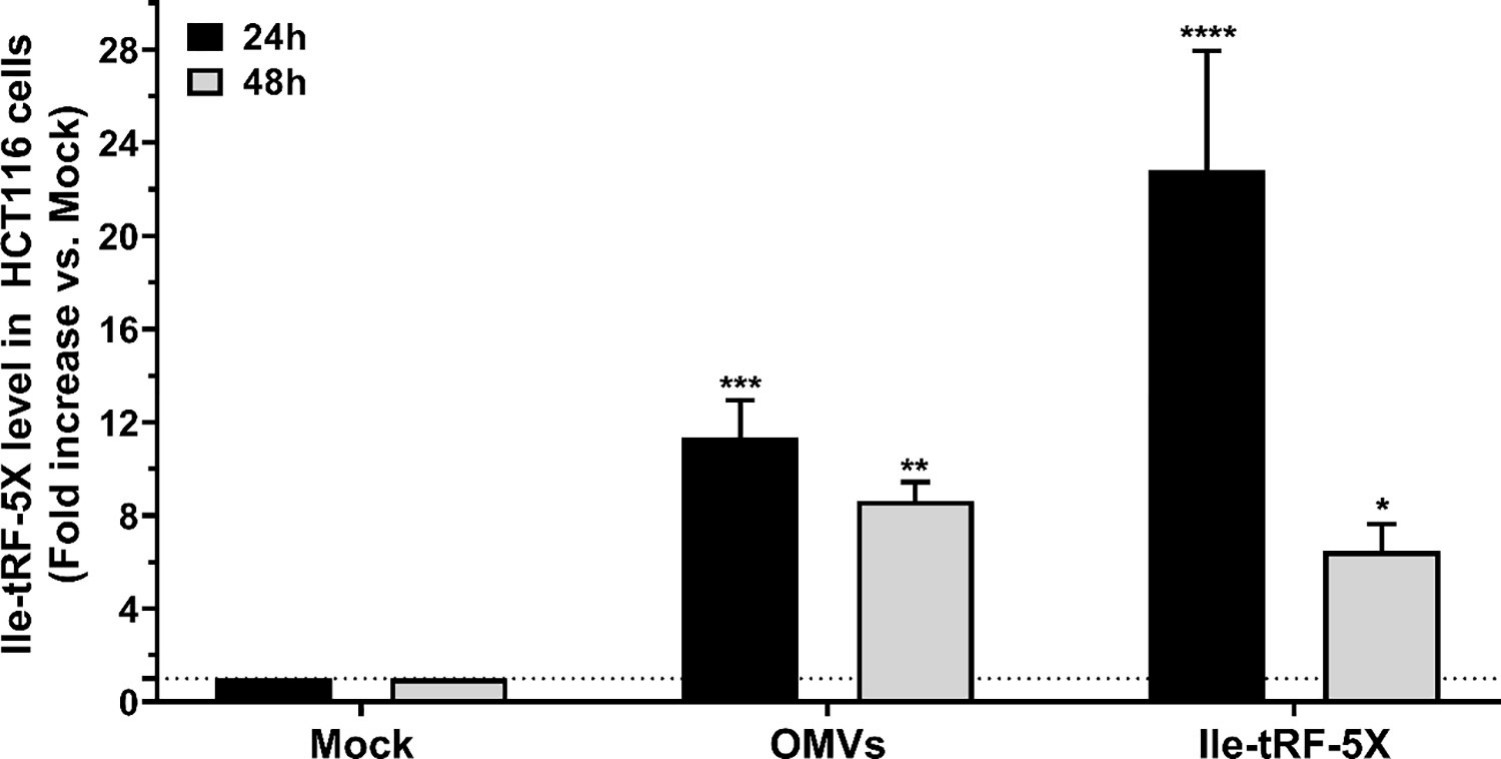
Bacterial OMVs deliver Ile-tRF-5X to human HCT116 cells. Human HCT116 cells were incubated with (10 ng) *E*. *coli* MG1655-derived OMVs or transfected with an equivalent amount of synthetic Ile-tRF-5X. The level of Ile-tRF-5X was measured at 24 h and 48 h by LNA RT-qPCR. UniSp6 was used as spike-in control and reference gene for normalization. The results were reported in fold change compared to the corresponding mock condition. **Statistical** analysis. Data were calculated from three biological replicate measurements (n = 3; mean ± SD). One-way analysis of variance (ANOVA) and Holm-Šídák’s multiple comparisons test (post-hoc test) were used for statistical analysis. Statistically significant differences is indicated as follows: * p < 0.05; ** p < 0.01; *** p < 0.001; **** p < 0.0001.

We monitored, by LNA RT-qPCR, the level of bacterial Ile-tRF-5X in HCT116 cells upon exposure to bacterial OMVs or synthetic Ile-tRF-5X (**Fig 6**).

HCT116 cells exposed to OMVs displayed high levels of Ile-tRF-5X at 24 h, which dropped by a third at 48 h, but remained significant. Cells transfected with synthetic Ile-tRF-5X displayed a marked increase in Ile-tRF-5X levels, compared to mock-transfected cells, which were two times higher than cells exposed to OMVs at 24 h. At 48 h, the level of Ile-tRF-5X dropped drastically by 74% compared to its level at 24 h. At 48 h, a third more Ile-tRF-5X was found in cells incubated with OMVs compared to those transfected with synthetic Ile-tRF-5X.

These results indicate that bacterial OMVs and their Ile-tRF-5X content can be transferred to human host cells.

### Bacterial OMVs promote MAP3K4 expression

Whether bacterial Ile-tRF-5X can function in human host cells and regulate host gene expression was initially explored using bioinformatic tools, which brought to light several host mRNAs that might be targeted by tRF vsRNAs, like Ile-tRF-5X [44]. Envisioning these interactions on large host-bacteria exchange surfaces such as the colon, we focused on related targets previously reported in this context and related to extracellular signal transduction, inflammatory cytokines, or environmental stress. For further validation, and as a proof of concept of the functionality of Ile-tRF-5X, we retained Mitogen-activated protein kinase 3 mRNA (MAP3K4, also known as MEKK4), which carries no less than 10 potential binding sites, including one with perfect complementarity to Ile-tRF-5X located in the open reading frame (ORF) of MAPK3 (**S6 Fig**). Involved in several aspects of cellular regulation, MAPKs are important signal transducing enzymes which allow the transfer of signals from cell surface receptors to critical regulatory targets within cells [51].

**Fig 7:**
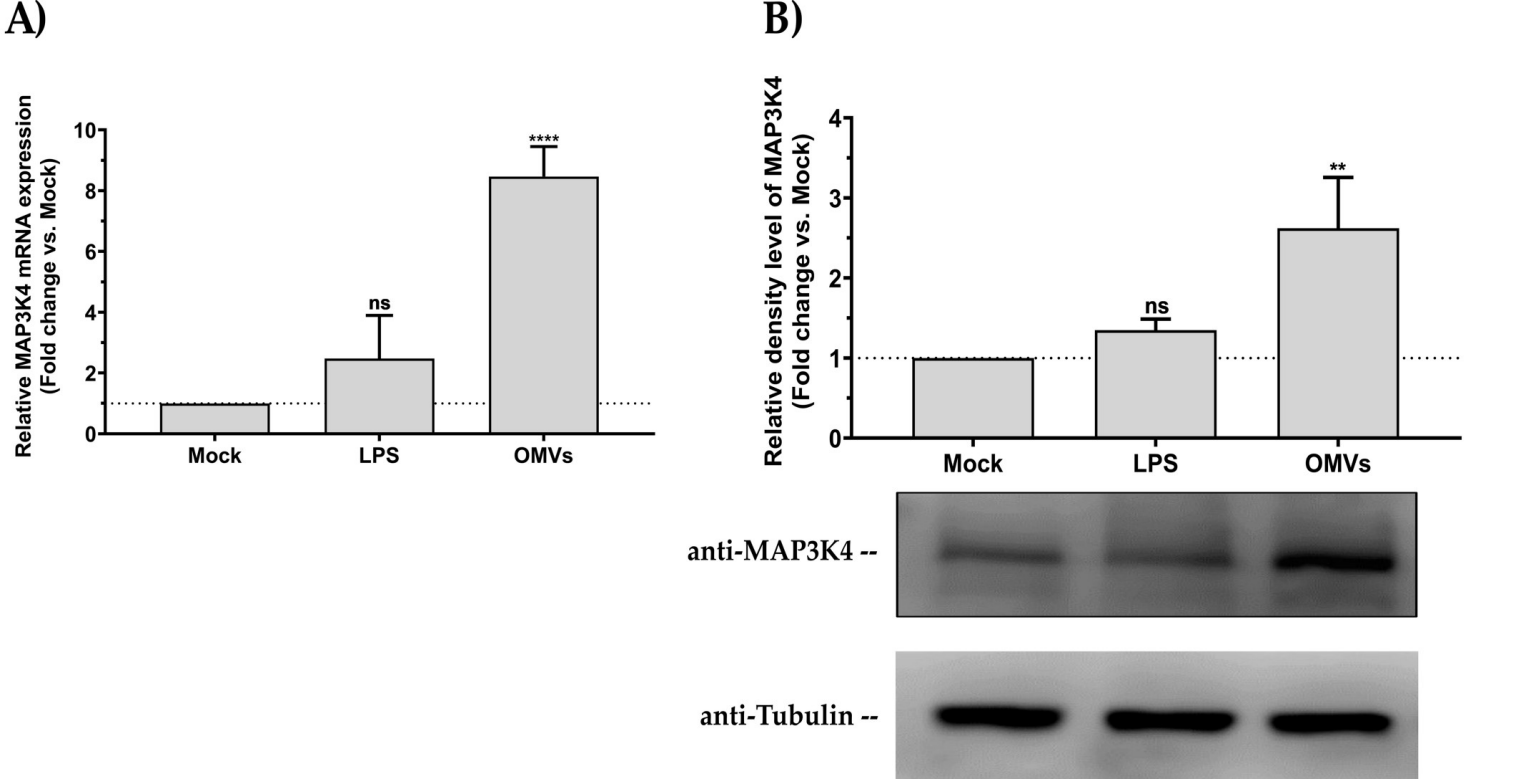
Bacterial OMVs enhance human MAP3K4 expression. Human HCT116 cells were incubated with (10 ng) *E*. *coli* MG1655-derived OMVs or an equivalent amount of LPS, compared to a mock control. (**A**) Relative quantification of MAP3K4 mRNA expression by RT-qPCR. Data were normalized with a reference gene (ACTB), reported as fold change vs mock control, and expressed with the relative quantitation method (ΔΔCt). (**B**) Western blot analysis of MAP3K4 protein expression relative to tubulin. Densitometric analysis of the bands was performed with ImageJ. Data were normalized with a reference gene (tubulin) and expressed as fold change vs mock control. **Statistical analysis.** Data were calculated from three biological replicate measurements (n = 3; mean ± SD). One-way analysis of variance (ANOVA) and Holm-Šídák’s multiple comparisons test (post-hoc test) were used for statistical analysis. Statistically significant differences (fold change vs mock) are indicated as follows: ** p < 0.01; **** p < 0.0001. ns, not significant.

We first exposed HCT116 cells to bacterial OMVs and measured the level of MAP3K4 expression. As OMVs naturally contain pathogen-associated molecular patterns (PAMPs), among which lipopolysaccharide (LPS) remains one of the major components, LPS was used as a control. The results showed that bacterial OMVs enhanced MAP3K4 expression at both messenger RNA and protein levels (**Fig 7**). Incubation of HCT116 cells with LPS did not alter MAP3K4 expression.

### Bacterial Ile-tRF-5X induces sequence-specific upregulation of MAP3K4 mRNA expression

To ascertain that the observed changes in MAP3K4 levels upon exposure to bacterial OMVs can be attributed to Ile-tRF-5X and not to other OMV components, we transfected HCT116 cells with either Ile-tRF-5X alone, a control sequence of the length of Ile-tRF-5X or small RNA (<200 nt) isolated from OMVs.

**Fig 8:**
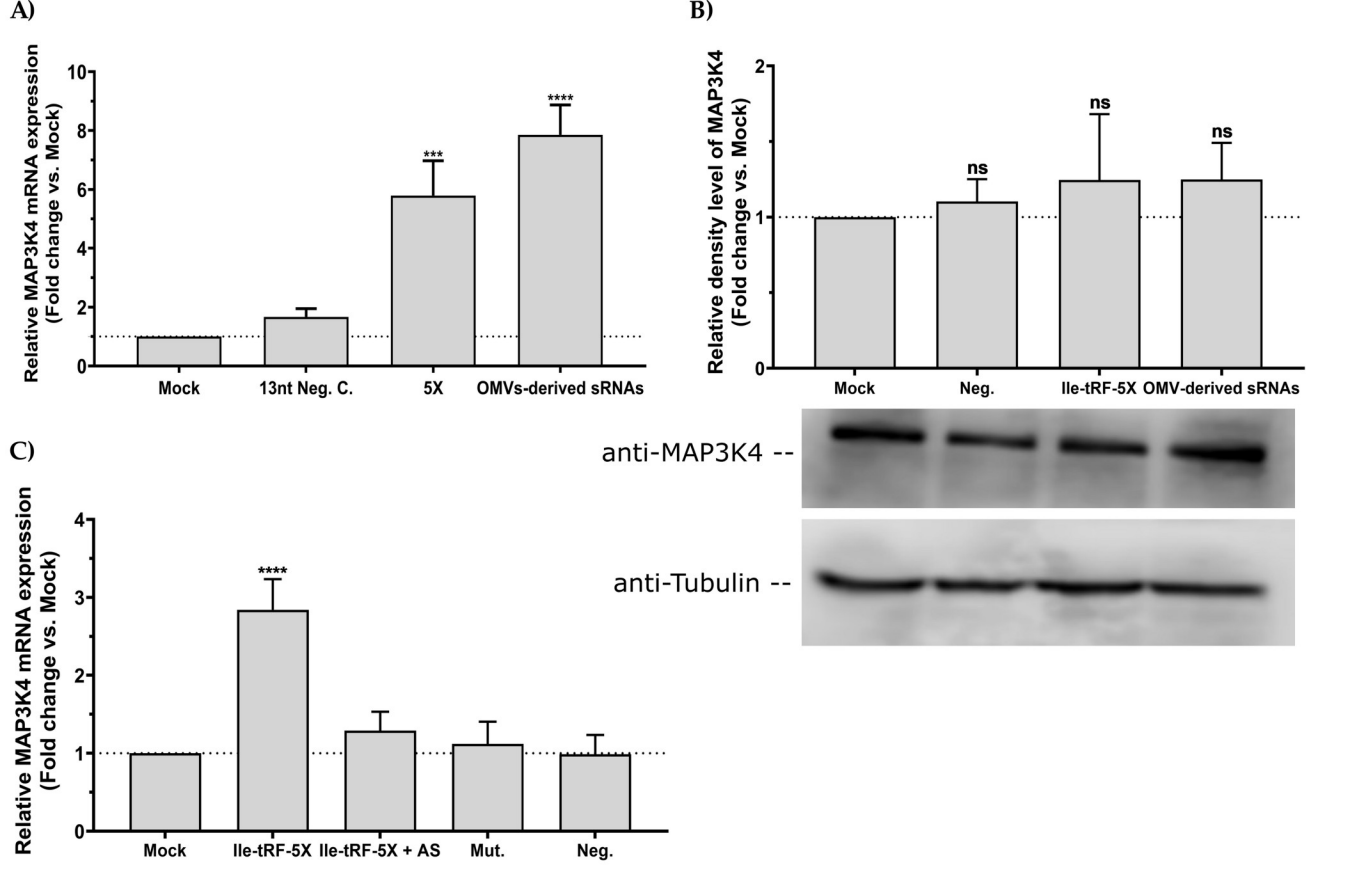
Bacterial Ile-tRF-5X upregulates human MAP3K4 expression in a sequence specific manner. (**A-C**) Human HCT116 cells were transfected with (100 nM) synthetic Ile-tRF-5X alone, mutated (Mut.) or combined with its antisense (AS), unrelated 13-nt negative control (Neg.) or small RNAs (sRNAs) derived from bacterial OMVs. (**A**) and (**C**) Relative quantification of MAP3K4 mRNA expression by RT-qPCR. Data were normalized with a reference gene (ACTB), reported as fold change vs mock control, and expressed with the relative quantitation method (ΔΔCt). (**B**) Western blot analysis of MAP3K4 protein expression relative to tubulin. Densitometric analysis of the bands was performed with ImageJ. Data were normalized with a reference gene (tubulin) and expressed as fold change vs mock control. **Statistical analysis**. Data were calculated from three biological replicate measurements (n = 3; mean ± SD). One-way analysis of variance (ANOVA) and Holm-Šídák’s multiple comparisons test (post hoc test) were used for statistical analysis. Statistically significant differences are indicated as follows: *** p < 0.001, **** p < 0.0001. ns, not significant.

Ile-tRF-5X seemed to upregulate MAP3K4 only at the mRNA level (**Fig 8A and 8B**).

Interestingly, the cocktail of small OMV-derived RNAs (<200 nt) also appeared to modulate MAP3K4 expression, slightly more than Ile-tRF-5X alone.

To address the specificity of the interplay between Ile-tRF-5X and MAP3K4, a mutated version of Ile-tRF-5X was also tested as well as an antisense to Ile-tRF-5X. Mutated Ile-tRF-5X had no effect and the antisense neutralized the MAP3K4 gene regulatory effects of Ile-tRF-5X, supporting the sequence-specificity of the interaction between Ile-tRF-5X with MAP3K4 mRNA (**Fig 8C**).

### Bacterial OMVs and Ile-tRF-5X modulate human MAP3K4 expression through the miRNA pathway

The presence of putative binding sites to Ile-tRF-5X in MAP3K4 mRNA (**S6 Fig**) led us to speculate that bacterial vsRNAs may function like eukaryotic miRNAs or tRFs, i.e. through binding and downregulation of their mRNA targets [52]. However, the opposite upregulation of MAP3K4 levels induced by Ile-tRF-5X suggests that it might not follow this mechanism of action. Instead, we purported that vsRNAs might compete with human host miRNAs and/or tRFs involved in MAP3K4 gene regulation.

Both tRFs and miRNAs largely depend on the ribonuclease Dicer for their production, but the high variability of the mechanisms involved in tRF generation led us to focus on miRNAs. To probe the modulation of MAP3K4 expression, we thus used a Dicer-deleted (Dicer -/-) HCT116 cell line, which exhibit a 96% depletion of mature miRNA levels [53], in parallel to the wild-type HCT116 cell line.

**Fig 9:**
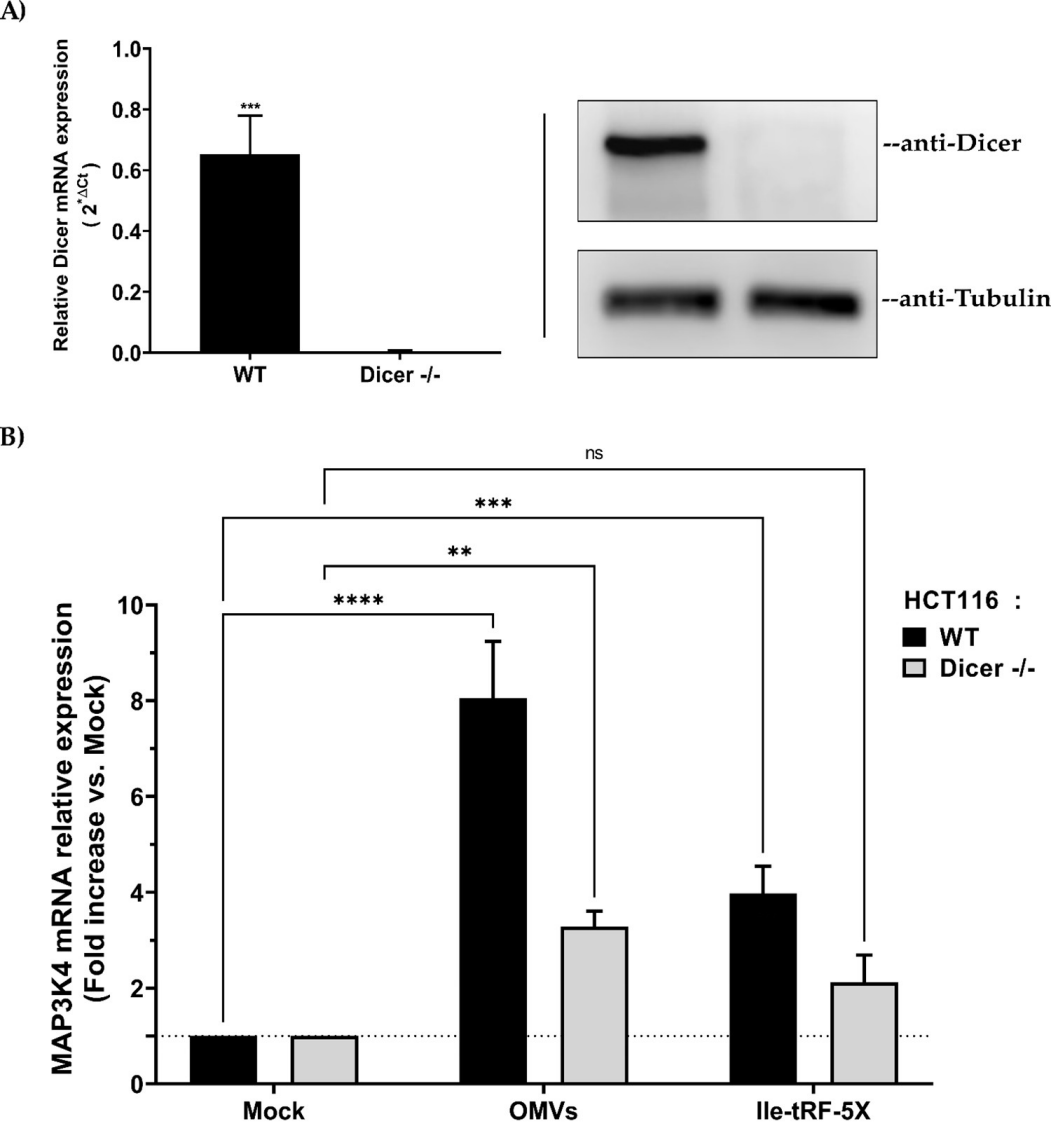
Bacterial OMVs and Ile-tRF-5X modulate human MAP3K4 expression through the miRNA pathway. (**A**) Dicer deletion (Dicer -/-) in human HCT116 cells was confirmed at the mRNA level by qPCR and at the protein level by Western blot using anti-Dicer and anti-Tubulin (control) antibodies. (**B**) Relative MAP3K4 mRNA expression was quantified at 48h by RT-qPCR in either wild-type (WT) or Dicer -/- HCT116 cells after incubation with bacterial OMVs (10 ng) or transfection with Ile-tRF-5X (100 nM). Data were normalized with a reference gene (ACTB), reported as fold change vs mock control, and expressed with the relative quantitation method (ΔΔCt). **Statistical analysis**. Data were calculated from three biological replicate measurements (n = 3; mean ± SD). Unpaired Student’s t test (**A**) and two-way analysis of variance (ANOVA) and Holm-Šídák’s multiple comparisons test (**B**) were used for statistical analysis. Statistically significant differences (fold change vs. mock) are indicated as follows: * p < 0.05; *** p < 0.001; **** p < 0.0001.

First, we confirmed the depletion of human Dicer at the mRNA and protein levels in Dicer -/- HCT116 cells, compared to wild-type cells (**Fig 9A**). These cells were then incubated in the absence or presence of bacterial OMVs or transfected or not with synthetic Ile-tRF-5X, followed by measurement of MAP3K4 mRNA expression. In HCT116 WT, either with OMVs or Ile-tRF-5X alone, there was an increase in the level of MAP3K4 whereas in the experimental condition where Dicer was absent, only OMVs induced a significant upregulation of MAP3K4 (**Fig 9B**). The respective 8- and 4-fold increase in MAP3K4 mRNA expression induced by bacterial OMVs or Ile-tRF-5X alone in wild-type HCT116 cells was reduced by half upon Dicer depletion (**Fig 9B**).

These results suggest that the MAP3K4 gene regulatory effects of bacterial OMVs or Ile-tRF-5X are, at least partially, mediated by Dicer, either directly (Dicer itself) or indirectly, through its miRNA products.

### Bacterial Ile-tRF-5X counteracts the miRNA-induced gene downregulatory properties of MAP3K4 3’UTR

As the regulatory elements recognized by miRNAs are usually located in mRNA 3’UTRs [54], we inspected the MAP3K4 mRNA 3’UTR for miRNA binding sites, or miRNA response elements (MREs), using TargetScan [55] and for the five most important potential Ile-tRF-5X binding sites using RNAhybrid [56].

We observed a close proximity between the MREs and those of Ile-tRF-5X (**S7 Fig**).

At least two of the MREs in MAP3K4 mRNA 3’UTR overlap with Ile-tRF-5X binding sites (at nt positions 77 and 208). The other Ile-tRF-5X binding sites were located within 30 nt of MREs, with one within 3 nt (nt position 121). The secondary structure of MAP3K4 mRNA 3’UTR, depicted by the Forna (RNAfolder) web interface [57], revealed that Ile-tRF-5X binding sites were mainly located in structured sequences, often in stem loops that made them even closer to MREs (**S8 Fig**).

**Fig 10:**
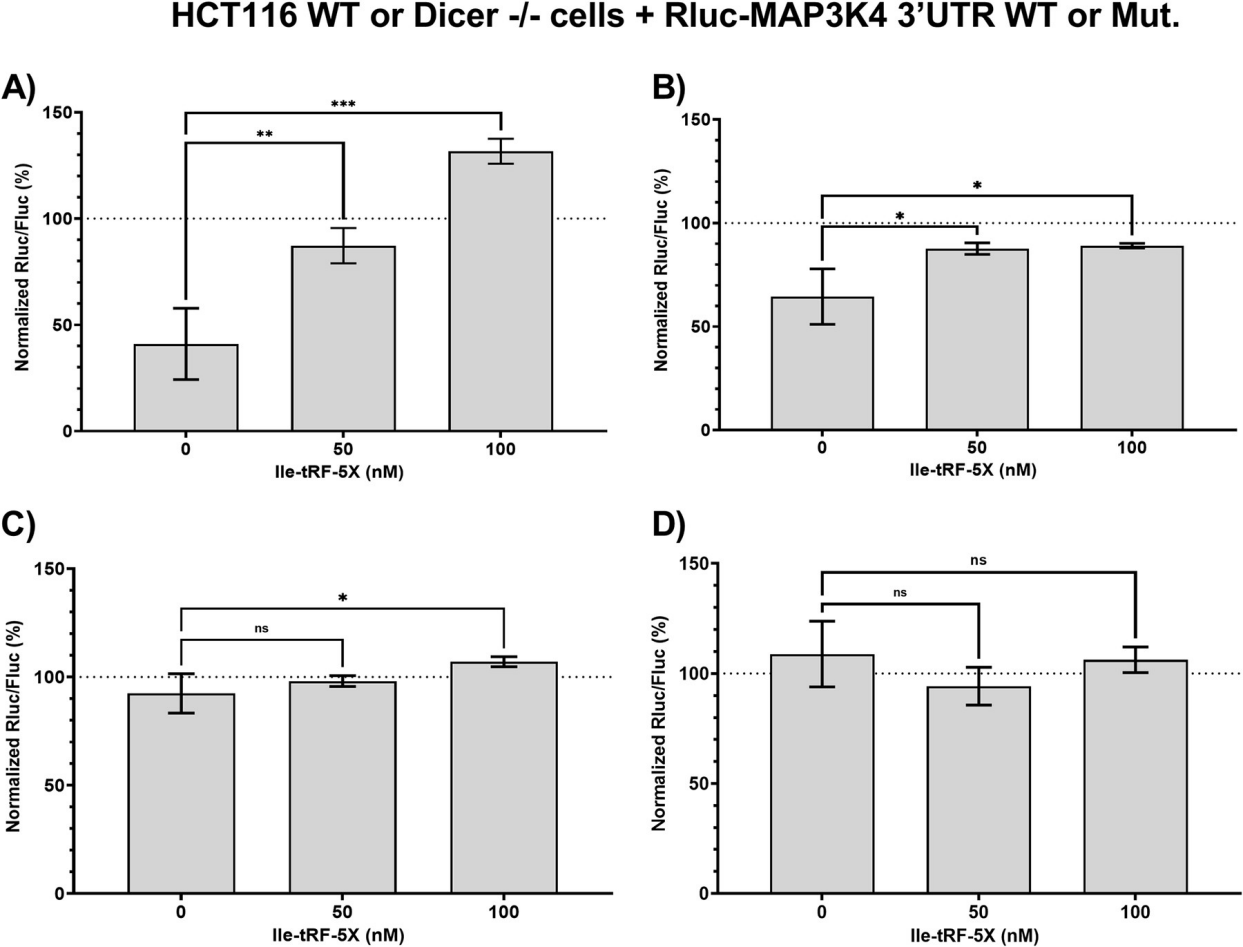
Bacterial Ile-tRF-5X counteracts the miRNA-induced gene downregulatory properties of MAP3K4 3’UTR. (**A-D**) Human HCT116 cells either wild-type (WT; **A-B**) or Dicer -/- (**C-D**) were co-transfected with synthetic Ile-tRF-5X (0, 50, 100 nM) and a psiCHECK-II reporter construct (50 ng; see S7 Fig) in which the Rluc reporter gene was coupled with WT (A, C) or mutated (Mut., B, D) MAP3K4 3’UTR. The details of the applied mutations are indicated in the S1 Information. An unrelated 13-nt sequence (100 nM) was used as control (0 nM Ile-tRF-5X). Dual luciferase reporter gene activity assays were performed as described previously [54], with Fluc as a normalization reporter control. **Statistical analysis**. Data were calculated from three biological replicate measurements (n = 3; mean ± SD). One-way analysis of variance (ANOVA) and Holm-Šídák’s multiple comparisons (post-hoc test) were used for statistical analysis. Statistically significant differences are indicated as follows: * p < 0.05; ** p < 0.01; *** p < 0.001. ns, not significant.

To study the possible interactions between miRNAs and Ile-tRF-5X in the regulation of MAP3K4 mRNA, we cloned the entire MAP3K4 mRNA 3’UTR into a dual-luciferase reporter gene system, which was used in wild-type and Dicer -/- HCT116 cells (**Fig 10**).

Introduction of the MAP3K4 mRNA 3’UTR downstream of Rluc reduced reporter gene expression in wild-type HCT116 cells by 60% compared to the empty vector (in the absence of added Ile-tRF-5X; **Fig 10A**), suggesting the presence of gene downregulatory elements in the 3’UTR. Addition of Ile-tRF-5X restored Rluc activity in a dose-dependent manner, supporting the ability of Ile-tRF-5X to counteract the gene downregulatory factors acting via the MAP3K4 mRNA 3’UTR (**Fig 10A**).

To determine the specificity of the Ile-tRF-5X gene regulatory effects, we performed the same experiment with mutated Ile-tRF-5X binding sites, without altering MREs. Rluc reporter gene activity was reduced by only 35% (**Fig 10B**), compared to as much as 60% for the wild-type MAP3K4 mRNA 3’UTR (**Fig 10A**), suggesting that the Ile-tRF-5X binding site mutations collaterally affected the inhibitory elements of the 3’UTR (**Fig 10B**). Addition of Ile-tRF-5X was still able to restore Rluc activity, but at a reduced intensity and without a dose-response effect, suggesting a role for Ile-tRF-5X binding sites in mediating its gene regulatory effects.

Expression of the Rluc reporter gene, placed under the control of the MAP3K4 mRNA 3’UTR, was reduced by only 10% in miRNA-depleted, Dicer -/- HCT116 cells, compared to 60% in wild-type HCT116 cells, which is compatible with the loss of miRNA-induced downregulation through the MAP3K4 mRNA 3’UTR. In cells depleted of miRNAs, addition of Ile-tRF-5X alleviated Rluc reporter gene expression by a marginal, but statistically significant, 15% at the highest concentration (**Fig 10C**). These small gene regulatory effects of Ile-tRF-5X were lost upon mutation of the Ile-tRF-5X binding sites (**Fig 10D**).

Taken together, these results suggest that bacterial Ile-tRF-5X may modulate the gene downregulatory properties of human MAP3K4 3’UTR, which may occur through competition with Dicer-derived miRNAs. Ile-tRF-5X does not seem to exert gene downregulatory effects through the MAP3K4 mRNA 3’UTR.

### Bacterial OMVs and Ile-tRF-5X dose-dependently enhance human cell proliferation

Since the mitogen-activated protein kinase (MAPK) pathways are known to connect extracellular signals to fundamental intracellular processes [58], we investigated if the upregulation of MAP3K4 triggered, directly or indirectly, cell proliferation, inflammation or apoptosis.

**Fig 11:**
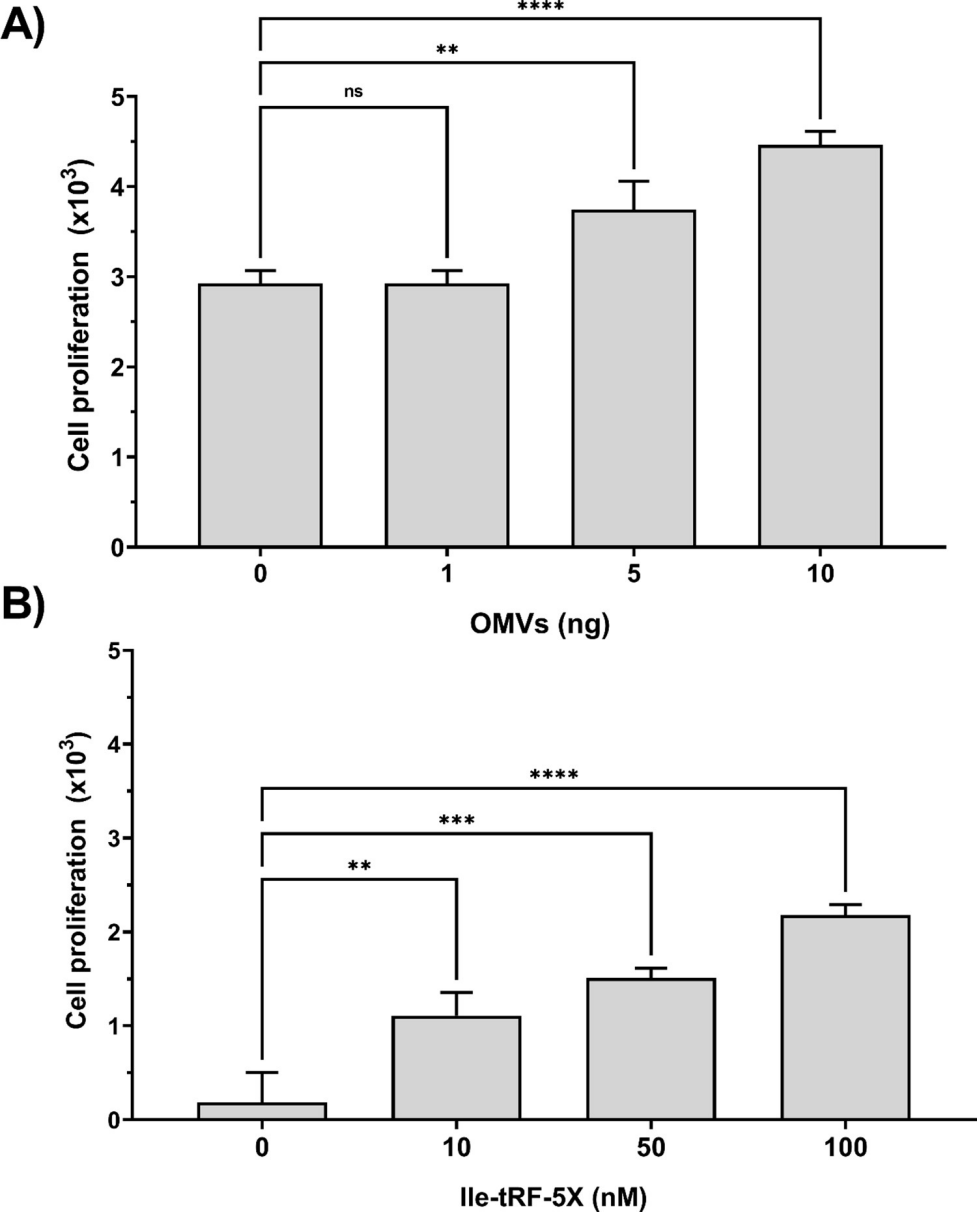
Bacterial OMVs and Ile-tRF-5X dose-dependently enhance human cell proliferation. (**A-B**) Human HCT116 cells were incubated with increasing amount of bacterial OMVs or transfected with increasing amount of Ile-tRF-5X. Cell proliferation was measured by plotting cell number from XTT-based absorbance (450 nm) measurements. Each dataset was normalized with its corresponding control (see materials and methods). **Statistical analysis**. Data were calculated from three biological replicate measurements (n = 3; mean ± SD), and each sample was tested in triplicate. One-way analysis of variance (ANOVA) and Holm-Šídák’s multiple comparisons test (post-hoc test) was used for statistical analysis. Statistically significant differences (fold change vs. control) are indicated as follows: ** p < 0.01; *** p < 0.001; **** p < 0.0001. ns, not significant.

The effect of bacterial OMVs and Ile-tRF-5X on proliferation of HCT116 cells was determined using a colorimetric assay. Cell proliferation was moderately increased upon exposure to bacterial OMVs (**Fig 11A**), but more substantially in response to increasing concentrations of Ile-tRF-5X (**Fig 11B**).

The bacterial OMV-induced cell proliferation was not affected by pre-treatment of the bacterial OMVs with an antisense to Ile-tRF-5X (**S9 Fig**).

To identify the human cell MAPK signaling pathway potentially regulated by bacterial OMVs and Ile-tRF-5X, we measured transcript levels of p38α MAPK, the transcription factor c-Jun and the cell division cycle 25 A (Cdc25A) phosphatase. Both bacterial OMVs and vsRNA Ile-tRF-5X promoted expression of Cdc25A and MAPK14 transcripts, whereas c-Jun mRNA seemed to be specifically modulated by OMVs, but not by Ile-tRF-5X (**S10 Fig**).

Finally, we performed a quantitative analysis of a dozen human cell transcripts relevant to inflammation and apoptosis to document their changes in expression levels in the presence of bacterial OMVs or Ile-tRF-5X (**S11 Fig**).

Some inflammatory cytokines, including interleukin-1 beta (IL-1β) and interleukin-6 (IL-6) were significantly upregulated by bacterial OMVs. In comparison, Ile-tRF-5X did not induce significant up or down regulation of the tested cytokines. The prototypical human inflammatory caspase-1 and the proapoptotic Bax were found to be upregulated in the presence of bacterial OMVs, whereas Ile-tRF-5X stimulated the survival pathway through upregulation of the apoptosis suppressor gene Bcl-2.

Taken together, these results suggest that bacterial OMVs and Ile-tRF-5X are potentially involved in the controlled regulation of human cell proliferation and death.

## Discussion

In this study, we have pursued our previous finding, in *E*. *coli* K-12 MG1655 and their OMVs, of an abundant 13-nt vsRNA [44], known as Ile-tRF-5X, and report on its potential role in bacteria-host interactions, and that of bacterial OMVs to act as a natural carrier. To simulate the intestinal microbiota environment, we chose the intestinal bacterium *E*. *coli*, which remains the most studied organism in modern biology [59], and the colonic epithelial HCT116 cell line as human host cells [60].

We observed that bacterial Ile-tRF-5X is differentially expressed in response to environmental factors. However, under normal laboratory conditions, either during the exponential or stationary phase, or upon inhibition of transcription and translation, the levels of Ile-tRF-5X in bacteria remained unchanged, although these processes are associated with a major shift in the abundance of tRNA [61, 62]. This uncoupling between tRF and tRNA levels in some conditions may seems intriguing but reinforces the idea that tRFs may have specialized roles different from their tRNA precursors. A lack of correlation has previously been reported between the accumulation of tRFs and different stresses applied to *Streptomyces coelicolor*, such as amino acid starvation, stringent response, or ribosome inhibition [63].

Stress conditions can lower the fraction of RNA polymerases that transcribe tRNA by about 60%, followed by degradation of the majority of bacterial tRNAs in less than twenty minutes [61, 64]. tRNA fragmentation is a highly conserved mechanism of the cellular response to stress in eukaryotes, as well [65]. Conversely, stress periods, including elevation of temperature [66], can also be a period of strong activation of the protein synthesis machinery [67]. The increase in bacterial Ile-tRF-5X levels might be explained not only by the controlled degradation of tRNAs (in this case, isoleucine) but also by their selective on-demand production by mechanisms that have yet to be fully understood. This duality in tRNA regulation, which may explain our results, at least in part, may be further supported by the demand-based model of tRNA degradation, developed by Sørensen and colleagues [68], suggesting that the tRNA pool is more dynamically regulated than previously thought. Moreover, previous findings have suggested that, in many respects, the metabolism of stable (tRNA and rRNA) and unstable (mRNA) RNAs are very similar [69] and, therefore, dynamically regulated to fit the bacteria’s needs.

The increase of Ile-tRF-5X during environmental stress situations (closer to what could be encountered in real conditions) suggests potential regulatory functions. In eukaryotes, these functions are now established and continue to expand [70]. Recent studies in bacteria have provided rational evidence to support these hypotheses (reviewed in [25]). Lalaouna et al. [23] reported the existence of an *E*. *coli* tRF derived from an external transcribed spacer (ETS) capable of modulating the activities of the sRNAs RyhB and RybB, which coordinate the bacterial responses to iron deprivation [71] and cell envelope homeostasis [72], respectively. Furthermore, they suggest functional conservation of these fragments in the ETS and ITS (internal transcribed spacer) of tRNAs from many species belonging to Enterobacteriaceae family and an important major role for RNAse E in their release [23].

By transiently inhibiting RNAse E or RNase P, we found that only RNAse E, which play a central role in stable-RNA processing including two-thirds of pre-tRNAs [73], may contribute to Ile-tRF-5X levels. Initiation of tRNA maturation by RNase E is essential for cell viability in *E*. *coli* [74], and Ile-tRF-5X is formed of the first 13 nucleotides of the mature tRNA-Ile. RNase E is also known to be involved in the glycolytic pathway of the bacteria [75] and hence we can speculate that its modulation may vary depending on the culture condition (LB vs. M63) more specifically on the parameter of carbon source availability in the M63 medium. The accumulation of tRF observed in M63 could therefore be, among other effects, the result of a decrease in RNase E efficiency due to its nutritional dependence. In fact, the role of RNase E in tRNA maturation is well known [49, 74, 76] but until now its potential contribution to tRF generation has never been documented and our study provides a new insight in this direction.

The lack of RNase P involvement may be explained by recent findings stipulating that the essential function of RNase P is not the 5’ maturation of pre-tRNAs, but rather the generation of pre-tRNAs from polycistronic operons [77].

Unlike tRNA genes that belong to tRNA operons [78], the tRNA-Ile gene is surrounded by rRNAs (*rrnA*, *rrnD*, and *rrnH*) operons, in a region with high evolutionary conservation which even serves as a tool for species characterization [79, 80]. More specifically, tRNA-Ile is part of the 16S-23S rRNA ITS [81]. It is worth recalling that tRNA and tRF processing may involve multiple independent pathways and multiple ribonucleases with overlapping functions [48]. This complexity made it challenging to identify the key enzymes mediating Ile-tRF-5X biosynthesis and/or turnover. The 16S and 23S rRNAs are respective components of the small and large ribosomal subunits that ultimately form the functional 70S ribosome [82]. The coexistence of tRNAs in ITS of rRNA operon remains enigmatic although widely reported [83]. It has been shown that all seven *E*. *coli* rRNA operons (including the 3 hosting the Ile-tRNA gene) are needed for optimal adaptation to changing physiological conditions [84]. Such localization of tRNA genes (and, therefore, tRFs) is probably not a coincidence, and may confer a strategic, functional advantage. Ideally, we would have needed a mutant strain of the isoleucine tRNA and another of the Ile-tRF-5X to better endorse the precise contribution at either the intracellular level or in OMVs. However, given the location of the genes, the removal of the three source sites would have led to adverse effects that would have biased data interpretation.

The selective enrichment of vsRNAs in OMVs and their effective transfer to human host cells were previously evidenced by other teams [34, 40, 85, 86]. The various OMV entry routes into host cells are discussed by O’Donoghue and Krachler [87], but the pathway(s) leading to their secretion from the parent bacterial cell is not well understood. Nevertheless, our study provides a different angle of perceiving these interactions in the largest symbiotic ecosystem in the gut microbiota [88], although it can hardly represent such a complex system and the biological events that take place.

Bacterial vsRNAs secreted in OMVs can potentially target a wide range of human host transcripts, which they may modulate to the advantage of the bacteria or, in a “commensal perspective”, control a win-win balance. This phenomenon implies the existence of an endless number of highly regulated exchange/pathways between bacteria and the host cells. We focused on host MAP3K4, whose family (MAPKs) had already been reported as potentially involved in the cascade of reactions triggered by bacteria and/or their tRFs [39, 40, 70, 89], besides having identified dozens of binding sites for Ile-tRF-5X in this mRNA transcript.

The quantification of Ile-tRF-5X, either post-transfection or post-incubation with bacterial OMVs, suggests that Ile-tRF-5X remains stable in human cells, which is probably conferred by its secondary hairpin structure, in addition to the contribution of their parental tRNA biotypes. This apparent stability might also be conferred by binding to effector protein complexes, as it is the case for some eukaryotic tRFs [90]. The Ile-tRF-5X transfection and OMV incubation approaches that we have used with human host cells have their own limitations, although comparable amounts of Ile-tRF-5X were used in this assay. Nevertheless, we noticed that the Ile-tRF-5X delivered by bacterial OMVs had a slightly higher rate of decrease than those directly transfected, suggesting a potential protective effect of OMVs or a slower internalization and/or intracellular release. However, the differences in the delivery for OMVs-associated and synthetic Ile-tRF-5X could affect these observations.

We observed that the MAP3K4 target candidate is upregulated at the mRNA level when human host cells are treated with bacterial OMVs, their sRNA content or Ile-tRF-5X alone. Bacterial RNA is a potent trigger for cell innate immune activation [91] and, in this respect, there is a high potential for MAPK pathways to be activated. This is in accordance with Tsatsaronis et al. [39], who proposed that the RNA content of extracellular vesicles may be sensed by endosomal receptors and, through a series of cascade reactions, lead to the activation of MAPKs.

Only bacterial OMVs induced the upregulation of MAP3K4 at the protein level. The decoupling of protein from mRNA levels may be explained, apart their measurement at a single time point, by the complexity of MAP3K4 transcript post-transcriptional modifications [92]. Being sensitive to a myriad of extracellular stimuli, MAPKs have developed a signal specificity related to the duration and magnitude of their interaction activities [93]. Here, OMVs appear to provide additional signal enhancement elements that vsRNAs do not have.

The binding of miRNAs involves the seed sequence, which is, on average, only 6–8 nt long, largely below the length of the vsRNA Ile-tRF-5X. From a molecular basis, our results suggest a model where Ile-tRF-5X and other vsRNAs compete with the pool of miRNAs regulating the 3’UTR of MAP3K4. By limiting the (down)regulatory effects of miRNAs, Ile-tRF-5X vsRNAs would contribute to the stabilization of MAP3K4 transcripts. The absence of Dicer and of most miRNAs, which are known to occupy binding sites in mRNA 3’UTRs, would be expected to facilitate access of Ile-tRF-5X to the 3’UTR of MAP3K4 mRNA. Under these conditions, Ile-tRF-5X does not seem to have intrinsic gene regulatory properties and may not be a simple on/off switch, but rather a modulator with sophisticated effects on phenotype [94].

This is what we have also reported in a previous study where we demonstrated that a 12-nt miRNA fragment (a “semi-miRNA”), without evidence of any involvement in a silencing complex, was able to modulate the activity of the miRNA from which it derived [95]. Similar to Ile-tRF-5X, semi-miRNA were probably too short to have direct gene regulatory properties [95]. It may not be excluded that miRNAs from non-canonical biogenesis pathways [96] could take over to interact differently with vsRNAs, such as Ile-tRF-5X. Of over fifteen miRNAs identified as potential MAP3K4 inhibitors, only miR-148a, a member of the miR-148/152 family, has been validated by three strong methodologies (reporter assay, qPCR, Western blot) [55, 97]. The seed sites of miR-148a do not overlap with those of Ile-tRF-5X but are 40 nt apart.

To study the global role of miRNAs, Dicer knockout models are often used. However, it is important to point out that Dicer is not only involved in the maturation of miRNAs and is probably implicated in several other regulatory processes [98], thus making the “Dicer phenotype” more challenging to interpret and hardly solely attributable to miRNA depletion.

It is difficult to assess the effect of vsRNAs on the entire MAP3K4 transcript structure and, more specifically, on its 3’UTR. However, beyond on-site competition, it is possible that their interactions may cumulatively or cooperatively lead to structural modifications that contribute to the post-transcriptional modulation of MAP3K4. Moreover, the possible cooperative effect of miRNAs [99] on the 3’UTRs may be perturbed by vsRNAs, resulting in an alleviation of the repression or, more drastically, a loss of miRNA function.

Bacterial Ile-tRF-5X has also predicted binding sites in the 5’UTR of MAP3K4 mRNA, a region where interactions can often lead to activation of transcription [100], which may contribute to increase MAP3K4 mRNA levels. Despite the normalization, which accounts for the variation in transfection efficiency and cell viability [101], the dual luciferase assay system made use of the MAP3K4 mRNA 3’UTR and, therefore, does not take into account the possible contribution of the ORF or 5’UTR, or the expression level of the endogenous target (here, MAP3K4). The list of potential factors involved in the modulation of MAP3K4 via vsRNAs is therefore non-exhaustive.

MAP3K4 is a ubiquitously expressed component of the stress-activated MAPK signaling module, which directly phosphorylates and activates the JNK and p38 families of MAPKs [102]. Depending on the context, MAP3K4 is either a proto-oncogene or a tumor suppressor. MAP3K4 stimulation may result in cell proliferation, a cellular response downstream of the signaling cascade regulated by MAP3Ks [58]. Although presenting several redundancies and crossovers, the two main MAPK signaling pathways (p38 and JNK) are differentially stimulated by bacterial OMVs and Ile-tRF-5X. OMVs seem to be able to engage indiscriminately both p38 and JNK pathways, while Ile-tRF-5X seems to be more specific to the p38 pathway (**S10 Fig**). Furthermore, p38 is required for cell proliferation and survival in colorectal cancer cells [103]. MAPKs constitute a dense network of pathways (crosstalk) that can hardly be partitioned from the functional point of view [104].

Several studies have clearly established these links between (endogenous) tRF or MAPKs expression and cell proliferation [19, 105, 106]. Host cell proliferation following incubation with OMVs has also been described in the literature [107, 108]. The colorimetric assay is also a mirror of viability and toxicity of metabolically active cells. Thus, fluctuation in proliferation may be related to mitochondrial abundance and cell viability (vs. death). Given the discrepancy observed in the induction of Bax (pro-death) and Bcl-2 (pro-survival) transcripts by OMVs, on one hand, and the vsRNA Ile-tRF-5X on the other hand, we can speculate on an in-balance bacterial modulation between cell proliferation and host cell death program. The specific induction of Caspase-1 by bacterial OMVs may trigger pyroptosis instead of apoptosis, as documented in previous studies [109]. It is necessary, however, to see at the protein level if all these patterns and phenotypes are confirmed and more precisely how they are affected in an in vivo experiment.

**Fig 12:**
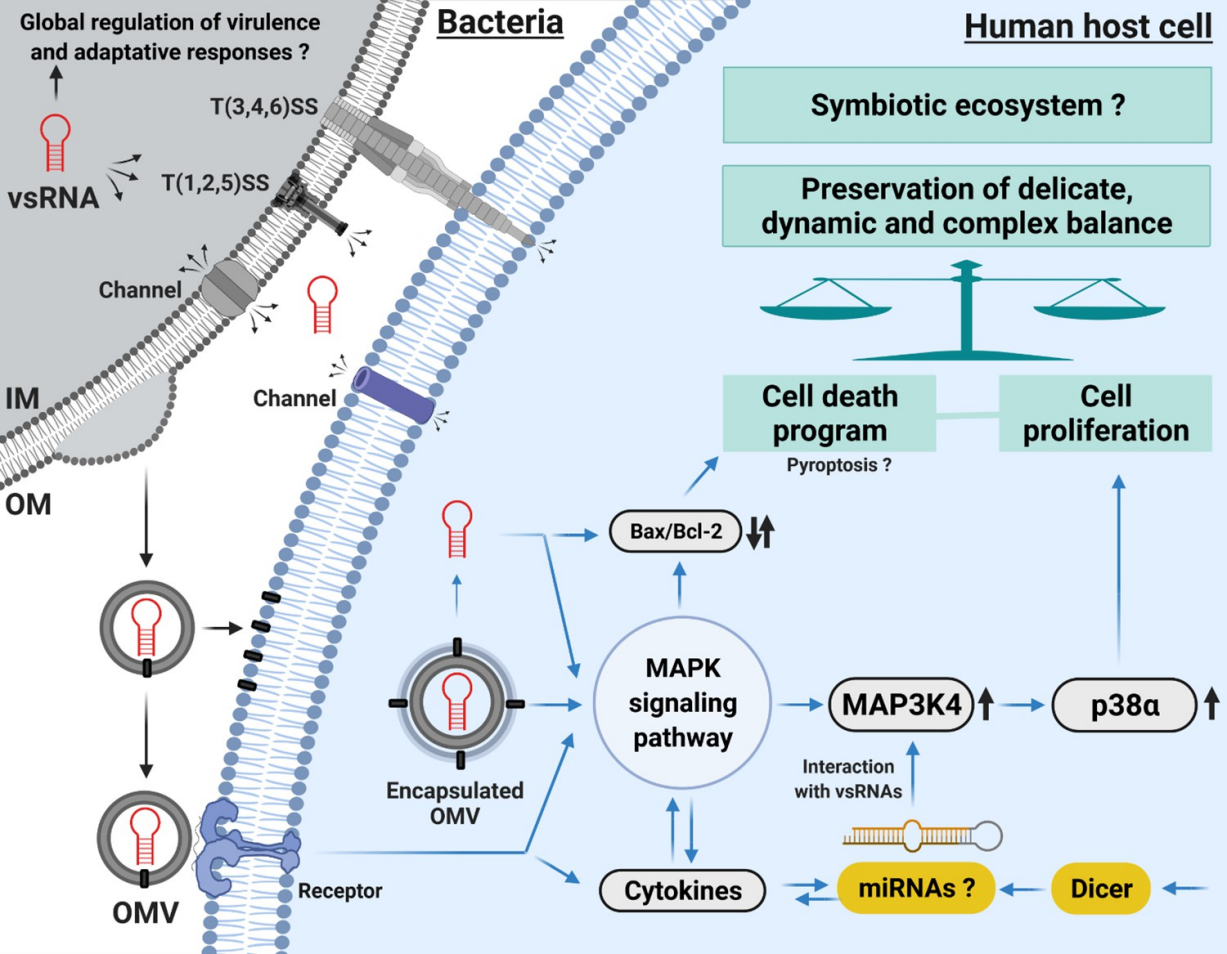
Schematic representation of how bacterial OMVs and their content in vsRNAs, such as Ile-tRF-5X, may be involved in preserving the delicate, dynamic and complex balance of homeostasis. IM = Inner Membrane; OM = Outer Membrane; OMV = Outer Membrane Vesicle; T(1,2,3,4,5,6)SS = Type (1,2,3,4,5,6) Secretion System. Original figure created with BioRender.com based on our previous review [27]. Not drawn to scale.

Finally, it has been shown that RNA fragments from plants, bacteria and fungi are ubiquitous in human plasma [110, 111] and may be delivered to any organ. Considering the present study in this broader perspective, bacterial OMVs and sRNAs, such as Ile-tRF-5X, may serve as universal mediators of microbial communication [39] and biomarkers of health status [111]. Also, on the host side, miRNAs were found to directly affect the growth of gut bacteria and were deemed essential for their maintenance [112]. The development of HTS technologies and the growing interest in bacterial OMVs and their RNA content being transferred to human host cells provide an unprecedented opportunity to rebuild our current understanding of host-bacteria relationships, which may either be pathogenic or symbiotic. Our study pleads in favor of studying vsRNAs, like the 13-nt Ile-tRF-5X, to provide new perspectives on the existing symbiosis between bacteria and their hosts. As an illustrative overview, we propose a schematic diagram representing how bacterial OMVs and their RNA content may be involved in the preservation of the delicate, dynamic and complex balance of homeostasis (**Fig 12**).

## Materials and methods

Critical materials and resources are summarize in Table 1.

**Table 1:** STAR★ Methods—Key Resources.

REAGENT or RESOURCE	SOURCE	IDENTIFIER
Bacterial Strains		
*Escherichia coli* MG1655 WT (EM1055)	Dr. Eric Massé	See S1 Table
*Escherichia coli* rne-3071 ts (EM1277)		
*Escherichia coli* rnpA ts (KP1036)		
Chemicals, Peptides, and Recombinant Proteins		
Polyethylenimine	Sigma	Cat# 919012
Lipofectamine 2000	Invitrogen	Cat# 11668019
Opti-MEM	Invitrogen	Cat# 31985062
PKH67 dye	Sigma	Cat# MINI67
HBSS 1X	Wisent	Cat# 311-513-CL
CellTracker Red CMTPX Dye	Invitrogen	Cat# C34552
SlowFade Gold Antifade Mountant	Invitrogen	Cat# S36942
TRIzol	Invitrogen	Cat# 15596026
RNAzol RT	Sigma	Cat# R4533
SSo Advanced SYBR Green mix	Biorad	Cat# 1725271
RIPA buffer	Cold Spring Harbor	CSH protocols
Complete EDTA-free Protease Inhibitor cocktails	Roche	Cat# 4693132001
PhosSTOP	Roche	Cat# 4906845001
anti-MAP3K4 (mouse monoclonal)	SCBT	Cat# sc-166197
anti-β-tubulin (mouse monoclonal)	SCBT	Cat# sc-5274
Critical Commercial Assays		
Dual-Luciferase Reporter Assay kit	Promega	Cat# E1980
Cell Proliferation Kit II	Roche	Cat# 11465015001
Exobacteria Kit	S. Biosciences	Cat# EXOBAC100A-1
VacuCap filters	Pall	Cat# 4634 / Cat# TA4632
RiboPure Bacteria Kit	Invitrogen	Cat# AM1925
HiFlex miScript II RT Kit	Qiagen	Cat# 218160
MicroAmp Fast Optical 96-Well Plate	Applied Biosystem	Cat# 4346907
miRCURY LNA RT Kit	Qiagen	Cat# 339340
miRCURY LNA SYBR Green PCR Kit	Qiagen	Cat# 339346
Multiplate PCR plate	Biorad	Cat# MLL9601
Clarity Max Western ECL Substrate	Biorad	Cat# 1705062
Deposited Data		
Raw and processed data from sRNA-Seq analysis	Ref [44]	BioProject accession: PRJNA826503 GEO accession: GSE200758 DOI: https://www.ncbi.nlm.nih.gov/geo/query/acc.cgi?acc=GSE200758
Experimental Models: Cell Lines		
HCT116 WT	ATCC	Cat# ATCC CCL-247
HCT116 Dicer -/-	This paper	NA
Oligonucleotides		
Primers	IDT	S2 Table
Recombinant DNA		
psiCHECK-II vector	Promega	Cat# 8021
psiCHECK-II + MAP3K4 3’UTR WT	This paper	S1 Information
psiCHECK-II + MAP3K4 3’UTR MUT	This paper	S1 Information
Software and Algorithms		
Volocity 4.2.1 software	Quorum Technologies	Technical resources/software/downloads/
Primer-BLAST	Ref [118]	Primer-BLAST website
StepOne Software	Thermofisher	Technical resources/software/downloads/
Image Studio Lite	Image Studio	Image studio lite website
ImageJ	Ref [125]	Technical resources/software/downloads/
GraphPad Prism 9.2.0	GraphPad	Technical resources/software/downloads/
Other		
Luminometer	TECAN	Cat# Infinite M1000 PRO
Sorvall WX+ Ultracentrifuge (T-1250 Fixed Angle Rotor)	Thermo Scientific	Cat#75000100
Beckman Coulter Optima Ultracentrifuge (TLA 100.4 rotor)	Beckman	Cat# TL 100
Wave FX-Borealis—Leica DMI 6000B	Quorum Technologies	NA
ImagEM camera	Hamamatsu	NA
NanoDrop 2000 Spectrophotometer	Thermo Scientific	Cat# ND-2000
StepOne Real-Time PCR System	Thermofisher	Cat# 4376357
CFX Connect Real-Time PCR Detection System	Biorad	Cat# 1855200
C-DiGit Blot Scanner	LI-COR Biosciences	Cat# 3600

### Bacterial strains and culture conditions

The bacterial strains used in this study and their experimental conditions are summarized in **S1 Table**. Unless otherwise stated, the reference strain of the study is *E*. *coli* MG1655. Bacteria were grown in Luria-Bertani (LB) medium or in M63 Glucose with 250 RPM aeration and on LB agar at 37°C.

### Human cell line culture

The human colorectal carcinoma cell lines HCT116 Wild type and Dicer-/- were grown in complete Dulbecco’s modified Eagle’s medium (DMEM, Wisent, St-Bruno, Canada, cat. no. 219-010-XK) supplemented with 10% fetal bovine serum, 1 mM L-glutamine, 100 units/ml penicillin, and 100 μg/ml streptomycin. Cells were grown and maintained in tissue culture plates and incubated at 37°C in a humidified atmosphere under 5% CO_2_. Cells were kept in the exponential growth phase and subcultured every 2–3 days.

### Plasmid constructs

The complete 3’UTR of MAP3K4 (4991–5506 nt, NCBI accession number NM_005922.4) was amplified by PCR and introduced downstream of the *Renilla luciferase* (Rluc) reporter gene in the XhoI/NotI cloning sites of the psiCHECK-II vector (Promega) to generate psiCHECK-II-3UTR-MAP3K4-WT.

A reporter construct bearing a mutated version of the 3’UTR of MAP3K4 was also engineered by using gBlocks gene fragments (Integrated DNA Technologies, Inc.) introduced in the XhoI/NotI cloning sites of psiCHECK-II vector to produce psiCHECK-II-3UTR-MAP3K4-MUT. The mutations (transversion substitution) were designed to abolish the interactions with the vsRNA Ile-tRF-5X and affect every other nucleotide of the binding sites. These constructions are described in **S1 Information.** All the constructs were confirmed by DNA sequencing at the Plateforme de Séquençage et Génotypage des Génomes (Centre de Recherche du CHU de Québec–CHUL, QC, Canada).

### Cell transfection and dual luciferase assay

Three hundred thousand cells were cultured in 6-well plates and transfected the following day at 70–80% confluency using polyethylenimine [113] (PEI; Sigma, ON, Canada, cat. no. 919012) or lipofectamine 2000 [114] (Invitrogen, ON, Canada, cat. no. 11668019) as described previously with slight modifications. The vsRNAs or plasmids to be transfected as well as the transfection reagents were diluted in Opti-MEM (Invitrogen, ON, Canada, cat. no. 31985062).

Forty-eight [48] h after transfection with psiCHECK-II vectors, cells were washed with PBS and lysed with 500 μl of passive lysis buffer. Luciferase activities were measured using the Dual-Luciferase Reporter Assay System (Promega, WI, USA, cat. no. E1980) on a luminometer (TECAN INFINITE M1000 PRO), according to the manufacturer’s instructions. Rluc expression was reported relative to the expression of the internal *Firefly luciferase* (Fluc) reporter control. Rluc expression was further normalized to the control in which cells were co-transfected with a synthetic mock vsRNA, referred to as mock control. All assays were conducted in triplicates in 96-well plates.

### Cell proliferation assay

For the quantification of cellular proliferation, we used the Cell Proliferation Kit II (Roche, Mannheim, Germany, cat. no. 11465015001), a colorimetric assay based on XTT (sodium 3´-[1- (phenylaminocarbonyl)- 3,4- tetrazolium]-bis (4-methoxy6-nitro) benzene sulfonic acid hydrate). A standard range of 0–30,000 cells were used to define the appropriate cell concentration and incubation time for the cell proliferation test (**S1 Fig**). Next, HCT116 cells were incubated in the presence of bacterial OMVs or transfected with vsRNAs at different concentrations in 96-well plates. After 48 h, the XTT labeling mixture was added to the microplate. After 3 h of incubation, the absorbance was measured using a microplate (ELISA) reader (spectrophotometer) at 450 nm wavelength.

### Isolation and characterization of OMVs

Outer membrane vesicles (OMVs) were purified from the reference strain *E*. *coli* K-12 MG1655, as described previously with some modifications [115, 116] or with the Exobacteria Kit (System Biosciences, CA, USA, cat. no. EXOBAC100A-1) following the manufacturer’s instructions.

Briefly, *E*. *coli* K-12 MG1655 was grown in 120 ml of LB to an OD_600_ of ~0.5, after which the supernatant was collected by centrifugation at 10,000 x g for 10 min at 4°C. The supernatant was filtered through a 0.45 and 0.2 μm-pore size VacuCap filters (PALL, MI, USA, cat. no. 4634, TA4632), after which an inoculum was taken to confirm the absence of bacteria on LB agar. Bacterial OMVs were then washed twice with PBS and pelleted each time by ultracentrifugation at ~200,000 x g for 2 h at 4°C in a Thermo Scientific Sorvall WX+ Ultracentrifuge with the T-1250 Fixed Angle Rotor. After removing the supernatant, OMVs were resuspended in 200 μl sterile PBS.

Isolation of OMVs by the Exobacteria Kit use a precipitation-free gravity column system. Based on the same approach, 30 ml of pre-cleared (centrifuged and filtered as above) bacterial culture media were used to harvest OMVs following the manufacturer’s instructions. Bacterial OMVs were collected in 1.5 ml of elution buffer.

Purified OMVs were subjected to quality control including agar plating to ensure lack of bacterial contamination, Coomassie blue stained gel, transmission electron micrograph (TEM) and dynamic light scattering (Zetasizer). These steps have been described in detail elsewhere [44]. Purified OMVs were stored at -80°C before being used for downstream applications.

### Confocal microscopy

Bacterial OMVs were stained with the green, fluorescent membrane dye PKH67 (Sigma, ON, Canada, cat. no. MINI67) at 1 μM final concentration in Diluent C (Sigma) for 5 min. The stained OMVs were then pelleted at 30,000g for 1 h in a TL-100 Optima Ultracentrifuge (Beckman) using rotor TLA 100.4. PKH67-labeled OMVs were washed in HBSS 1X (Wisent, QC, Canada, cat. no. 311-513-CL) to get rid of the free dye and then incubated with the fluorescently labeled HCT116 cells. HCT116 cells were labeled with the CellTracker Red CMTPX Dye (Invitrogen, ON, Canada, cat. no. C34552) for 30 min at 37°C in darkness and washed with PBS.

After incubation for the indicated times, trypsin was used for cell dissociation followed by centrifugation at room temperature for 5 min. Cells were then resuspended in 100 μl 2% PFA and kept for 15 min at room temperature to allow fixation. Cell deposition on slides was performed using cytospin. One drop of Gold Antifade Mountant with DAPI (SlowFade, ON, Canada, cat. no. S36942) was applied directly to the slide, which was then sealed with varnish and imaged using a Wave FX-Borealis—Leica DMI 6000B (Quorum Technologies) microscope (63X) with ImagEM camera (Hamamatsu, 512x512 pixels) at the CHUL–Université Laval Bio-imaging platform. The images were processed with the Volocity 4.2.1 software (Quorum Technologies).

### RNA isolation

Total RNA from bacterial strains was extracted using the hot phenol procedure [117] or the RiboPure Bacteria Kit (Invitrogen, ON, Canada, cat. no. AM1925) following the manufacturer’s recommendations. Total RNA from the human cell lines was extracted using TRIzol (Invitrogen, ON, Canada, cat. no. 15596026) or RNAzol RT (Sigma, MO, USA, cat. no. R4533) reagents following the manufacturer’s recommendations. Bacterial OMV RNA was isolated with RNAzol RT (Sigma, MO, USA, cat. no. R4533) reagents following the manufacturer’s recommendations (allow selective isolation, enrichment, of small RNAs <200 nt). All RNA samples were subjected to treatment with DNase I when applicable, quantified with the NanoDrop 2000 Spectrophotometer (Thermo Scientific, cat. no. ND-2000) and kept at -80°C for subsequent experiments.

### RT-qPCR

cDNA was generated by reverse transcription using the HiFlex miScript II RT Kit (Qiagen, MD, USA, cat. no. 218160) from 1 μg of DNase-treated RNA following the manufacturer’s protocol. After diluting the cDNA (1/10), qPCR was performed using the SSo Advanced SYBR Green mix (Bio-Rad, CA, USA, cat. no. 1725271) in 0.1 ml MicroAmp Fast Optical 96-Well Reaction Plate (Applied Biosystem, cat. no. 4346907).

The final concentration of the primers (Integrated DNA Technologies, Inc.) used in RT-qPCR was 500 nM and their sequence are listed in **S2 Table**. The primers were designed with Primer-BLAST tools and recommendations [118]. Primers were chosen to allow specific amplification of the target messengers (span exon-exon junction). Temperature gradient tests were performed to determine the best annealing temperature for each primer pair.

Unless otherwise specified, qPCR reactions were performed using the StepOne Real-Time PCR System (cat. no. 4376357). Unless otherwise specified, all data obtained (from StepOne Software) were normalized with reference genes (ACTB or GAPDH) and reported to the controls. The relative quantitation was calculated using the ΔΔCt method [119].

### vsRNA detection by splint-ligation-based strategy

The splint-ligation detection of vsRNA Ile-tRF-5X, which also relies on data from the literature on small RNA quantification [120, 121], was described previously [122] and is illustrated in **S12 Fig**. For the highest efficiency and specificity, several adjustments were carried out at the pre-annealing (splint+adapter+vsRNA), ligation, reverse transcription, and qPCR steps. We employed a ligation step (T4 RNA ligase) to elongate vsRNA Ile-tRF-5X prior to RT-qPCR. A 13-nt adapter was tagged to the sequence of interest (Ile-tRF-5X) with the use of a specific 20-nt splint.

The adapter-ligated vsRNA and total RNA content were converted to cDNA with the miRCURY LNA RT Kit (Qiagen, MD, USA, cat. no. ID 339340). Specific and sensitive custom miRNA primer set designed by the Exiqon experts and optimized with LNA technology [123] were used.

After diluting the cDNA (1/10), qPCR was performed using the miRCURY LNA SYBR Green PCR Kit (Qiagen, MD, USA, cat. no. 339346) with CFX Connect Real-Time PCR Detection System (Bio-Rad, cat. no. 1855200) in 96-well plates (Multiplate PCR plate, cat. no. MLL9601) following the manufacturer’s protocol. The UniSp6 RNA spike-in was used for cDNA synthesis and PCR amplification normalization. In bacteria, 23S and/or 16S served as reference genes.

### Protein extraction

Unless otherwise specified and in order to obtain RNA and protein from the same sample, proteins were extracted directly from the organic part remaining after RNA extraction with TRIzol reagent (Invitrogen, cat. no. 15596026). A sonication step was included to ensure better protein dissolution (5 cycles of 15 sec sonication and 30 sec ice incubation; sonicator settings: amplitude 80%, pulses 90%).

Homemade radioimmunoprecipitation assay (RIPA) buffer [124] was also used to extract OMV-associated proteins. One tablet of each of the complete EDTA-free Protease Inhibitor cocktails (Roche, QC, Canada, cat. no. 4693132001) and PhosSTOP (Roche, QC, Canada, cat. no. 4906845001) was added per 10 ml 1X RIPA buffer.

### Western blot

Protein extracts were analyzed by 10% (wt/vol) SDS-PAGE and immunoblotting using anti-MAP3K4 (Mitogen-activated protein kinase kinase kinase 4, SCBT, TX, USA, cat. no. sc-166197), anti-β-tubulin (SCBT, TX, USA, cat. no. sc-5274) antibodies. Chemiluminescence detection was performed using C-DiGit Blot Scanner (LI-COR Biosciences) with Clarity Max Western ECL Substrate (Bio-Rad, CA, USA, cat. no. 1705062). The images were acquired with Software Image Studio Lite (Image Studio) and densitometry analyses were performed with ImageJ [125].

### Statistical analysis

The statistical method used is mentioned in each figure legend. All statistical analyses were performed using GraphPad Prism version 9.2.0 (GraphPad Software, Inc., La Jolla, CA, USA), with statistical significance set at p < 0.05. Details of the tests are mentioned under each figure when applicable.

### Contact for reagent and resource sharing

Further information and requests for resources and reagents should be directed to and will be fulfilled by the Lead Contact, Patrick Provost (patrick.provost@crchudequebec.ulaval.ca).

### Materials availability

This study did not generate new unique reagents.

## Supporting information

S1 Fig Setting up of the XTT test of cell proliferation. Determination of the optimal measurement time and the appropriate amount of cells.
(DOCX)

S2 Fig Bacterial Ile-tRF-5X and mature Ile-tRNA levels under different experimental conditions. (**A**) *E*. *coli* MG1655 bacteria were grown at 37°C up to the exponential (Reference, R) and stationary phases of growth in either complete (rich) LB or minimal M63 medium. (**B**) *E*. *coli* MG1655 bacteria were grown in LB medium at exponential phase (R) in different temperatures: 30, 37 and or 44°C. (**C**) Bacterial mRNA or protein synthesis was inhibited by addition of chloramphenicol or rifampicin, respectively, to cultures of *E*. *coli* MG1655 grown at exponential phase (R). The level of bacterial Ile-tRF-5X level is not modulated by transcription or translation activity (**D**) *E*. *coli* strains carrying heat-sensitive (hs) mutations in the essential genes rne-3071-hs (EM1277) and rnpA-hs (KP1036) were grown in LB medium at 30°C and then heat-shocked (44°C) to transiently inhibit ribonuclease (RNase) P or RNase E, which are involved in tRNA maturation. Bacteria RNase E contributes to Ile-tRF-5X biogenesis. For more details, see **Supplementary S1 Table**. In all conditions, the level of Ile-tRF-5X and Ile-tRNA were measured by LNA RT-qPCR. A spike-in (UniSp6) and reference genes (23S and/or 16S) were used as control and for normalization. The results are reported in fold change compared to the reference condition. **Statistical analysis**. Data were calculated from three biological replicate measurements (n = 3; mean ± SD). Two-way analysis of variance (ANOVA) and Dunnett’s multiple comparisons (fold change vs. reference test) or Šídák’s multiple comparisons test (Ile-tRF-5X vs. Ile-tRNA) were used for statistical analysis. Statistically significant differences (fold change vs. reference or Ile-tRF-5X vs. Ile-tRNA shown in purple) are indicated by stars (*), * p < 0.05; ** p < 0.01; *** p < 0.001; **** p < 0.0001; ns, not significant.
(DOCX)

S3 Fig Ile-tRF-5X level in different E. coli samples. Ile-tRF-5X was quantified from normalized RNA-Seq data in *E*. *coli* exponential growth phase (reference, ref, R), stationary phase, and after treatment with chloramphenicol (R+cat), rifampicin (R+rif) or heat shock to transiently inactivate RNase E (RNase E -). Fig 2A (LB medium), Fig 3 and Fig 4 (RNase E) are the quantitative validation of these data. One biological replicate. See [reference [1]] for more details.
(DOCX)

S4 Fig Proportion in copy number of intracellular Ile-tRF-5X or OMV-packaged Ile-tRF-5X. (DOCX)

S5 Fig Confocal microscopy imaging of labelled OMVs taken up by human HCT116 cells. HCT116 cells were stained with a Cell Tracker CMTPX™ (red) and then incubated for 2 h with bacterial OMVs labelled with PKH67 (green). The nuclei are stained with DAPI (blue). The XZ and YZ projection of Volocity shows that OMVs were internalized by HCT116 cells and localized mainly in the cytoplasm (See panel A at 2 h in Fig 5.).
(DOCX)

S6 Fig Human MAP3K4 mRNA harbors potential binding sites for bacterial Ile-tRF-5X. Three bioinformatics tools (microRT, blastN and RNAhybrid) used in combination predict several Ile-tRF-5X (5X) binding sites in the human MAP3K4 mRNA. Base pairing of the top three Ile-tRF-5X binding sites is shown on the left. The number of nucleotides (mer) involved in the interaction (seed sequences), their minimal free energy (MFE, according to RNAhybrid) and their position (5’UTR, ORF or 3’UTR) are listed in the table. * indicates that the 13th nt of Ile-tRF-5X is a C, thus allowing perfect base pairing with MAP3K4 mRNA in its ORF.
(DOCX)

S7 Fig Schematic representation of the predicted Ile-tRF-5X and miRNA base pairing in the MAP3K4 mRNA 3’UTR. The base pairing interactions between bacterial Ile-tRF-5X and human MAP3K4 mRNA (RNAhybrid) are shown in red, with the minimum free energy (Mfe) and position (nucleotides). The base pairing interactions between human miRNAs and MAP3K4 mRNA (Targetscan) are highlighted in grey. The nucleotides involved in the Watson–Crick base pairing are in bold. The numbers in square brackets ([]) correspond to the number of non-displayed nucleotides in the 3’UTR of MAP3K4 mRNA. The full MAP3K4 mRNA 3′ UTR sequence was cloned downstream of the humanized Rluc (hRluc) gene, in the dual-luciferase reporter gene expression vector psiCHECK-II, with hFluc as a normalization control. hRluc, humanized *Renilla luciferase* gene; hluc+, humanized *Firefly luciferase* gene.
(DOCX)

S8 Fig Secondary structure of 3’UTR MAP3K4 displaying the binding sites of miRNAs and Ile-tRF-5X. FORNA Web-based tools were used to illustrate RNA secondary structure of the full 3’UTR of MAP3K4. In blue we have the binding site positions of miRNAs and in red those of Ile-tRF-5X. For each of these actors, the positions (P) of the nucleotides (nt) are indicated.
(DOCX)

S9 Fig OMVs enhance HCT116 cell proliferation. Cell number was estimated from XTT-based absorbance (450 nm) measurement. The % of cell proliferation was deduced from that of the mock control set at 100%. The use of Ile-tRF-5X (as5X, 100nM) did not significantly reduce cell proliferation. Each data set is normalized with its corresponding control. **Statistical analysis**. All data presented were calculated from three biological replicate (n = 3) measurements ± SD and each sample was tested with 3 replicates. The one-way analysis of variance (ANOVA) and Holm-Šídák’s multiple comparisons test were used for statistical analysis. Statistically significant differences (fold change vs. mock) are indicated as follows: * p < 0.05; *** p < 0.001; **** p < 0.0001.
(DOCX)

S10 Fig Relative quantification of MAPK14 (p38α), CDC25A and c-JUN expressions at mRNA level in HCT116 cells by RT-qPCR after incubation with OMVs or transfection with Ile-tRF-5X. Data were normalized with a reference gene (ACTB), reported to mock control, and expressed with a relative quantitation method (ΔΔCt). **Statistical analysis**. All data presented were calculated from three biological replicate (n = 3) measurements ± SD. The one-way analysis of variance (ANOVA) and Dunnett’s multiple comparisons were used for statistical analysis. Statistically significant differences (fold change vs. mock) are indicated by stars (*), * p < 0.05; ** p < 0.01; *** p < 0.001, **** p < 0.0001.
(DOCX)

S11 Fig Changes in expression of cytokines and apoptosis factors in HCT116 cells after incubation with bacterial OMVs or transfection with Ile-tRF-5X. Relative mRNA expression was quantified by RT-qPCR. Data were normalized with a reference gene (ACTB), reported as fold change vs mock control, and expressed with the relative quantitation method (ΔΔCt). **Statistical analysis**. Data were calculated from three biological replicate measurements (n = 3; mean ± SD), and each sample was tested in triplicate. Two-way analysis of variance (ANOVA) and Holm-Šídák’s multiple comparisons test (post-hoc test) were used for statistical analysis. Statistically significant differences (fold change vs mock) are indicated as follows: * p < 0.05.
(DOCX)

S12 Fig vsRNA monitoring (Splint-ligation-based strategy). A) Experimental scheme for the detection of vsRNAs, here Ile-tRF-5X as example (See mat. & meth. section). B) specificity test of Exiqon Ile-tRF-5X primers. The primers can discriminate Ile-tRF-5X from any other sequence by up to one nucleotide difference using LNA technology. C) Single peak melt curve assessing the specificity of amplification. Ile-tRF-5X melt curve analysis shows the production of specific and single product. D) comparative analysis of Ile-tRF-5X quantifications by RNA-seq (semi-quantitative) and by qPCR reported in percentage. Ile-tRF-5X was quantified in *E*. *coli* exponential growth phase (reference = R), stationary phase, after treatment with rifampicin (R+rif) or chloramphenicol (R+cat), or finally in derived OMVs.
(DOCX)

S1 Table List of bacteria used in the study and their growth conditions. (DOCX)

S2 Table Primers used in the study. (DOCX)

S1 Information Details of the design of the plasmid construction for the dual luciferase assay with MAP3K4 3’UTR WT (A) and MAP3K4 3’UTR MUT (B). (DOCX)

## References

[1] Wagner EGH, Romby P. Small RNAs in bacteria and archaea: who they are, what they do, and how they do it. Adv Genet. 2015;90:133–208. doi: 10.1016/bs.adgen.2015.05.001 26296935

[2] Storz G, Vogel J, Wassarman KM. Regulation by small RNAs in bacteria: expanding frontiers. Mol Cell. 16 sept 2011;43(6):880–91. doi: 10.1016/j.molcel.2011.08.022 21925377 PMC3176440

[3] Cech TR, Steitz JA. The noncoding RNA revolution-trashing old rules to forge new ones. Cell. 27 mars 2014;157(1):77–94. doi: 10.1016/j.cell.2014.03.008 24679528

[4] Carrier MC, Lalaouna D, Massé E. Broadening the Definition of Bacterial Small RNAs: Characteristics and Mechanisms of Action. Annu Rev Microbiol. 8 sept 2018;72:141–61. doi: 10.1146/annurev-micro-090817-062607 30200848

[5] Simons RW, Kleckner N. Translational control of IS10 transposition. Cell. 1 sept 1983;34(2):683–91. doi: 10.1016/0092-8674(83)90401-4 6311438

[6] Mizuno T, Chou MY, Inouye M. A unique mechanism regulating gene expression: translational inhibition by a complementary RNA transcript (micRNA). Proc Natl Acad Sci USA. avr 1984;81(7):1966–70. doi: 10.1073/pnas.81.7.1966 6201848 PMC345417

[7] Morita T, Aiba H. RNase E action at a distance: degradation of target mRNAs mediated by an Hfq-binding small RNA in bacteria. Genes Dev. 15 févr 2011;25(4):294–8. doi: 10.1101/gad.2030311 21325130 PMC3042153

[8] Mohanty BK, Kushner SR. Regulation of mRNA Decay in Bacteria. Annual Review of Microbiology. 2016;70(1):25–44. doi: 10.1146/annurev-micro-091014-104515 27297126

[9] Lalaouna D, Simoneau-Roy M, Lafontaine D, Massé E. Regulatory RNAs and target mRNA decay in prokaryotes. Biochim Biophys Acta. juill 2013;1829(6–7):742–7. doi: 10.1016/j.bbagrm.2013.02.013 23500183

[10] Fröhlich KS, Vogel J. Activation of gene expression by small RNA. Current Opinion in Microbiology. 1 déc 2009;12(6):674–82. doi: 10.1016/j.mib.2009.09.009 19880344

[11] Papenfort K, Vanderpool CK. Target activation by regulatory RNAs in bacteria. FEMS Microbiology Reviews. 1 mai 2015;39(3):362–78. doi: 10.1093/femsre/fuv016 25934124 PMC4542691

[12] Argaman L, Hershberg R, Vogel J, Bejerano G, Wagner EGH, Margalit H, et al. Novel small RNA-encoding genes in the intergenic regions of Escherichia coli. Current Biology. 26 juin 2001;11(12):941–50. doi: 10.1016/s0960-9822(01)00270-6 11448770

[13] Chao Y, Papenfort K, Reinhardt R, Sharma CM, Vogel J. An atlas of Hfq-bound transcripts reveals 3′ UTRs as a genomic reservoir of regulatory small RNAs. The EMBO Journal. 17 oct 2012;31(20):4005–19. doi: 10.1038/emboj.2012.229 22922465 PMC3474919

[14] Chao Y, Vogel J. A 3′ UTR-Derived Small RNA Provides the Regulatory Noncoding Arm of the Inner Membrane Stress Response. Molecular Cell. 4 févr 2016;61(3):352–63. doi: 10.1016/j.molcel.2015.12.023 26805574

[15] Ren GX, Guo XP, Sun YC. Regulatory 3′ Untranslated Regions of Bacterial mRNAs. Frontiers in Microbiology. 2017;8:1276. doi: 10.3389/fmicb.2017.01276 28740488 PMC5502269

[16] Jose BR, Gardner PP, Barquist L. Transcriptional noise and exaptation as sources for bacterial sRNAs. Biochem Soc Trans. 30 avr 2019;47(2):527–39. doi: 10.1042/BST20180171 30837318

[17] Morozova O, Marra MA. Applications of next-generation sequencing technologies in functional genomics. Genomics. nov 2008;92(5):255–64. doi: 10.1016/j.ygeno.2008.07.001 18703132

[18] Jørgensen MG, Pettersen JS, Kallipolitis BH. sRNA-mediated control in bacteria: An increasing diversity of regulatory mechanisms. Biochimica et Biophysica Acta (BBA)—Gene Regulatory Mechanisms. 1 mai 2020;1863(5):194504. doi: 10.1016/j.bbagrm.2020.194504 32061884

[19] Lee YS, Shibata Y, Malhotra A, Dutta A. A novel class of small RNAs: tRNA-derived RNA fragments (tRFs). Genes Dev. 15 nov 2009;23(22):2639–49. doi: 10.1101/gad.1837609 19933153 PMC2779758

[20] Fu Y, Lee I, Lee YS, Bao X. Small Non-coding Transfer RNA-Derived RNA Fragments (tRFs): Their Biogenesis, Function and Implication in Human Diseases. Genomics Inform. déc 2015;13(4):94–101. doi: 10.5808/GI.2015.13.4.94 26865839 PMC4742329

[21] Cherlin T, Magee R, Jing Y, Pliatsika V, Loher P, Rigoutsos I. Ribosomal RNA fragmentation into short RNAs (rRFs) is modulated in a sex- and population of origin-specific manner. BMC Biology. 13 avr 2020;18(1):38. doi: 10.1186/s12915-020-0763-0 32279660 PMC7153239

[22] Lambert M, Benmoussa A, Provost P. Small Non-Coding RNAs Derived From Eukaryotic Ribosomal RNA. Noncoding RNA. 4 févr 2019;5(1). doi: 10.3390/ncrna5010016 30720712 PMC6468398

[23] Lalaouna D, Carrier MC, Semsey S, Brouard JS, Wang J, Wade JT, et al. A 3′ External Transcribed Spacer in a tRNA Transcript Acts as a Sponge for Small RNAs to Prevent Transcriptional Noise. Molecular Cell. 7 mai 2015;58(3):393–405. doi: 10.1016/j.molcel.2015.03.013 25891076

[24] Kumar P, Kuscu C, Dutta A. Biogenesis and Function of Transfer RNA-Related Fragments (tRFs). Trends Biochem Sci. 2016;41(8):679–89. doi: 10.1016/j.tibs.2016.05.004 27263052 PMC5173347

[25] Li Z, Stanton BA. Transfer RNA-Derived Fragments, the Underappreciated Regulatory Small RNAs in Microbial Pathogenesis. Frontiers in Microbiology. 2021;12:1252. doi: 10.3389/fmicb.2021.687632 34079534 PMC8166272

[26] Schwechheimer C, Kuehn MJ. Outer-membrane vesicles from Gram-negative bacteria: biogenesis and functions. Nat Rev Microbiol. oct 2015;13(10):605–19. doi: 10.1038/nrmicro3525 26373371 PMC5308417

[27] Diallo I, Provost P. RNA-Sequencing Analyses of Small Bacterial RNAs and their Emergence as Virulence Factors in Host-Pathogen Interactions. International Journal of Molecular Sciences. janv 2020;21(5):1627. doi: 10.3390/ijms21051627 32120885 PMC7084465

[28] Sender R, Fuchs S, Milo R. Revised Estimates for the Number of Human and Bacteria Cells in the Body. PLOS Biology. 19 août 2016;14(8):e1002533. doi: 10.1371/journal.pbio.1002533 27541692 PMC4991899

[29] Lee YK, Mazmanian SK. Has the microbiota played a critical role in the evolution of the adaptive immune system? Science. 24 déc 2010;330(6012):1768–73. doi: 10.1126/science.1195568 21205662 PMC3159383

[30] Vaca DJ, Thibau A, Schütz M, Kraiczy P, Happonen L, Malmström J, et al. Interaction with the host: the role of fibronectin and extracellular matrix proteins in the adhesion of Gram-negative bacteria. Med Microbiol Immunol. 2020;209(3):277–99. doi: 10.1007/s00430-019-00644-3 31784893 PMC7248048

[31] Toyofuku M, Nomura N, Eberl L. Types and origins of bacterial membrane vesicles. Nat Rev Microbiol. janv 2019;17(1):13–24. doi: 10.1038/s41579-018-0112-2 30397270

[32] Guerrero-Mandujano A, Hernández-Cortez C, Ibarra JA, Castro-Escarpulli G. The outer membrane vesicles: Secretion system type zero. Traffic. 2017;18(7):425–32. doi: 10.1111/tra.12488 28421662

[33] Dorward DW, Garon CF, Judd RC. Export and intercellular transfer of DNA via membrane blebs of Neisseria gonorrhoeae. J Bacteriol. mai 1989;171(5):2499–505. doi: 10.1128/jb.171.5.2499-2505.1989 2496108 PMC209926

[34] Sjöström AE, Sandblad L, Uhlin BE, Wai SN. Membrane vesicle-mediated release of bacterial RNA. Sci Rep. 20 oct 2015;5:15329. doi: 10.1038/srep15329 26483327 PMC4612299

[35] Ho MH, Chen CH, Goodwin JS, Wang BY, Xie H. Functional Advantages of Porphyromonas gingivalis Vesicles. PLoS ONE. 2015;10(4):e0123448. doi: 10.1371/journal.pone.0123448 25897780 PMC4405273

[36] Ghosal A, Upadhyaya BB, Fritz JV, Heintz-Buschart A, Desai MS, Yusuf D, et al. The extracellular RNA complement of Escherichia coli. Microbiologyopen. avr 2015;4(2):252–66. doi: 10.1002/mbo3.235 25611733 PMC4398507

[37] Valadi H, Ekström K, Bossios A, Sjöstrand M, Lee JJ, Lötvall JO. Exosome-mediated transfer of mRNAs and microRNAs is a novel mechanism of genetic exchange between cells. Nat Cell Biol. juin 2007;9(6):654–9. doi: 10.1038/ncb1596 17486113

[38] Yáñez-Mó M, Siljander PRM, Andreu Z, Zavec AB, Borràs FE, Buzas EI, et al. Biological properties of extracellular vesicles and their physiological functions. J Extracell Vesicles. 2015;4:27066. doi: 10.3402/jev.v4.27066 25979354 PMC4433489

[39] Tsatsaronis JA, Franch-Arroyo S, Resch U, Charpentier E. Extracellular Vesicle RNA: A Universal Mediator of Microbial Communication? Trends Microbiol. mai 2018;26(5):401–10. doi: 10.1016/j.tim.2018.02.009 29548832

[40] Koeppen K, Hampton TH, Jarek M, Scharfe M, Gerber SA, Mielcarz DW, et al. A Novel Mechanism of Host-Pathogen Interaction through sRNA in Bacterial Outer Membrane Vesicles. Whiteley, éditeur. PLOS Pathogens. 13 juin 2016;12(6):e1005672. doi: 10.1371/journal.ppat.1005672 27295279 PMC4905634

[41] Sahr T, Escoll P, Rusniok C, Bui S, Pehau-Arnaudet G, Lavieu G, et al. Translocated Legionella pneumophila small RNAs mimic eukaryotic microRNAs targeting the host immune response. Nat Commun. 9 févr 2022;13(1):762. doi: 10.1038/s41467-022-28454-x 35140216 PMC8828724

[42] Choi JW, Kim SC, Hong SH, Lee HJ. Secretable Small RNAs via Outer Membrane Vesicles in Periodontal Pathogens. J Dent Res. avr 2017;96(4):458–66. doi: 10.1177/0022034516685071 28068479

[43] Lambert M, Benmoussa A, Diallo I, Ouellet-Boutin K, Dorval V, Majeau N, et al. Identification of Abundant and Functional dodecaRNAs (doRNAs) Derived from Ribosomal RNA. International Journal of Molecular Sciences. janv 2021;22(18):9757. doi: 10.3390/ijms22189757 34575920 PMC8467515

[44] Diallo I, Ho J, Lalaouna D, Massé E, Provost P. RNA Sequencing Unveils Very Small RNAs With Potential Regulatory Functions in Bacteria. Frontiers in Molecular Biosciences [Internet]. 2022 [cité 3 juin 2022];9. Disponible sur: https://www.frontiersin.org/article/10.3389/fmolb.2022.914991 35720117 10.3389/fmolb.2022.914991 PMC9203972

[45] Kumar P, Anaya J, Mudunuri SB, Dutta A. Meta-analysis of tRNA derived RNA fragments reveals that they are evolutionarily conserved and associate with AGO proteins to recognize specific RNA targets. BMC Biology. déc 2014;12(1):78. doi: 10.1186/s12915-014-0078-0 25270025 PMC4203973

[46] Hartmann G, Honikel KO, Knüsel F, Nüesch J. The specific inhibition of the DNA-directed RNA synthesis by rifamycin. Biochim Biophys Acta. 1967;145(3):843–4. doi: 10.1016/0005-2787(67)90147-5 4863911

[47] Vázquez D. Inhibitors of protein biosynthesis. Mol Biol Biochem Biophys. 1979;30:i-x, 1–312. doi: 10.1007/978-3-642-81309-2 370549

[48] Mohanty BK, Kushner SR. Enzymes Involved in Post-transcriptional RNA Metabolism in Gram-negative bacteria. Microbiol Spectr. avr 2018;6(2): doi: 10.1128/microbiolspec.RWR-0011-2017 29676246 PMC5912700

[49] Li Z, Deutscher MP. RNase E plays an essential role in the maturation of Escherichia coli tRNA precursors. RNA. janv 2002;8(1):97–109. doi: 10.1017/s1355838202014929 11871663 PMC1370232

[50] Willkomm DK, Hartmann RK. An important piece of the RNase P jigsaw solved. Trends Biochem Sci. juin 2007;32(6):247–50. doi: 10.1016/j.tibs.2007.04.005 17485211

[51] Chang L, Karin M. Mammalian MAP kinase signalling cascades. Nature. mars 2001;410(6824):37–40. doi: 10.1038/35065000 11242034

[52] Venkatesh T, Suresh PS, Tsutsumi R. tRFs: miRNAs in disguise. Gene. 1 avr 2016;579(2):133–8. doi: 10.1016/j.gene.2015.12.058 26743126

[53] Kim YK, Kim B, Kim VN. Re-evaluation of the roles of DROSHA, Exportin 5, and DICER in microRNA biogenesis. Proc Natl Acad Sci U S A. 29 mars 2016;113(13):E1881–9. doi: 10.1073/pnas.1602532113 26976605 PMC4822641

[54] Boissonneault V, Plante I, Rivest S, Provost P. MicroRNA-298 and microRNA-328 regulate expression of mouse beta-amyloid precursor protein-converting enzyme 1. J Biol Chem. 23 janv 2009;284(4):1971–81. doi: 10.1074/jbc.M807530200 18986979 PMC2908704

[55] Agarwal V, Bell GW, Nam JW, Bartel DP. Predicting effective microRNA target sites in mammalian mRNAs. Elife. 12 août 2015;4. doi: 10.7554/eLife.05005 26267216 PMC4532895

[56] Krüger J, Rehmsmeier M. RNAhybrid: microRNA target prediction easy, fast and flexible. Nucleic Acids Research. 1 juill 2006;34(suppl_2):W451–4. doi: 10.1093/nar/gkl243 16845047 PMC1538877

[57] Kerpedjiev P, Hammer S, Hofacker IL. Forna (force-directed RNA): Simple and effective online RNA secondary structure diagrams. Bioinformatics. 15 oct 2015;31(20):3377–9. doi: 10.1093/bioinformatics/btv372 26099263 PMC4595900

[58] Dhillon AS, Hagan S, Rath O, Kolch W. MAP kinase signalling pathways in cancer. Oncogene. mai 2007;26(22):3279–90. doi: 10.1038/sj.onc.1210421 17496922

[59] Hayashi K, Morooka N, Yamamoto Y, Fujita K, Isono K, Choi S, et al. Highly accurate genome sequences of Escherichia coli K-12 strains MG1655 and W3110. Mol Syst Biol. 21 févr 2006;2:2006.0007. doi: 10.1038/msb4100049 16738553 PMC1681481

[60] Barrila J, Crabbé A, Yang J, Franco K, Nydam SD, Forsyth RJ, et al. Modeling Host-Pathogen Interactions in the Context of the Microenvironment: Three-Dimensional Cell Culture Comes of Age. Infection and Immunity. 86(11):e00282–18. doi: 10.1128/IAI.00282-18 30181350 PMC6204695

[61] Svenningsen SL, Kongstad M, Stenum TS, Muñoz-Gómez AJ, Sørensen MA. Transfer RNA is highly unstable during early amino acid starvation in Escherichia coli. Nucleic Acids Res. 25 janv 2017;45(2):793–804. doi: 10.1093/nar/gkw1169 27903898 PMC5314770

[62] Zundel MA, Basturea GN, Deutscher MP. Initiation of ribosome degradation during starvation in Escherichia coli. RNA. mai 2009;15(5):977–83. doi: 10.1261/rna.1381309 19324965 PMC2673067

[63] Haiser HJ, Karginov FV, Hannon GJ, Elliot MA. Developmentally regulated cleavage of tRNAs in the bacterium Streptomyces coelicolor. Nucleic Acids Res. févr 2008;36(3):732–41. doi: 10.1093/nar/gkm1096 18084030 PMC2241913

[64] Masaki H, Ogawa T. The modes of action of colicins E5 and D, and related cytotoxic tRNases. Biochimie. juin 2002;84(5–6):433–8. 10.1016/s0300-9084(02)01425-6 12423786

[65] Oberbauer V, Schaefer MR. tRNA-Derived Small RNAs: Biogenesis, Modification, Function and Potential Impact on Human Disease Development. Genes (Basel). 5 déc 2018;9(12):607. doi: 10.3390/genes9120607 30563140 PMC6315542

[66] Baer MF, Wesolowski D, Altman S. Characterization in vitro of the defect in a temperature-sensitive mutant of the protein subunit of RNase P from Escherichia coli. J Bacteriol. déc 1989;171(12):6862–6. 10.1128/jb.171.12.6862-6866.1989 PMC210589 2480345

[67] Dong H, Nilsson L, Kurland CG. Co-variation of tRNA abundance and codon usage in Escherichia coli at different growth rates. J Mol Biol. 2 août 1996;260(5):649–63. doi: 10.1006/jmbi.1996.0428 8709146

[68] Sørensen MA, Fehler AO, Lo Svenningsen S. Transfer RNA instability as a stress response in Escherichia coli: Rapid dynamics of the tRNA pool as a function of demand. RNA Biol. 9 mai 2018;15(4–5):586–93. doi: 10.1080/15476286.2017.1391440 29023189 PMC6103710

[69] RNA quality control: degradation of defective transfer RNA. The EMBO Journal. 1 mars 2002;21(5):1132–8. doi: 10.1093/emboj/21.5.1132 11867541 PMC125898

[70] Sun X, Yang J, Yu M, Yao D, Zhou L, Li X, et al. Global identification and characterization of tRNA-derived RNA fragment landscapes across human cancers. NAR Cancer. 19 oct 2020;2(4):zcaa031. doi: 10.1093/narcan/zcaa031 34316691 PMC8210304

[71] Salvail H, Lanthier-Bourbonnais P, Sobota JM, Caza M, Benjamin JAM, Mendieta MES, et al. A small RNA promotes siderophore production through transcriptional and metabolic remodeling. Proc Natl Acad Sci U S A. 24 août 2010;107(34):15223–8. doi: 10.1073/pnas.1007805107 20696910 PMC2930555

[72] Gogol EB, Rhodius VA, Papenfort K, Vogel J, Gross CA. Small RNAs endow a transcriptional activator with essential repressor functions for single-tier control of a global stress regulon. PNAS. 2 août 2011;108(31):12875–80. doi: 10.1073/pnas.1109379108 21768388 PMC3150882

[73] Mackie GA. RNase E: at the interface of bacterial RNA processing and decay. Nat Rev Microbiol. janv 2013;11(1):45–57. doi: 10.1038/nrmicro2930 23241849

[74] Ow MC, Kushner SR. Initiation of tRNA maturation by RNase E is essential for cell viability in E. coli. Genes Dev. 1 mai 2002;16(9):1102–15. doi: 10.1101/gad.983502 12000793 PMC186257

[75] Tamura M, Moore CJ, Cohen SN. Nutrient Dependence of RNase E Essentiality in Escherichia coli. J Bacteriol. mars 2013;195(6):1133–41. doi: 10.1128/JB.01558-12 23275245 PMC3591997

[76] Söderbom F, Svärd SG, Kirsebom LA. RNase E cleavage in the 5’ leader of a tRNA precursor. J Mol Biol. 9 sept 2005;352(1):22–7. doi: 10.1016/j.jmb.2005.07.003 16081101

[77] Mohanty BK, Agrawal A, Kushner SR. Generation of pre-tRNAs from polycistronic operons is the essential function of RNase P in Escherichia coli. Nucleic Acids Research. 18 mars 2020;48(5):2564–78. doi: 10.1093/nar/gkz1188 31993626 PMC7049720

[78] Nakajima N, Ozeki H, Shimura Y. Organization and structure of an E. coli tRNA operon containing seven tRNA genes. Cell. janv 1981;23(1):239–49. 10.1016/0092-8674(81)90288-9 6163550

[79] Antón AI, Martínez-Murcia AJ, Rodríguez-Valera F. Sequence diversity in the 16S-23S intergenic spacer region (ISR) of the rRNA operons in representatives of the Escherichia coli ECOR collection. J Mol Evol. juill 1998;47(1):62–72. doi: 10.1007/pl00006363 9664697

[80] Magray MSUD, Kumar A, Rawat AK, Srivastava S. Identification of Escherichia coli through analysis of 16S rRNA and 16S-23S rRNA internal transcribed spacer region sequences. Bioinformation. 2 août 2011;6(10):370–1. doi: 10.6026/97320630006370 21904423 PMC3163913

[81] Sekiya T, Nishimura S. Sequence of the gene for isoleucine tRNA1 and the surrounding region in a ribosomal RNA operon of Escherichia coli. Nucleic Acids Res. févr 1979;6(2):575–92. doi: 10.1093/nar/6.2.575 370791 PMC327714

[82] Shajani Z, Sykes MT, Williamson JR. Assembly of bacterial ribosomes. Annu Rev Biochem. 2011;80:501–26. doi: 10.1146/annurev-biochem-062608-160432 21529161

[83] Loughney K, Lund E, Dahlberg JE. tRNA genes are found between the 16S and 23S rRNA genes in Bacillus subtilis. Nucleic Acids Research. 11 mars 1982;10(5):1607–24. 6280153 10.1093/nar/10.5.1607 PMC320553

[84] Condon C, Liveris D, Squires C, Schwartz I, Squires CL. rRNA operon multiplicity in Escherichia coli and the physiological implications of rrn inactivation. J Bacteriol. juill 1995;177(14):4152–6. doi: 10.1128/jb.177.14.4152-4156.1995 7608093 PMC177152

[85] Blenkiron C, Simonov D, Muthukaruppan A, Tsai P, Dauros P, Green S, et al. Uropathogenic Escherichia coli Releases Extracellular Vesicles That Are Associated with RNA. Cascales E, éditeur. PLOS ONE. 8 août 2016;11(8):e0160440. doi: 10.1371/journal.pone.0160440 27500956 PMC4976981

[86] Resch U, Tsatsaronis JA, Le Rhun A, Stübiger G, Rohde M, Kasvandik S, et al. A Two-Component Regulatory System Impacts Extracellular Membrane-Derived Vesicle Production in Group A Streptococcus. mBio. 1 nov 2016;7(6):e00207–16. doi: 10.1128/mBio.00207-16 27803183 PMC5090034

[87] O’Donoghue EJ, Krachler AM. Mechanisms of outer membrane vesicle entry into host cells. Cell Microbiol. nov 2016;18(11):1508–17. doi: 10.1111/cmi.12655 27529760 PMC5091637

[88] Shi N, Li N, Duan X, Niu H. Interaction between the gut microbiome and mucosal immune system. Mil Med Res. 27 avr 2017;4:14. doi: 10.1186/s40779-017-0122-9 28465831 PMC5408367

[89] Zheng J, Lou L, Fan J, Huang C, Mei Q, Wu J, et al. Commensal Escherichia coli Aggravates Acute Necrotizing Pancreatitis through Targeting of Intestinal Epithelial Cells. Appl Environ Microbiol. 30 mai 2019;85(12):e00059–19. doi: 10.1128/AEM.00059-19 30979838 PMC6544826

[90] Kuscu C, Kumar P, Kiran M, Su Z, Malik A, Dutta A. tRNA fragments (tRFs) guide Ago to regulate gene expression post-transcriptionally in a Dicer-independent manner. RNA. août 2018;24(8):1093–105. doi: 10.1261/rna.066126.118 29844106 PMC6049499

[91] Eigenbrod T, Dalpke AH. Bacterial RNA: An Underestimated Stimulus for Innate Immune Responses. J Immunol. 15 juill 2015;195(2):411–8. doi: 10.4049/jimmunol.1500530 26138638

[92] Fortelny N, Overall CM, Pavlidis P, Freue GVC. Can we predict protein from mRNA levels? Nature. juill 2017;547(7664):E19–20. doi: 10.1038/nature22293 28748932

[93] Kondoh K, Nishida E. Regulation of MAP kinases by MAP kinase phosphatases. Biochimica et Biophysica Acta (BBA)—Molecular Cell Research. 1 août 2007;1773(8):1227–37. doi: 10.1016/j.bbamcr.2006.12.002 17208316

[94] Barquist L, Vogel J. Accelerating Discovery and Functional Analysis of Small RNAs with New Technologies. Annual Review of Genetics. 2015;49(1):367–94. doi: 10.1146/annurev-genet-112414-054804 26473381

[95] Plante I, Plé H, Landry P, Gunaratne PH, Provost P. Modulation of microRNA Activity by Semi-microRNAs. Front Genet. 2012;3:99. doi: 10.3389/fgene.2012.00099 22675332 PMC3366366

[96] Herrera-Carrillo E, Berkhout B. Dicer-independent processing of small RNA duplexes: mechanistic insights and applications. Nucleic Acids Res. 13 oct 2017;45(18):10369–79. doi: 10.1093/nar/gkx779 28977573 PMC5737282

[97] Huang HY, Lin YCD, Li J, Huang KY, Shrestha S, Hong HC, et al. miRTarBase 2020: updates to the experimentally validated microRNA-target interaction database. Nucleic Acids Res. 8 janv 2020;48(D1):D148–54. doi: 10.1093/nar/gkz896 31647101 PMC7145596

[98] Kurzynska-Kokorniak A, Koralewska N, Pokornowska M, Urbanowicz A, Tworak A, Mickiewicz A, et al. The many faces of Dicer: the complexity of the mechanisms regulating Dicer gene expression and enzyme activities. Nucleic Acids Res. 19 mai 2015;43(9):4365–80. doi: 10.1093/nar/gkv328 25883138 PMC4482082

[99] Saetrom P, Heale BSE, Snøve O, Aagaard L, Alluin J, Rossi JJ. Distance constraints between microRNA target sites dictate efficacy and cooperativity. Nucleic Acids Res. 2007;35(7):2333–42. doi: 10.1093/nar/gkm133 17389647 PMC1874663

[100] Lytle JR, Yario TA, Steitz JA. Target mRNAs are repressed as efficiently by microRNA-binding sites in the 5′ UTR as in the 3′ UTR. PNAS. 5 juin 2007;104(23):9667–72. doi: 10.1073/pnas.0703820104 17535905 PMC1887587

[101] Connelly CM, Thomas M, Deiters A. High-Throughput Luciferase Reporter Assay for Small-Molecule Inhibitors of MicroRNA Function. J Biomol Screen. juill 2012;17(6):822–8. doi: 10.1177/1087057112439606 22412086 PMC3758890

[102] Gerwins P, Blank JL, Johnson GL. Cloning of a novel mitogen-activated protein kinase kinase kinase, MEKK4, that selectively regulates the c-Jun amino terminal kinase pathway. J Biol Chem. 28 mars 1997;272(13):8288–95. doi: 10.1074/jbc.272.13.8288 9079650

[103] Comes F, Matrone A, Lastella P, Nico B, Susca FC, Bagnulo R, et al. A novel cell type-specific role of p38 α in the control of autophagy and cell death in colorectal cancer cells. Cell Death & Differentiation. avr 2007;14(4):693–702. 17159917 10.1038/sj.cdd.4402076

[104] Cargnello M, Roux PP. Activation and Function of the MAPKs and Their Substrates, the MAPK-Activated Protein Kinases. Microbiol Mol Biol Rev. mars 2011;75(1):50–83. doi: 10.1128/MMBR.00031-10 21372320 PMC3063353

[105] Craig EA, Stevens MV, Vaillancourt RR, Camenisch TD. MAP3Ks as central regulators of cell fate during development. Developmental Dynamics. 2008;237(11):3102–14. doi: 10.1002/dvdy.21750 18855897

[106] Yang LX, Gao Q, Shi JY, Wang ZC, Zhang Y, Gao PT, et al. Mitogen-activated protein kinase kinase kinase 4 deficiency in intrahepatic cholangiocarcinoma leads to invasive growth and epithelial-mesenchymal transition. Hepatology. 2015;62(6):1804–16. doi: 10.1002/hep.28149 26340507

[107] Ismail S, Hampton MB, Keenan JI. Helicobacter pylori outer membrane vesicles modulate proliferation and interleukin-8 production by gastric epithelial cells. Infect Immun. oct 2003;71(10):5670–5. doi: 10.1128/IAI.71.10.5670-5675.2003 14500487 PMC201067

[108] Vidakovics MLAP, Jendholm J, Mörgelin M, Månsson A, Larsson C, Cardell LO, et al. B Cell Activation by Outer Membrane Vesicles—A Novel Virulence Mechanism. PLOS Pathogens. 15 janv 2010;6(1):e1000724. doi: 10.1371/journal.ppat.1000724 20090836 PMC2799554

[109] Dhital S, Deo P, Stuart I, Naderer T. Bacterial outer membrane vesicles and host cell death signaling. Trends Microbiol. déc 2021;29(12):1106–16. doi: 10.1016/j.tim.2021.04.003 34001418

[110] Beatty M, Guduric-Fuchs J, Brown E, Bridgett S, Chakravarthy U, Hogg RE, et al. Small RNAs from plants, bacteria and fungi within the order Hypocreales are ubiquitous in human plasma. BMC Genomics. 25 oct 2014;15:933. doi: 10.1186/1471-2164-15-933 25344700 PMC4230795

[111] Wang K, Li H, Yuan Y, Etheridge A, Zhou Y, Huang D, et al. The Complex Exogenous RNA Spectra in Human Plasma: An Interface with Human Gut Biota? PLOS ONE. 10 déc 2012;7(12):e51009. doi: 10.1371/journal.pone.0051009 23251414 PMC3519536

[112] Liu S, da Cunha AP, Rezende RM, Cialic R, Wei Z, Bry L, et al. The Host Shapes the Gut Microbiota via Fecal microRNA. Cell Host Microbe. 13 janv 2016;19(1):32–43. doi: 10.1016/j.chom.2015.12.005 26764595 PMC4847146

[113] Longo PA, Kavran JM, Kim MS, Leahy DJ. Transient Mammalian Cell Transfection with Polyethylenimine (PEI). Methods Enzymol. 2013;529:227–40. doi: 10.1016/B978-0-12-418687-3.00018-5 24011049 PMC4012321

[114] Dalby B, Cates S, Harris A, Ohki EC, Tilkins ML, Price PJ, et al. Advanced transfection with Lipofectamine 2000 reagent: primary neurons, siRNA, and high-throughput applications. Methods. juin 2004;33(2):95–103. 10.1016/j.ymeth.2003.11.023 15121163

[115] Chutkan H, Macdonald I, Manning A, Kuehn MJ. Quantitative and qualitative preparations of bacterial outer membrane vesicles. Methods Mol Biol. 2013;966:259–72. doi: 10.1007/978-1-62703-245-2_16 23299740 PMC4317262

[116] Klimentová J, Stulík J. Methods of isolation and purification of outer membrane vesicles from gram-negative bacteria. Microbiol Res. janv 2015;170:1–9. doi: 10.1016/j.micres.2014.09.006 25458555

[117] Aiba H, Adhya S, Crombrugghe B de. Evidence for two functional gal promoters in intact Escherichia coli cells. J Biol Chem. 25 nov 1981;256(22):11905–10. 6271763

[118] Ye J, Coulouris G, Zaretskaya I, Cutcutache I, Rozen S, Madden TL. Primer-BLAST: A tool to design target-specific primers for polymerase chain reaction. BMC Bioinformatics. 18 juin 2012;13:134. doi: 10.1186/1471-2105-13-134 22708584 PMC3412702

[119] Livak KJ, Schmittgen TD. Analysis of relative gene expression data using real-time quantitative PCR and the 2(-Delta Delta C(T)) Method. Methods. déc 2001;25(4):402–8. doi: 10.1006/meth.2001.1262 11846609

[120] Stark MR, Pleiss JA, Deras M, Scaringe SA, Rader SD. An RNA ligase-mediated method for the efficient creation of large, synthetic RNAs. RNA. nov 2006;12(11):2014–9. doi: 10.1261/rna.93506 16983143 PMC1624903

[121] Stark MR, Rader SD. Efficient splinted ligation of synthetic RNA using RNA ligase. Methods Mol Biol. 2014;1126:137–49. doi: 10.1007/978-1-62703-980-2_10 24549661

[122] Lambert M, Benmoussa A, Provost P. A New Specific and Sensitive RT-qPCR Method Based on Splinted 5′ Ligation for the Quantitative Detection of RNA Species Shorter than microRNAs. Non-Coding RNA. sept 2021;7(3):59. doi: 10.3390/ncrna7030059 34564321 PMC8482087

[123] Vester B, Wengel J. LNA (locked nucleic acid): high-affinity targeting of complementary RNA and DNA. Biochemistry. 26 oct 2004;43(42):13233–41. doi: 10.1021/bi0485732 15491130

[124] RIPA Lysis Buffer. Cold Spring Harb Protoc. 12 janv 2017;2017(12):pdb.rec101428.

[125] Schneider CA, Rasband WS, Eliceiri KW. NIH Image to ImageJ: 25 years of image analysis. Nat Methods. juill 2012;9(7):671–5. doi: 10.1038/nmeth.2089 22930834 PMC5554542

