# Supplementary material for: A tRNA-derived fragment present in E. coli OMVs regulates host cell gene expression and proliferation
Source: PLoS Pathog. 2022 Sep 15;18(9):e1010827. doi: 10.1371/journal.ppat.1010827 (PMC9514646; doi:10.1371/journal.ppat.1010827)
Supplement: S1 Fig — Determination of the optimal measurement time and the appropriate amount of cells. (DOCX) [file ppat.1010827.s001.docx]

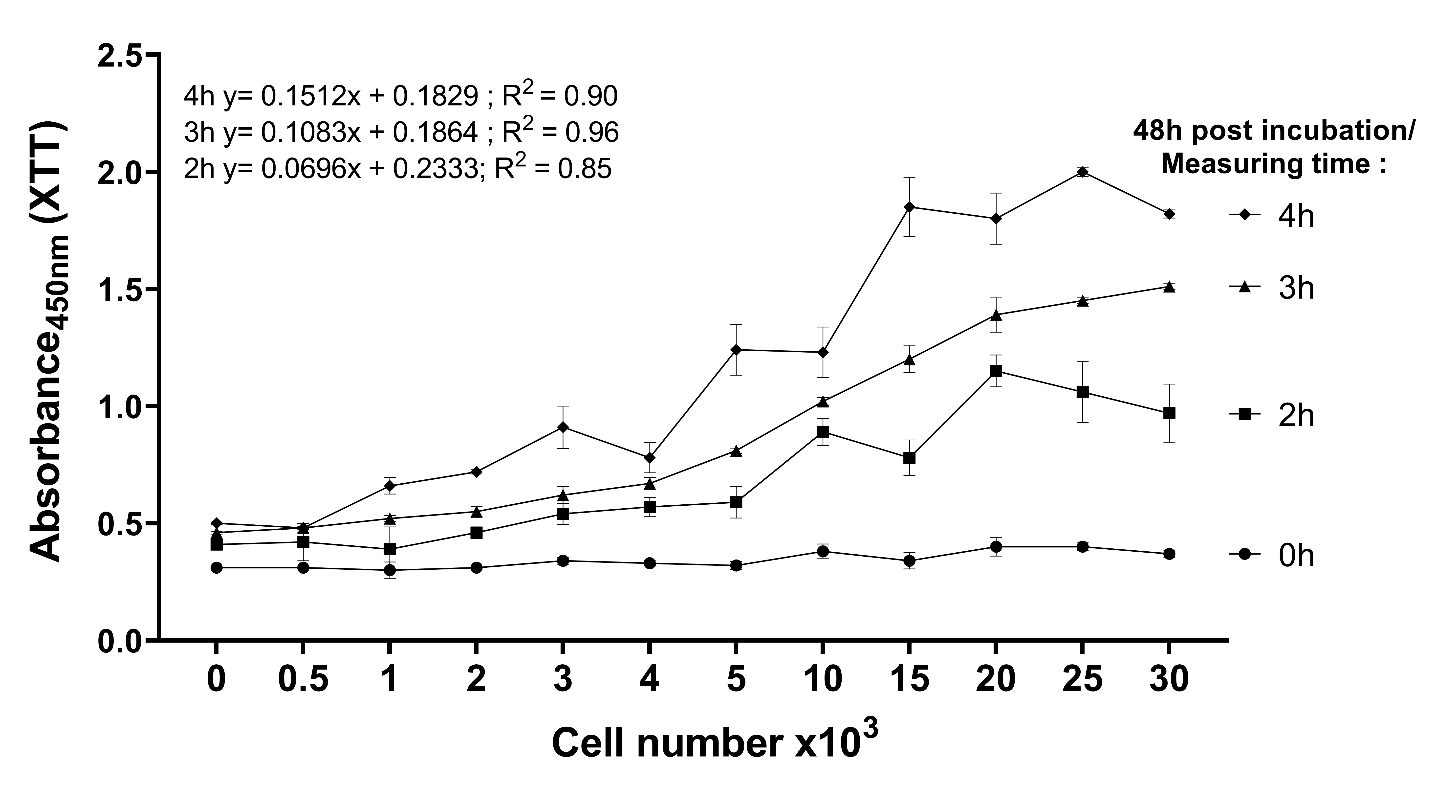


**Supplementary Figure S1. Setting up of the XTT test of cell proliferation.** Determination of the optimal measurement time and the appropriate amount of cells
