# Supplementary material for: A tRNA-derived fragment present in E. coli OMVs regulates host cell gene expression and proliferation
Source: PLoS Pathog. 2022 Sep 15;18(9):e1010827. doi: 10.1371/journal.ppat.1010827 (PMC9514646; doi:10.1371/journal.ppat.1010827)
Supplement: S2 Fig — (A) E. coli MG1655 bacteria were grown at 37°C up to the exponential (Reference, R) and stationary phases of growth in either complete (rich) LB or minimal M63 medium. (B) E. coli MG1655 bacteria were grown in LB medium at exponential phase (R) in different temperatures: 30, 37 and or 44°C. (C) Bacterial mRNA or protein synthesis was inhibited by addition of chloramphenicol or rifampicin, respectively, to cultures of E. coli MG1655 grown at exponential phase (R). The level of bacterial Ile-tRF-5X level is not modulated by transcription or translation activity (D) E. coli strains carrying heat-sensitive (hs) mutations in the essential genes rne-3071-hs (EM1277) and rnpA-hs (KP1036) were grown in LB medium at 30°C and then heat-shocked (44°C) to transiently inhibit ribonuclease (RNase) P or RNase E, which are involved in tRNA maturation. Bacteria RNase E contributes to Ile-tRF-5X biogenesis. For more details, see Supplementary S1 Table. In all conditions, the level of Ile-tRF-5X and Ile-tRNA were measured by LNA RT-qPCR. A spike-in (UniSp6) and reference genes (23S and/or 16S) were used as control and for normalization. The results are reported in fold change compared to the reference condition. Statistical analysis. Data were calculated from three biological replicate measurements (n = 3; mean ± SD). Two-way analysis of variance (ANOVA) and Dunnett’s multiple comparisons (fold change vs. reference test) or Šídák’s multiple comparisons test (Ile-tRF-5X vs. Ile-tRNA) were used for statistical analysis. Statistically significant differences (fold change vs. reference or Ile-tRF-5X vs. Ile-tRNA shown in purple) are indicated by stars (*), * p < 0.05; ** p < 0.01; *** p < 0.001; **** p < 0.0001; ns, not significant. (DOCX) [file ppat.1010827.s002.docx]

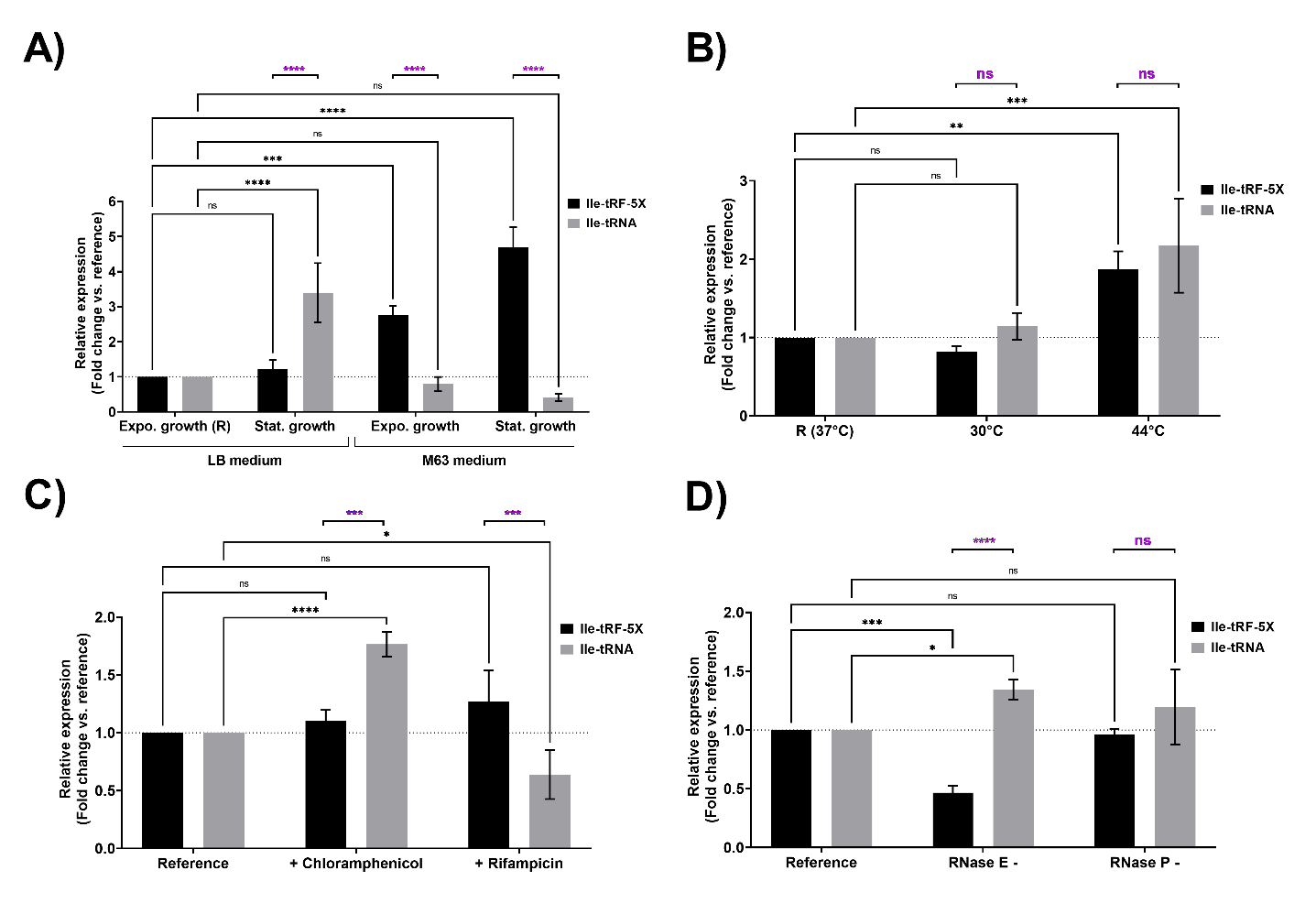


**Figure S2. Bacterial Ile-tRF-5X and mature Ile-tRNA levels under different experimental conditions**. (**A**) *E. coli* MG1655 bacteria were grown at 37°C up to the exponential (Reference, R) and stationary phases of growth in either complete (rich) LB or minimal M63 medium. (**B**) *E. coli* MG1655 bacteria were grown in LB medium at exponential phase (R) in different temperatures: 30, 37 and or 44°C. (**C**) Bacterial mRNA or protein synthesis was inhibited by addition of chloramphenicol or rifampicin, respectively, to cultures of *E. coli* MG1655 grown at exponential phase (R). The level of bacterial Ile-tRF-5X level is not modulated by transcription or translation activity (**D**) *E. coli* strains carrying heat-sensitive (hs) mutations in the essential genes rne-3071-hs (EM1277) and rnpA-hs (KP1036) were grown in LB medium at 30°C and then heat-shocked (44°C) to transiently inhibit ribonuclease (RNase) P or RNase E, which are involved in tRNA maturation. Bacteria RNase E contributes to Ile-tRF-5X biogenesis. For more details, see **Supplementary Table S1**. In all conditions, the level of Ile-tRF-5X and Ile-tRNA were measured by LNA RT-qPCR. A spike-in (UniSp6) and reference genes (23S and/or 16S) were used as control and for normalization. The results are reported in fold change compared to the reference condition. **Statistical analysis**. Data were calculated from three biological replicate measurements (n=3; mean ± SD). Two-way analysis of variance (ANOVA) and Dunnett's multiple comparisons (fold change vs. reference test) or Šídák’s multiple comparisons test (Ile-tRF-5X vs. Ile-tRNA) were used for statistical analysis. Statistically significant differences (fold change vs. reference or Ile-tRF-5X vs. Ile-tRNA shown in purple) are indicated by stars (*), * p < 0.05; ** p < 0.01; *** p < 0.001; **** p < 0.0001; ns, not significant.
