# Supplementary material for: A tRNA-derived fragment present in E. coli OMVs regulates host cell gene expression and proliferation
Source: PLoS Pathog. 2022 Sep 15;18(9):e1010827. doi: 10.1371/journal.ppat.1010827 (PMC9514646; doi:10.1371/journal.ppat.1010827)
Supplement: S3 Fig — Ile-tRF-5X was quantified from normalized RNA-Seq data in E. coli exponential growth phase (reference, ref, R), stationary phase, and after treatment with chloramphenicol (R+cat), rifampicin (R+rif) or heat shock to transiently inactivate RNase E (RNase E -). Fig 2A (LB medium), Fig 3 and Fig 4 (RNase E) are the quantitative validation of these data. One biological replicate. See [reference [1]] for more details. (DOCX) [file ppat.1010827.s003.docx]

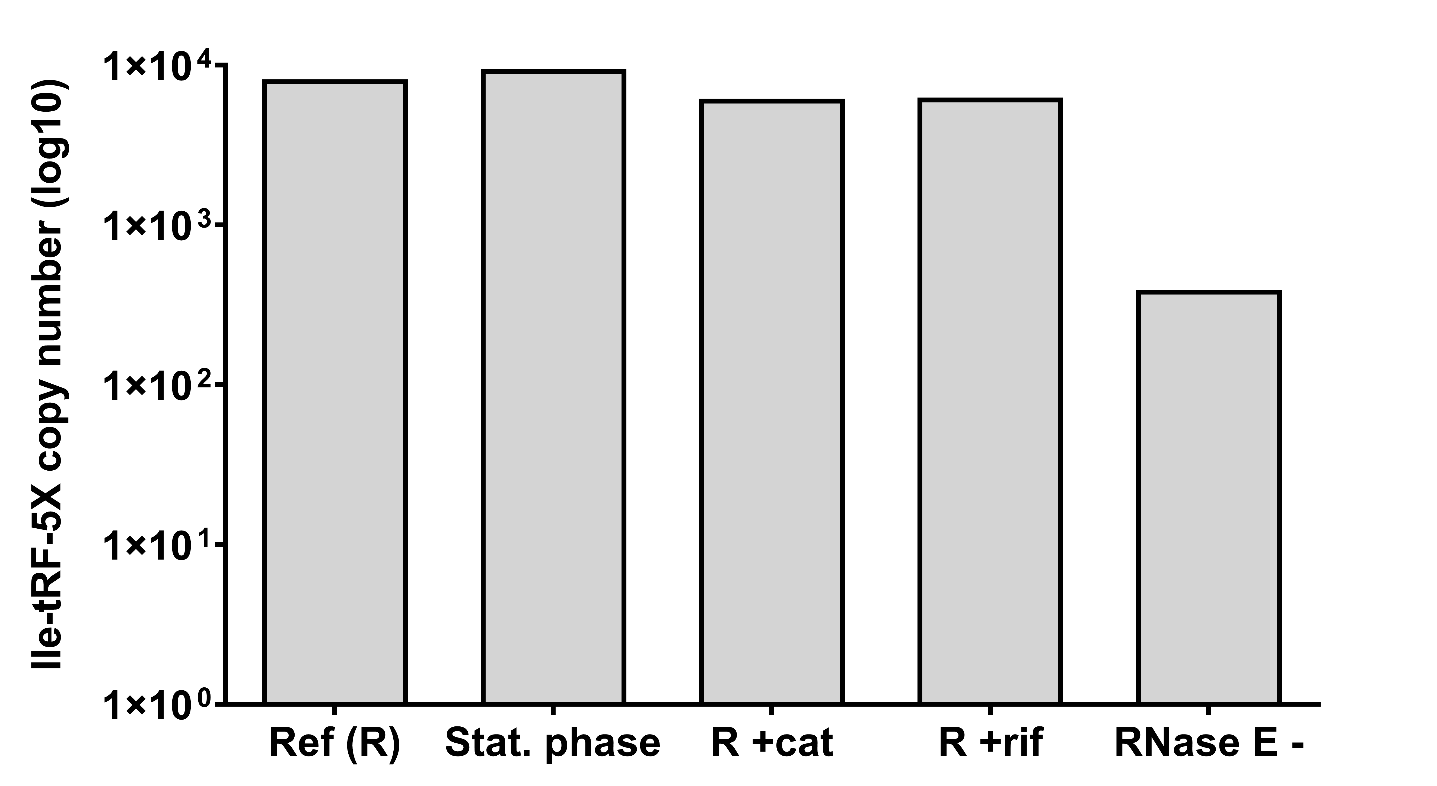


**Supplementary Figure S3. Ile-tRF-5X level in different E. coli samples**. Ile-tRF-5X was quantified from normalized RNA-Seq data in *E. coli* exponential growth phase (reference, ref, R), stationary phase, and after treatment with chloramphenicol (R+cat), rifampicin (R+rif) or heat shock to transiently inactivate RNase E (RNase E -). Figure 2A (LB medium), Figure 3 and Figure 4 (RNase E) are the quantitative validation of these data. One biological replicate. See reference [1] for more details.

References

1. Diallo, I.; Ho, J.; Lalaouna, D.; Massé, E.; Provost, P. RNA Sequencing Unveils Very Small RNAs With Potential Regulatory Functions in Bacteria. *Frontiers in Molecular Biosciences* **2022**, *9*.
