# Supplementary material for: A tRNA-derived fragment present in E. coli OMVs regulates host cell gene expression and proliferation
Source: PLoS Pathog. 2022 Sep 15;18(9):e1010827. doi: 10.1371/journal.ppat.1010827 (PMC9514646; doi:10.1371/journal.ppat.1010827)
Supplement: S4 Fig — (DOCX) [file ppat.1010827.s004.docx]

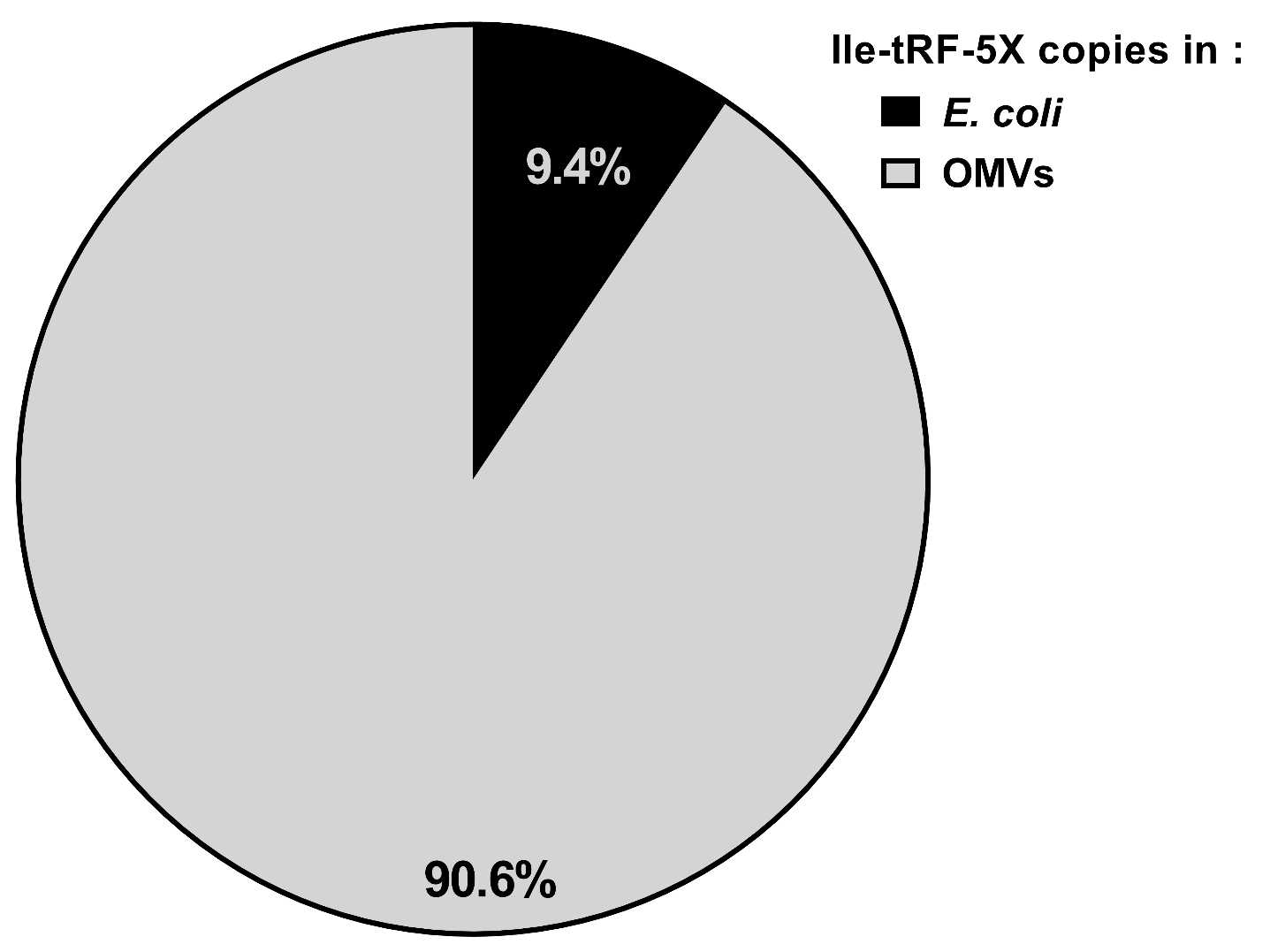


**Supplementary Figure S4. Proportion in copy number of intracellular Ile-tRF-5X or OMV-packaged Ile-tRF-5X.**
