# Supplementary material for: A tRNA-derived fragment present in E. coli OMVs regulates host cell gene expression and proliferation
Source: PLoS Pathog. 2022 Sep 15;18(9):e1010827. doi: 10.1371/journal.ppat.1010827 (PMC9514646; doi:10.1371/journal.ppat.1010827)
Supplement: S5 Fig — HCT116 cells were stained with a Cell Tracker CMTPX™ (red) and then incubated for 2 h with bacterial OMVs labelled with PKH67 (green). The nuclei are stained with DAPI (blue). The XZ and YZ projection of Volocity shows that OMVs were internalized by HCT116 cells and localized mainly in the cytoplasm (See panel A at 2 h in Fig 5.). (DOCX) [file ppat.1010827.s005.docx]

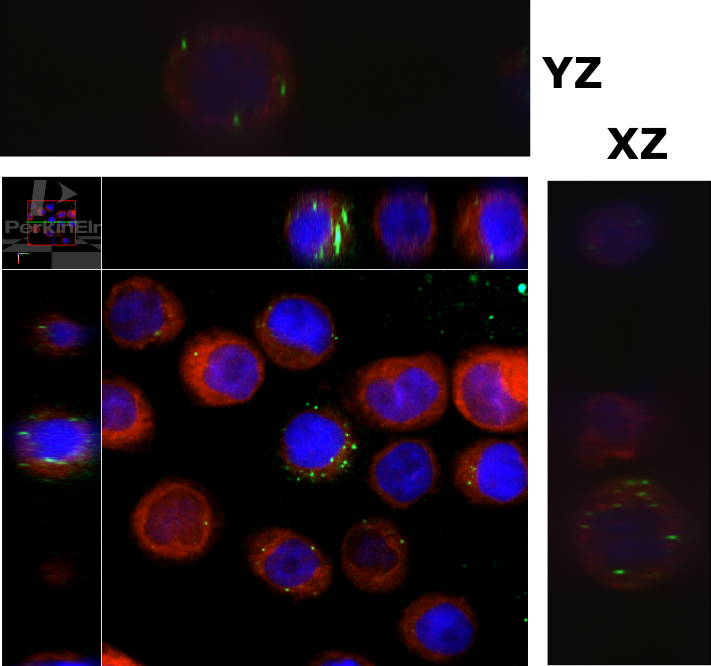


**Supplementary Figure S5. Confocal microscopy imaging of labelled OMVs taken up by human HCT116 cells**. HCT116 cells were stained with a Cell Tracker CMTPX™ (red) and then incubated for 2 h with bacterial OMVs labelled with PKH67 (green). The nuclei are stained with DAPI (blue). The XZ and YZ projection of Volocity shows that OMVs were internalized by HCT116 cells and localized mainly in the cytoplasm (See panel A at 2 h in figure 5.).
