# Supplementary material for: A tRNA-derived fragment present in E. coli OMVs regulates host cell gene expression and proliferation
Source: PLoS Pathog. 2022 Sep 15;18(9):e1010827. doi: 10.1371/journal.ppat.1010827 (PMC9514646; doi:10.1371/journal.ppat.1010827)
Supplement: S6 Fig — Three bioinformatics tools (microRT, blastN and RNAhybrid) used in combination predict several Ile-tRF-5X (5X) binding sites in the human MAP3K4 mRNA. Base pairing of the top three Ile-tRF-5X binding sites is shown on the left. The number of nucleotides (mer) involved in the interaction (seed sequences), their minimal free energy (MFE, according to RNAhybrid) and their position (5’UTR, ORF or 3’UTR) are listed in the table. * indicates that the 13th nt of Ile-tRF-5X is a C, thus allowing perfect base pairing with MAP3K4 mRNA in its ORF. (DOCX) [file ppat.1010827.s006.docx]

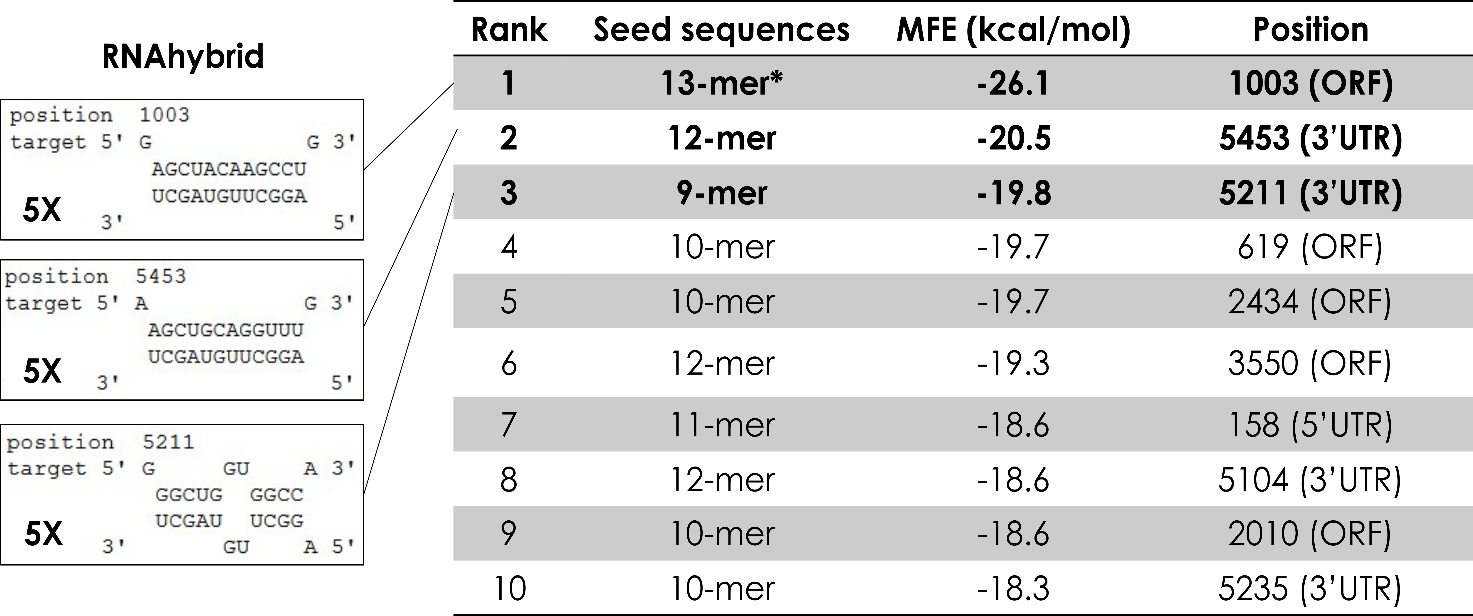


**Supplementary Figure S6. Human MAP3K4 mRNA harbors potential binding sites for bacterial Ile-tRF-5X.** Three bioinformatics tools (microRT, blastN and RNAhybrid) used in combination predict several Ile-tRF-5X (5X) binding sites in the human MAP3K4 mRNA. Base pairing of the top three Ile-tRF-5X binding sites is shown on the left. The number of nucleotides (mer) involved in the interaction (seed sequences), their minimal free energy (MFE, according to RNAhybrid) and their position (5’UTR, ORF or 3’UTR) are listed in the table. * indicates that the 13th nt of Ile-tRF-5X is a C, thus allowing perfect base pairing with MAP3K4 mRNA in its ORF.
