# Supplementary material for: A tRNA-derived fragment present in E. coli OMVs regulates host cell gene expression and proliferation
Source: PLoS Pathog. 2022 Sep 15;18(9):e1010827. doi: 10.1371/journal.ppat.1010827 (PMC9514646; doi:10.1371/journal.ppat.1010827)
Supplement: S7 Fig — The base pairing interactions between bacterial Ile-tRF-5X and human MAP3K4 mRNA (RNAhybrid) are shown in red, with the minimum free energy (Mfe) and position (nucleotides). The base pairing interactions between human miRNAs and MAP3K4 mRNA (Targetscan) are highlighted in grey. The nucleotides involved in the Watson–Crick base pairing are in bold. The numbers in square brackets ([]) correspond to the number of non-displayed nucleotides in the 3’UTR of MAP3K4 mRNA. The full MAP3K4 mRNA 3′ UTR sequence was cloned downstream of the humanized Rluc (hRluc) gene, in the dual-luciferase reporter gene expression vector psiCHECK-II, with hFluc as a normalization control. hRluc, humanized Renilla luciferase gene; hluc+, humanized Firefly luciferase gene. (DOCX) [file ppat.1010827.s007.docx]

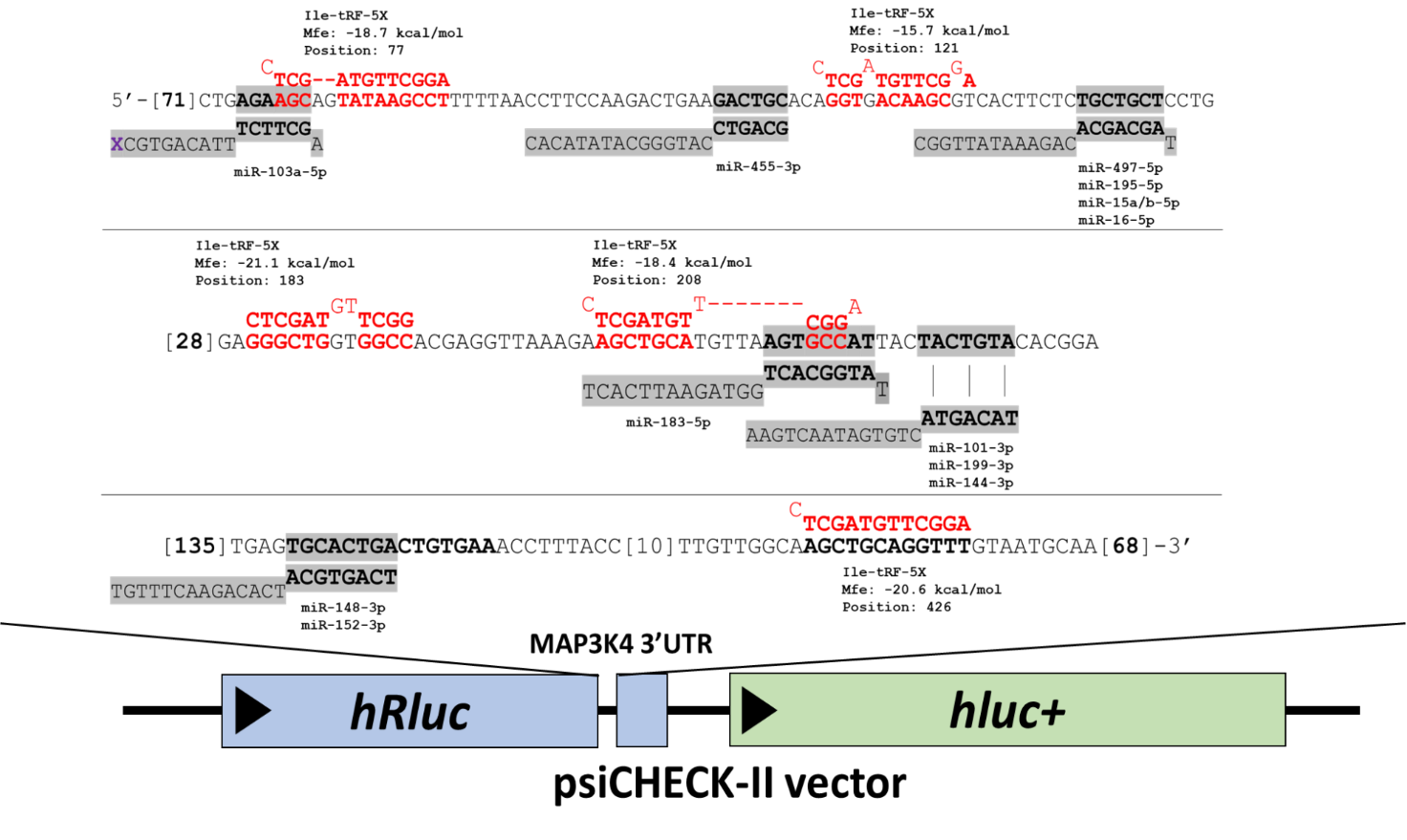


**Supplementary Figure S7. Schematic representation of the predicted Ile-tRF-5X and miRNA base pairing in the MAP3K4 mRNA 3’UTR**. The base pairing interactions between bacterial Ile-tRF-5X and human MAP3K4 mRNA (RNAhybrid) are shown in red, with the minimum free energy (Mfe) and position (nucleotides). The base pairing interactions between human miRNAs and MAP3K4 mRNA (Targetscan) are highlighted in grey. The nucleotides involved in the Watson–Crick base pairing are in bold. The numbers in square brackets ([]) correspond to the number of non-displayed nucleotides in the 3’UTR of MAP3K4 mRNA. The full MAP3K4 mRNA 3′ UTR sequence was cloned downstream of the humanized Rluc (hRluc) gene, in the dual-luciferase reporter gene expression vector psiCHECK-II, with hFluc as a normalization control. hRluc, humanized *Renilla luciferase* gene; hluc+, humanized *Firefly luciferase* gene.
