# Supplementary material for: A tRNA-derived fragment present in E. coli OMVs regulates host cell gene expression and proliferation
Source: PLoS Pathog. 2022 Sep 15;18(9):e1010827. doi: 10.1371/journal.ppat.1010827 (PMC9514646; doi:10.1371/journal.ppat.1010827)
Supplement: S8 Fig — FORNA Web-based tools were used to illustrate RNA secondary structure of the full 3’UTR of MAP3K4. In blue we have the binding site positions of miRNAs and in red those of Ile-tRF-5X. For each of these actors, the positions (P) of the nucleotides (nt) are indicated. (DOCX) [file ppat.1010827.s008.docx]

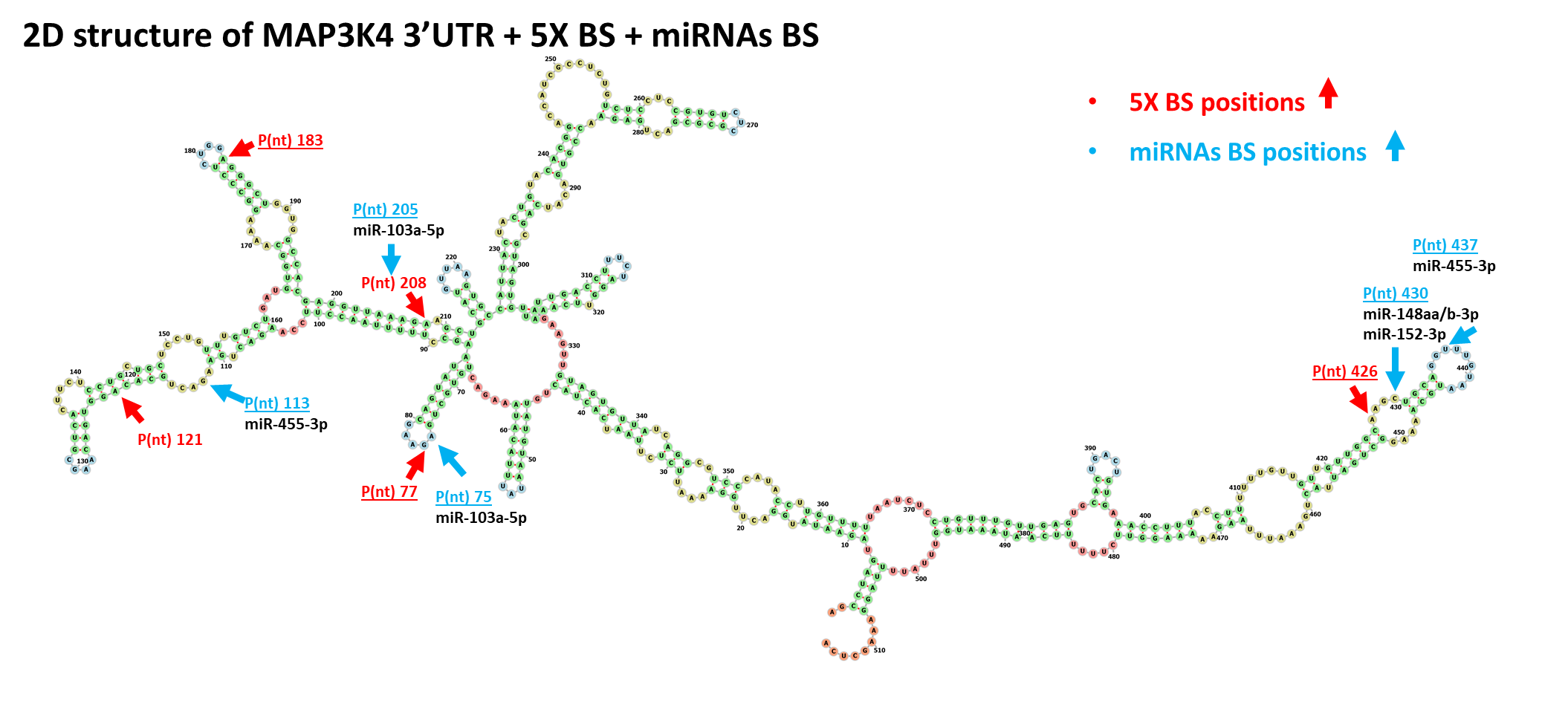


**Supplementary Figure S8. Secondary structure of 3’UTR MAP3K4 displaying the binding sites of miRNAs and Ile-tRF-5X**. FORNA Web-based tools were used to illustrate RNA secondary structure of the full 3’UTR of MAP3K4. In blue we have the binding site positions of miRNAs and in red those of Ile-tRF-5X. For each of these actors, the positions (P) of the nucleotides (nt) are indicated.
