# Supplementary material for: A tRNA-derived fragment present in E. coli OMVs regulates host cell gene expression and proliferation
Source: PLoS Pathog. 2022 Sep 15;18(9):e1010827. doi: 10.1371/journal.ppat.1010827 (PMC9514646; doi:10.1371/journal.ppat.1010827)
Supplement: S9 Fig — Cell number was estimated from XTT-based absorbance (450 nm) measurement. The % of cell proliferation was deduced from that of the mock control set at 100%. The use of Ile-tRF-5X (as5X, 100nM) did not significantly reduce cell proliferation. Each data set is normalized with its corresponding control. Statistical analysis. All data presented were calculated from three biological replicate (n = 3) measurements ± SD and each sample was tested with 3 replicates. The one-way analysis of variance (ANOVA) and Holm-Šídák’s multiple comparisons test were used for statistical analysis. Statistically significant differences (fold change vs. mock) are indicated as follows: * p < 0.05; *** p < 0.001; **** p < 0.0001. (DOCX) [file ppat.1010827.s009.docx]

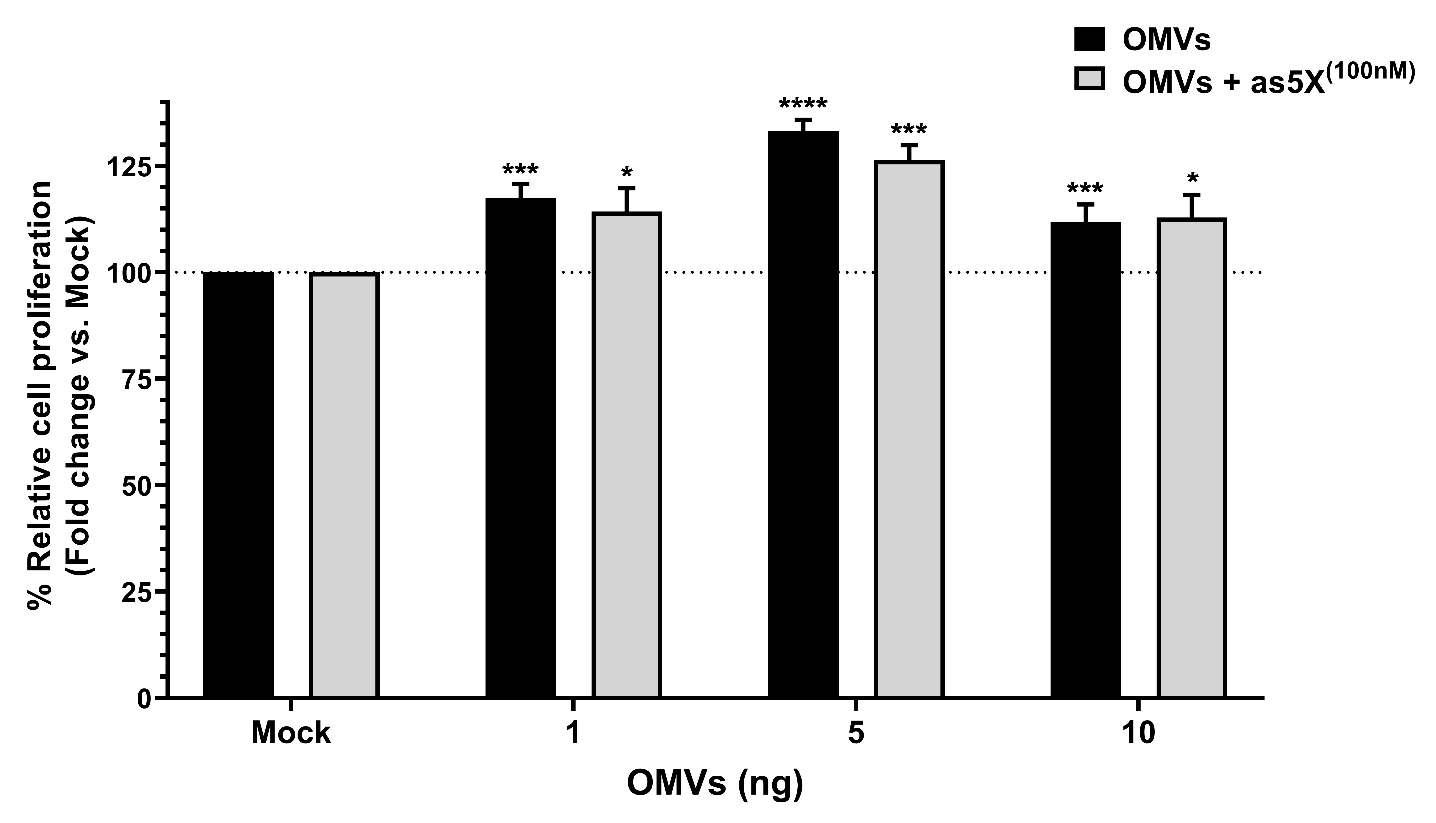


**Supplementary Figure S9.** **OMVs enhance HCT116 cell proliferation**. Cell number was estimated from XTT-based absorbance (450 nm) measurement. The % of cell proliferation was deduced from that of the mock control set at 100%. The use of Ile-tRF-5X (as5X, 100nM) did not significantly reduce cell proliferation. Each data set is normalized with its corresponding control. **Statistical analysis**. All data presented were calculated from three biological replicate (n = 3) measurements ± SD and each sample was tested with 3 replicates. The one-way analysis of variance (ANOVA) and Holm-Šídák's multiple comparisons test were used for statistical analysis. Statistically significant differences (fold change vs. mock) are indicated as follows: * p < 0.05; *** p < 0.001; **** p < 0.0001.
