# Supplementary material for: A tRNA-derived fragment present in E. coli OMVs regulates host cell gene expression and proliferation
Source: PLoS Pathog. 2022 Sep 15;18(9):e1010827. doi: 10.1371/journal.ppat.1010827 (PMC9514646; doi:10.1371/journal.ppat.1010827)
Supplement: S10 Fig — Data were normalized with a reference gene (ACTB), reported to mock control, and expressed with a relative quantitation method (ΔΔCt). Statistical analysis. All data presented were calculated from three biological replicate (n = 3) measurements ± SD. The one-way analysis of variance (ANOVA) and Dunnett’s multiple comparisons were used for statistical analysis. Statistically significant differences (fold change vs. mock) are indicated by stars (*), * p < 0.05; ** p < 0.01; *** p < 0.001, **** p < 0.0001. (DOCX) [file ppat.1010827.s010.docx]

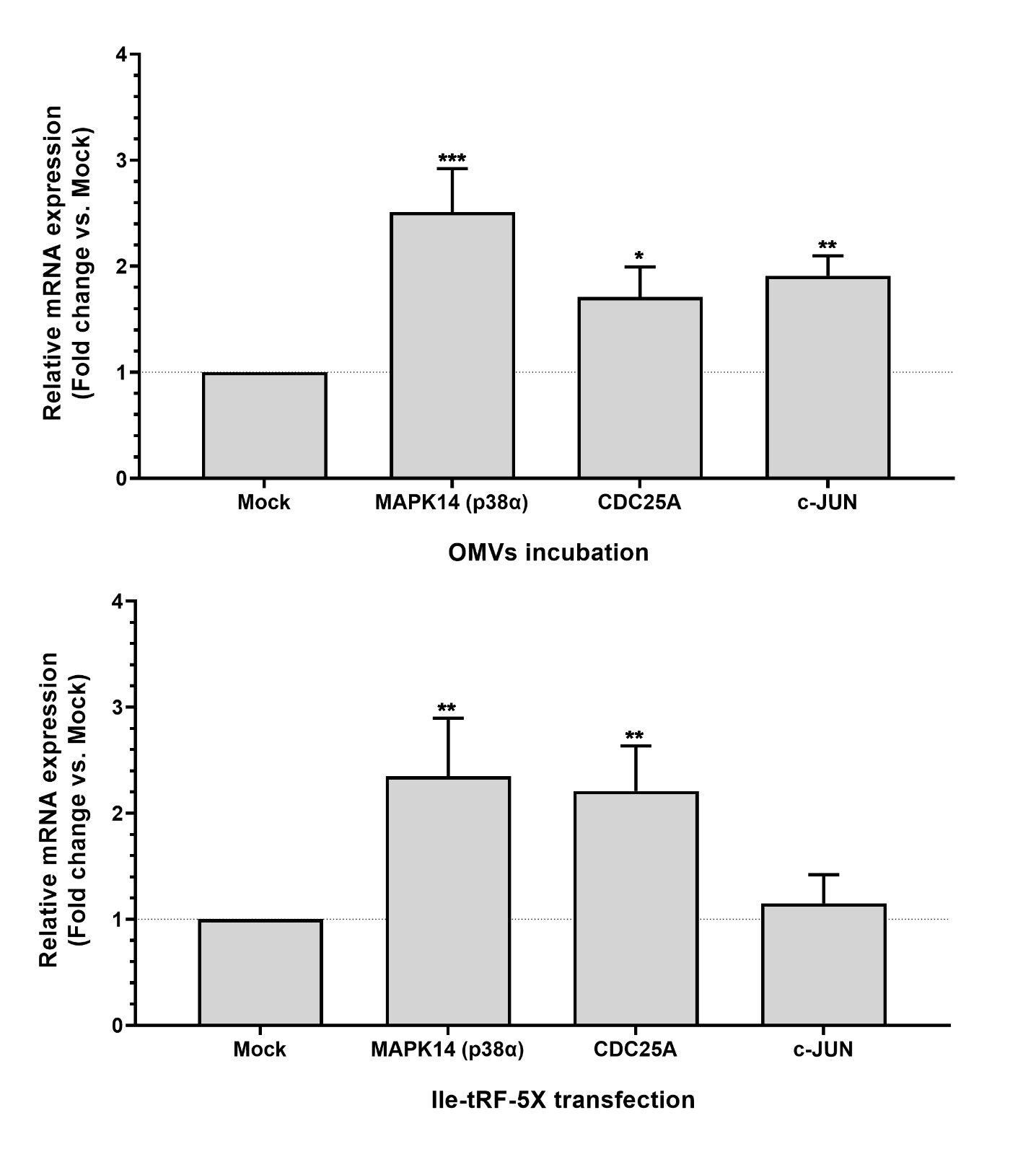


**Supplementary Figure S10. Relative quantification of MAPK14 (p38α), CDC25A and c-JUN expressions at mRNA level in HCT116 cells by RT-qPCR after incubation with OMVs or transfection with Ile-tRF-5X**. Data were normalized with a reference gene (ACTB), reported to mock control, and expressed with a relative quantitation method (ΔΔCt). **Statistical analysis**. All data presented were calculated from three biological replicate (n = 3) measurements ± SD. The one-way analysis of variance (ANOVA) and Dunnett’s multiple comparisons were used for statistical analysis. Statistically significant differences (fold change vs. mock) are indicated by stars (*), * p < 0.05; ** p < 0.01; *** p < 0.001, **** p < 0.0001.
