# Supplementary material for: A tRNA-derived fragment present in E. coli OMVs regulates host cell gene expression and proliferation
Source: PLoS Pathog. 2022 Sep 15;18(9):e1010827. doi: 10.1371/journal.ppat.1010827 (PMC9514646; doi:10.1371/journal.ppat.1010827)
Supplement: S11 Fig — Relative mRNA expression was quantified by RT-qPCR. Data were normalized with a reference gene (ACTB), reported as fold change vs mock control, and expressed with the relative quantitation method (ΔΔCt). Statistical analysis. Data were calculated from three biological replicate measurements (n = 3; mean ± SD), and each sample was tested in triplicate. Two-way analysis of variance (ANOVA) and Holm-Šídák’s multiple comparisons test (post-hoc test) were used for statistical analysis. Statistically significant differences (fold change vs mock) are indicated as follows: * p < 0.05. (DOCX) [file ppat.1010827.s011.docx]

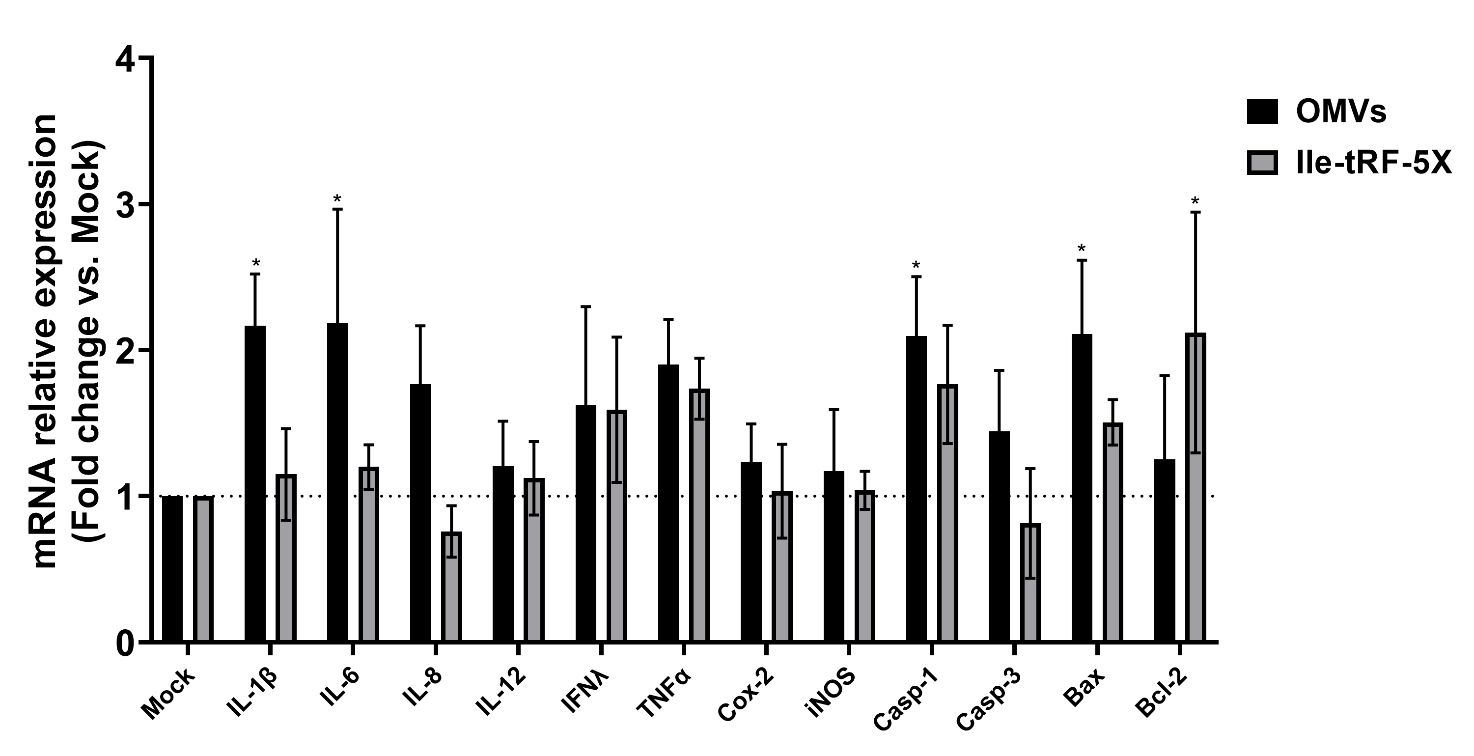


**Supplementary Figure S11. Changes in expression of cytokines and apoptosis factors in HCT116 cells after incubation with bacterial OMVs or transfection with Ile-tRF-5X.** Relative mRNA expression was quantified by RT-qPCR. Data were normalized with a reference gene (ACTB), reported as fold change vs mock control, and expressed with the relative quantitation method (ΔΔCt). **Statistical analysis**. Data were calculated from three biological replicate measurements (n=3; mean ± SD), and each sample was tested in triplicate. Two-way analysis of variance (ANOVA) and Holm-Šídák's multiple comparisons test (post-hoc test) were used for statistical analysis. Statistically significant differences (fold change vs mock) are indicated as follows: * p < 0.05.
