# Supplementary material for: A tRNA-derived fragment present in E. coli OMVs regulates host cell gene expression and proliferation
Source: PLoS Pathog. 2022 Sep 15;18(9):e1010827. doi: 10.1371/journal.ppat.1010827 (PMC9514646; doi:10.1371/journal.ppat.1010827)
Supplement: S12 Fig — A) Experimental scheme for the detection of vsRNAs, here Ile-tRF-5X as example (See mat. & meth. section). B) specificity test of Exiqon Ile-tRF-5X primers. The primers can discriminate Ile-tRF-5X from any other sequence by up to one nucleotide difference using LNA technology. C) Single peak melt curve assessing the specificity of amplification. Ile-tRF-5X melt curve analysis shows the production of specific and single product. D) comparative analysis of Ile-tRF-5X quantifications by RNA-seq (semi-quantitative) and by qPCR reported in percentage. Ile-tRF-5X was quantified in E. coli exponential growth phase (reference = R), stationary phase, after treatment with rifampicin (R+rif) or chloramphenicol (R+cat), or finally in derived OMVs. (DOCX) [file ppat.1010827.s012.docx]

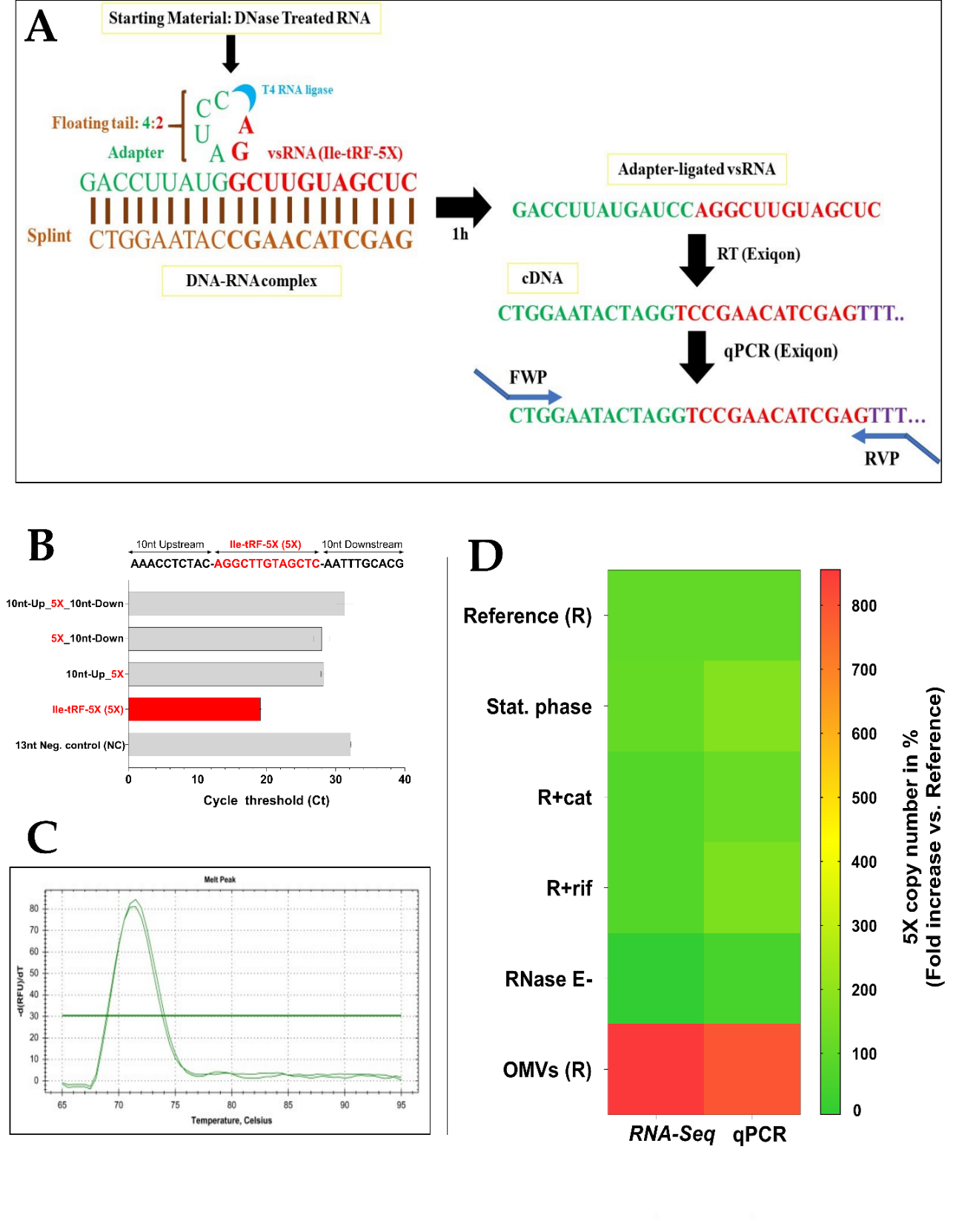


**Supplementary Figure S12. vsRNA monitoring (Splint-ligation-based strategy).** A) Experimental scheme for the detection of vsRNAs, here Ile-tRF-5X as example (See mat. & meth. section). B) specificity test of Exiqon Ile-tRF-5X primers. The primers can discriminate Ile-tRF-5X from any other sequence by up to one nucleotide difference using LNA technology. C) Single peak melt curve assessing the specificity of amplification. Ile-tRF-5X melt curve analysis shows the production of specific and single product. D) comparative analysis of Ile-tRF-5X quantifications by RNA-seq (semi-quantitative) and by qPCR reported in percentage. Ile-tRF-5X was quantified in *E. coli* exponential growth phase (reference = R), stationary phase, after treatment with rifampicin (R+rif) or chloramphenicol (R+cat), or finally in derived OMVs.
