# Supplementary material for: A tRNA-derived fragment present in E. coli OMVs regulates host cell gene expression and proliferation
Source: PLoS Pathog. 2022 Sep 15;18(9):e1010827. doi: 10.1371/journal.ppat.1010827 (PMC9514646; doi:10.1371/journal.ppat.1010827)
Supplement: S1 Table — (DOCX) [file ppat.1010827.s013.docx]

**Supplementary Table S1. List of bacteria used in the study and their growth conditions.**

| N* | Bacteria | Background | Media | Growth phase | Inoculum | OD_600nm_ | Incubation | Conditions | Stress |
| --- | --- | --- | --- | --- | --- | --- | --- | --- | --- |
| 1 | *E*. coli | K12 MG1655 | LB | Exponential | ON culture  (1/1000 in 50 ml) | 0.449 | 2h45 | Growth at 37°C | Reference |
| 2 |  |  |  | Stationary |  | 2.23 | 5h |  | Stationary phase |
| 3 |  |  |  | Exponential |  | 0.429 | 4h15 | Growth at 30°C | Temperature |
| 4 |  |  |  |  |  | 0.445 | 2h45 | Growth at 44°C |  |
| 5 |  |  | M63 glucose |  | ON culture  (1/100 in 50 ml) | 0.349 | 4h45 | Growth at 37°C | Nutrients |
| 6 |  |  |  | Stationary |  | 2 | 7h30 |  |  |
| 7 |  |  | LB | Exponential | ON culture  (1/1000 in 50 ml) | 0.453 | 2h45 | Growth at 37°C  100 ng/µl chloramphenicol | Protein synthesis - |
| 8 |  |  |  |  |  | 0.449 | 2h45 | Growth at 37°C  500 ng/µl rifampicin | RNA synthesis - |
| 9 |  | *rne*-3071 ts  (EM1277) |  |  |  | 0.411 | 4h15 | Heat shock (30 to 44°C) | RNase E - |
| 10 |  | *rnpA* ts (KP1036) |  |  |  | 0.421 | 8h25 |  | RNase P - |

ON = overnight.
