# Supplementary material for: A tRNA-derived fragment present in E. coli OMVs regulates host cell gene expression and proliferation
Source: PLoS Pathog. 2022 Sep 15;18(9):e1010827. doi: 10.1371/journal.ppat.1010827 (PMC9514646; doi:10.1371/journal.ppat.1010827)
Supplement: S2 Table — (DOCX) [file ppat.1010827.s014.docx]

**Supplementary Table S2. Primers used in the study.**

| Transcript names | 5’🡪 3’ sequences (FW/RV) | Accession number |
| --- | --- | --- |
| MAP3K4 | TACAAGACCCAGGTGGGCG  ACAGGACTGTGTGGTTTTCCT | NM_001291958.2 |
| MAPK14 | TGGATGCATTACAACCAGACAGT  AGACTGAATATAGTTTCTTGCCTCA | NM_001315.3 |
| MAP2K6 | TGCAGCTTGCATCTTTGTTGC  AATCTCGAGGTGGTGTGGAAC | NM_001330450.2 |
| GAPDH | GGATTTGGTCGTATTGGG  CTCGCTCCTGGAAGATGG | NM_001256799.3 |
| CDC25A | CCTACCTCAGAAGCTGTTGGGA  GCAGAGTTCTGCCTCTGTGT | NM_001789.3 |
| cJUN | TTCTATGACGATGCCCTCAACGC  GCTCTGTTTCAGGATCTTGGGGTTAC | NM_002228.4 |
| ACTB | GATTCCTATGTGGGCGACGA  AGGTCTCAAACATGATCTGGGT | NM_001101.5 |
| 23S rRNA | GTTAAGCGACTAAGCGTACAC  QIAGEN Universal reverse primer | EG30078 (EcoCyc) |
| tRNA-Ile | CTTGTAGCTCAGGTGGTTAGAG  QIAGEN Universal reverse primer | EG30043 (EcoCyc) |
| 16S rRNA | TCTCTGAGATGTTCGCAAGC  QIAGEN Universal reverse primer | EG30089 (EcoCyc) |
| Mutated Ile-tRF | AAACUCGUGGCCU | Designed for the project and ordered from IDT |
| Antisens Ile-tRF-5X | GAGCUACAAGCCU | Designed for the project and ordered from IDT |

Primers were chosen to provide specific amplification of the target messengers (span exon-exon junction) and different pre-tests were carried out to determine the best annealing temperatures associated with each oligo pair.
