# Supplementary material for: A tRNA-derived fragment present in E. coli OMVs regulates host cell gene expression and proliferation
Source: PLoS Pathog. 2022 Sep 15;18(9):e1010827. doi: 10.1371/journal.ppat.1010827 (PMC9514646; doi:10.1371/journal.ppat.1010827)
Supplement: S1 Information — (DOCX) [file ppat.1010827.s015.docx]

**S1 Information. Details of the design of the plasmid construction for the dual luciferase assay with MAP3K4 3'UTR WT (A) and MAP3K4 3'UTR MUT (B)**

1. **Wild-type (WT) MAP3K4 3’-UTR**

*TAAGCA*CTCGAGAGCCTAGTAGAATATGGACTTGGAAAATTCTCTTAATC**ACTACTGTA**TGTAATATTTACATAAAGACTGTGCTG**AGA~~AGC~~A**G**~~TATAAGCCT~~**TTTTAACCTTCCAAGACTGAA**GACTGCA**CA**~~GGT~~**G**~~ACAAGC~~**GTCACTTCTCC**TGCTGCT**CCTGTTTGTCTGATGTGGCAAAAGGCCCTCTGGA**~~GGGCT~~G**GT**~~GGCC~~**ACGAGGTTAAAGA**~~AGCTGCA~~**TGTTA**AGT~~GCC~~A**TT**ACTACTGTA**CACGGACCATCGCCTCTGTCTCCTCCGTGTCTCGCGCGACTGAGAACCGTGACATCAGCGTAGTGTTTTGACCTTTCTAGGTTCAAAAGAAGTTGTAGTGTTATCAGGCGTCCCATACCTTGTTTTTAATCTCCTGTTTGTTGAG**TGCACTGACTGTGAA**ACCTTTACCTTTTTTGTTGTTGTTGGCA**~~AGCTGCAGGTTT~~**GTAATGCAAAAGGCTGATTACTGAAATTTAAGAAAAAGGTTCTTTTTTCAATAAATGGTTTATTTTAGGAAAGCTCAGCGGCCGCATTCGT


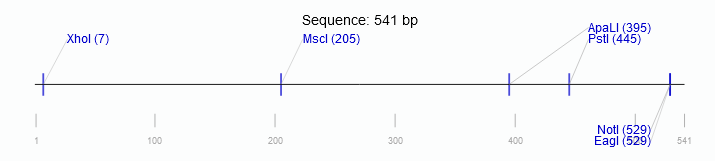


1. **Mutated (MUT) MAP3K4 3’-UTR**

*TAAGCA*CTCGAGAGCCTAGTAGAATATGGACTTGGAAAATTCTCTTAATC**ACTACTGTA**TGTAATATTTACATAAAGACTGTGCTG**AGA~~AGC~~A**G**~~GAGACGACG~~**TTTTAACCTTCCAAGACTGAA**GACTGCA**CA**~~TGG~~**G**~~CCCATC~~**GTCACTTCTCC**TGCTGCT**CCTGTTTGTCTGATGTGGCAAAAGGCCCTCTGGA**~~GTGAT~~T**GT**~~GTCA~~**ACGAGGTTAAAGA**~~ATCGGAA~~**TGTTA**AGT~~GCC~~A**TT**ACTACTGTA**CACGGACCATCGCCTCTGTCTCCTCCGTGTCTCGCGCGACTGAGAACCGTGACATCAGCGTAGTGTTTTGACCTTTCTAGGTTCAAAAGAAGTTGTAGTGTTATCAGGCGTCC**C**ATACCTTGTTTTTAATCTCCTGTTTGTTGAG**TGCACTGACTGTGAA**ACCTTTACCTTTTTTGTTGTTGTTGGCA**~~ATCGGAATGGTG~~**GTAATGCAAAAGGCTGATTACTGAAATTTAAGAAAAAGGTTCTTTTTTCAATAAATGGTTTATTTTAGGAAAGCTCAGCGGCCGCATTCGT

**XX : Restriction site XhoI & NotI, no MUT + some random extra nucleotides**

**~~XX~~ : Ile-tRF-5X binding sites(~~Barred~~ + Italic = seed seq : RNA22 + RNhybrid)**

**~~XX~~ : Mutated Ile-tRF-5X binding sites(~~Barred~~ + Italic = seed sequence : RNA22 + RNAhybrid)**

**XX : miR-199-3p;101-3p.2; miR-144-3p**

**XX : miR-455-3p.2**

**XX : miR-497-5p; miR-195-5p; miR-15b-5p; miR-15a-5p; miR-16-5p**

**XX : miR-183-5p**

**XX : miR-148-3p; miR-152-3p (higher probab. of conserve. Site)**

**XX : miR-103a-5p**
