# Supplementary material for: A tRNA-derived fragment present in E. coli OMVs regulates host cell gene expression and proliferation
Source: PLoS Pathog. 2022 Sep 15;18(9):e1010827. doi: 10.1371/journal.ppat.1010827 (PMC9514646; doi:10.1371/journal.ppat.1010827)
Supplement: Attachment — Submitted filename: Point-by-point response to the Reviewers_MS#2_5X.docx [file ppat.1010827.s016.docx]

**Point-by-point response to the Reviewers**

**Manuscript title:** A tRNA-derived fragment present in E. coli OMVs regulates host cell gene expression and proliferation"

**Manuscript ID:** ?

**Authors:** Idrissa Diallo, Jeffrey Ho, Marine Lambert, Abderrahim Benmoussa, Zeinab Husseini, David Lalaouna, Eric Massé & Patrick Provost1*

**Journal:** PLOS Pathogens

**Article type:** Original Research

**Submitted on:** April 09, 2022

*** Corresponding author:**	Dr. Patrick Provost

CHUQ Research Center/CHUL Pavilion

2705 Blvd Laurier, Room T1-65

Quebec, QC G1V 4G2

Canada

Phone: 1 418 525 4444 (ext. 48842)

E-mail: [patrick.provost@crchudequebec.ulaval.ca](mailto:patrick.provost@crchudequebec.ulaval.ca)

**Editor’s response to the Authors**

Dear Dr. Provost,

Thank you very much for submitting your manuscript "A tRNA-derived fragment present in E. coli OMVs regulates host cell gene expression and proliferation" for consideration at PLOS Pathogens. As with all papers reviewed by the journal, your manuscript was reviewed by members of the editorial board and by several independent reviewers. In light of the reviews (below this email), we would like to invite the resubmission of a significantly-revised version that takes into account the reviewers' comments. Both reviewers agreed that the work is interesting, well done and of general interest to the broader scientific community working on regulatory RNAs and host-pathogen interactions. However, there are several important points that the authors have to address. Among them, the previous results must be accessible to reviewers AND readers. I recommend that the authors publish the submitted work (Diallo et al.,manuscript submitted) on a preprint repository such as bioRxiv. If this is not possible, authors should present the previous data in their rebuttal letter. However, I do not encourage the latter as this present work should not be published without the publication of the previous work. We cannot make any decision about publication until we have seen the revised manuscript and your response to the reviewers' comments. Your revised manuscript is also likely to be sent to reviewers for further evaluation.

When you are ready to resubmit, please upload the following:

[1] A letter containing a detailed list of your responses to the review comments and a description of the changes you have made in the manuscript. Please note while forming your response, if your article is accepted, you may have the opportunity to make the peer review history publicly available. The record will include editor decision letters (with reviews) and your responses to reviewer comments. If eligible, we will contact you to opt in or out.

[2] Two versions of the revised manuscript: one with either highlights or tracked changes denoting where the text has been changed; the other a clean version (uploaded as the manuscript file).

Important additional instructions are given below your reviewer comments.

Please prepare and submit your revised manuscript within 60 days. If you anticipate any delay, please let us know the expected resubmission date by replying to this email. Please note that revised manuscripts received after the 60-day due date may require evaluation and peer review similar to newly submitted manuscripts.

Thank you again for your submission. We hope that our editorial process has been constructive so far, and we welcome your feedback at any time. Please don't hesitate to contact us if you have any questions or comments.

Sincerely,

Mathieu Coureuil
Guest Editor
PLOS Pathogens

Xavier Nassif
Section Editor
PLOS Pathogens

Kasturi Haldar
Editor-in-Chief
PLOS Pathogens
orcid.org/0000-0001-5065-158X

Michael Malim
Editor-in-Chief
PLOS Pathogens
orcid.org/0000-0002-7699-2064

**Response of the Authors**

We wish, if the revisions are accepted, that our exchanges with the reviewers are published. We wish to thank you for the processing of our manuscript and hope the attached revised version of our review manuscript is now suitable for publication in PLOS PATHOGENS

**Reviewers’ comments**

**Reviewer #1**

In this manuscript, **Diallo et al** describe the regulatory impact of an abundant short RNA from outer membrane vesicles (OMVs) of Escherichia coli, isoleucine tRNA-derived fragment Ile-tRNA-5X, on the components of mitogen-activated protein kinase pathway in human host cells.
The authors emphasize an important point on the RNA-based crosstalk during host-pathogen interactions and the role of tRNA-fragments in these cross-regulations.
They monitored the amount of this particular 13-nt in length tRNA-fragment and showed that it is modulated by environmental stresses (nutritional and thermal stress signals) and is delivered through OMVs to human cells in culture promoting host cell gene expression and proliferation. The authors also started to explore the molecular mechanisms of these regulatory effects and suggested the competition with gene silencing properties of miRNA.
Overall, the paper is well written, and the experiments are well performed. However, the work is rather descriptive and largely based on another submitted paper of the same team. Novelty of the present work as compared to previous studies implicating tRNA-fragments in MAPK-signalling pathways should be emphasized.
I have several concerns that should be addressed prior to publication.

**Reviewer #1, comment 1:**

Previous results are cited several times in the manuscript as (Diallo et al submitted), however, the submitted paper is not available. It is not easy to follow the current manuscript without these data. For example, page 5 « are predicted to target several host mRNAs with diverse function (Diallo et al, submitted) », please provide details. Page 12, “several tRNAs might be targeted by tRF vsRNAs (Diallo et al, submitted)”.
I wondered if authors considered the BioRxiv submission of the cited paper?

**Response of the Authors:**

Indeed, this present manuscript, proof of concept of the functionality of small RNAs below 16 nt, is a continuation of the story we published on April 07, 2022, in the Journal Frontiers in Molecular Biosciences (entitled “[RNA sequencing unveils very small RNAs with potential regulatory functions in bacteria](https://www.frontiersin.org/articles/10.3389/fmolb.2022.914991/full?&utm_source=Email_to_authors_&utm_medium=Email&utm_content=T1_11.5e1_author&utm_campaign=Email_publication&field=&journalName=Frontiers_in_Molecular_Biosciences&id=914991)”; accepted on May 2, 2022).

We absolutely understand the necessity of having the previous manuscript available because it explains some of our choices for the experimental strategy and especially where some preliminary data (RNA-Seq) come from.

**Abstract:**

RNA sequencing (RNA-seq) is the gold standard for the discovery of small non-coding RNAs. Following a long-standing approach, reads shorter than 16 nucleotides (nt) are removed from the small RNA sequencing libraries or datasets. The serendipitous discovery of an eukaryotic 12 nt-long RNA species capable of modulating the microRNA from which they derive prompted us to challenge this dogma and, by expanding the window of RNA sizes down to 8 nt, to confirm the existence of functional very small RNAs (vsRNAs <16 nt). Here we report the detailed profiling of vsRNAs in Escherichia coli, E. coli-derived outer membrane vesicles (OMVs) and five other bacterial strains (Pseudomonas aeruginosa PA7, P. aeruginosa PAO1, Salmonella enterica serovar Typhimurium 14028S, Legionella pneumophila JR32 Philadelphia-1 and Staphylococcus aureus HG001). vsRNAs of 8 to 15 nt in length (RNAs(8-15nt)) were found to be more abundant than RNAs of 16 to 30 nt in length (RNAs(16-30nt)). vsRNA biotypes were distinct and varied within and across bacterial species and accounted for one third of reads identified in the 8 to 30 nt window. The tRNA-derived fragments (tRFs) have appeared as a major biotype among the vsRNAs, notably Ile-tRF and Ala-tRF, and were selectively loaded in OMVs. tRF-derived vsRNAs appear to be thermodynamically stable with at least 2 G-C basepairs and stem-loop structure. The analyzed tRF-derived vsRNAs are predicted to target several human host mRNAs with diverse functions. Bacterial vsRNAs and OMV-derived vsRNAs could be novel players likely modulating the intricate relationship between pathogens and their hosts.

<https://www.frontiersin.org/articles/10.3389/fmolb.2022.914991/full>

**Reviewer #1, comment 2:**

The generation of tRNA-derived fragments has been reported to impact cell proliferation through MAPK-signalling pathway modulation in previous work on OMV-associated short RNAs. The novelty of the present study should be emphasized as compared to previously reported data.

**Response of the Authors:**

In fact, we mention in an honest, explicit, and complete way to the best of our knowledge the previous studies that have been published on this topic.

We can state that there are very few publications on bacterial tRFs and sometimes when they are reported the classification/nomenclature does not always follow the one already established for eukaryotic tRFs.

To the best of our knowledge, we dated the first detailed characterization E. coli RNA OMVs in 2015 with reference to the abundance of tRNA fragment (over 90% of sequence reads were mapped to tRNA fragments) [1] and the first example of trans-kingdom biological activity of a regulatory tRF contained in OMVs in 2016 [2]. In the same period, other studies have documented the transfer and delivery of OMVs RNA content (no tRF reported) into host cells but without mentioning precise roles and/or mechanism of action of these RNAs [3,4]. Recently, Mu et al., [5] reported proliferation of colorectal cancer cells via the MAPK/ERK Signaling Pathway induced by intracellular P. gingivalis. They did not specify the mechanistic pathway involving RNAs or more precisely tRFs.

Without mentioning proliferation, two other studies reported the transfer of functional tRNA fragments to the host with a modulatory effect on the immune system but they did not detail the mechanisms of action [6,7].

Koppen et al.,[2] showed that tRF (24 nt, tRNA-Met) that is predicted to target kinases in the LPS-stimulated MAPK signaling pathways, reduces the LPS-mediated induction of IL-8 mRNA and protein secretion in primary human airway epithelial cells and keratinocyte-derived chemokine production in mouse lungs. The tRF was predicted to downregulate five kinases in the LPS-stimulated MAPK pathway and two of these kinases (MAP3K7 and MAP2K4) were significantly reduced.

Our study is the very first in E. coli to report the existence of abundant tRF, found in vesicles (OMVs) and assuming potential functions in the host. It is also the first functional 13 nt tRF reported in bacteria.

Following a long-standing approach, reads shorter than 16 nt (vsRNA) are removed from the small RNA sequencing libraries or datasets; in the face of this fact, it is important for us to clarify that the first motivation of our study is the reconsideration of the biological importance of vsRNAs and thus a call for a revision of scientific concepts that exclude small RNAs because of their small size. Based on this approach (RNA-Seq without ribodepletion, in the 8-30nt window), we have characterized new functional RNA types with unusual sizes [8–10]. Further, the role of RNAse E in tRNA maturation is well known [11–13] but until now its potential contribution to tRF generation has never been documented and our study provides a new insight in this direction.

**Reviewer #1, comment 3:**

I wondered how the bacterial origin of the tRNA fragments could be proven? Are there similar fragments in human cells? Please consider this point.

**Response of the Authors:**

It is known that tRNA represents the most abundant biotype of total RNAs [14–16]. tRNA fragments account for the majority of reads found in the RNA-Seq data. From this, it is highly likely that a random sequence belongs to the tRNA biotype.

But beyond the probabilities, we have sequenced total RNA of bacteria from which we have removed for E. coli the contaminant sequences from the LB culture medium (mainly s. cerevisiae).

For this reason, we are confident that the sequences we have obtained are of bacterial origin. By an alignment on both the [NCBI](https://blast.ncbi.nlm.nih.gov/Blast.cgi) and the [GtRNAdb](http://gtrnadb.ucsc.edu/) databases, the correspondence is perfect and unambiguous. In addition, the raw data has been aligned with Novoalign (REF MS#1) which showed higher sensitivity towards short reads [17,18].

We have, in collaboration with EXIQON experts ([later acquired by Qiagen](https://corporate.qiagen.com/newsroom/press-releases/press-release-details/2016/QIAGEN-announces-successful-completion-of-tender-offer-for-shares-in-Exiqon/default.aspx)) designed very sensitive and specific oligos based on LNA. If the target sequence has a single extra nucleotide in its 3' or 5', the oligos are able to discriminate it and preliminary experiments allow us to establish a detection threshold that permits us to formally affirm that the amplification observed in the X or Y condition is indeed that of the target sequence.

Thereafter, the vsRNA transfection or co-incubation experiments with OMVs took place. And in this context, we did not do RNA-Seq but rather a qPCR quantification using the oligos described above.

Screen capture of the NCBI- BLASTN platform with the example of the blast of the Ile-tRF-5X sequence discussed in the study. The Ile-tRF-5X sequence is specific to bacteria possessing Ile-tRNA.

In Escherichia coli str. K-12 substr. MG1655, it originates from 3 sources of tRNA-Ile :

1_ tRNA-Ile-GAT-1-1 (tRNAscan-SE ID: chr.trna1) Ile (GAT) 77 bp mature sequence Sc: 75.8 chr:225381-225457 (+)

2_ tRNA-Ile-GAT-1-2 (tRNAscan-SE ID: chr.trna58) Ile (GAT) 77 bp mature sequence Sc: 75.8 chr:3427076-3427152 (-)

3_ tRNA-Ile-GAT-1-3 (tRNAscan-SE ID: chr.trna41) Ile (GAT) 77 bp mature sequence Sc: 75.8 chr:4037141-4037217 (+)

**Reviewer #1, comment 4:**

The level of Ile-tRF-5X should be compared with the level of precursors/mature Ile-tRNA. The normalization strategy should be specified.

**Response of the Authors:**

Since we did not know the exact mechanism of Ile-tRF-5X generation, we were unable to use the original tRNA (Ile-tRNA) as a control. However, we measured it under certain conditions as mentioned on page 8.

We mention on page 8: “A complementary RT-qPCR analysis (data not shown) showed that the level of tRNAs was affected by inhibition of RNA and protein synthesis, suggesting a lack of correlation between the level of tRNAs and that of tRFs, such as Ile-tRF-5X”. We discussed this issue on page 22.

In the experiments performed with HCT116 cells, ACTB and GAPDH were used as reference genes (internal standard) for gene monitoring such as MAP3K4. In the experiments performed only with bacteria, 23S and 16S were used as reference genes (tRNA-Ile be part of the 16S-23S rRNA ITS [19]).

In both situations UniSp6 served as a spike-in and thus as a normalizer as well. In table S2 (Document S2), we mentioned the sequences of these genes (ACTB, GAPDH, 23S, 16S) that were used for qPCR. When required, the Ile-tRF-5X is used in parallel with its negative control of the same length. Finally, the relative quantitation was calculated using the ∆∆Ct method (removing differences due to sampling and quality of the samples) [20].

We are aware of the importance of controls and standard setting, and we took the necessary precautions recommended by the literature [21]. The miRCURY LNA RT Kit used in this study include the RNA spike-in template for monitoring performance. Besides being a cDNA synthesis efficiency marker for small non coding RNA, Unisp6 is also used as endogenous control [22–24]. We believe that the use of spike-in included in the kits for cDNA synthesis is a reasonable option. Even the 16S and 23S in case of rifampicin treatment for example may be subjected to huge variations [25].

The other bacterial reference genes for gene expression studies by RT-qPCR have also not received the necessary validation or are equally subject to variations according to the treatment [26].There is no endogenous reference transcript which is known to be suitable for comparing the cellular levels of tRNA (or any other RNA species) during starvation to the levels during steady-state growth [27]. Sørensen et al., [27] propose to use a spike-in cells from a separate culture, which will be added to the experimental samples prior to RNA extraction. For the dual processing of our samples in RNA-Seq and RT-qPCR this method does not fit our primary objectives. We have clarified and detailed the information in the concerned sections to avoid any confusion.

| 23S rRNA | GTTAAGCGACTAAGCGTACAC  QIAGEN Universal reverse primer | EG30078 (EcoCyc) |
| --- | --- | --- |
| tRNA-Ile | CTTGTAGCTCAGGTGGTTAGAG  QIAGEN Universal reverse primer | EG30043 (EcoCyc) |
| 16S rRNA | TCTCTGAGATGTTCGCAAGC  QIAGEN Universal reverse primer | EG30089 (EcoCyc) |

**Extract from Table S2 (Document S2)**

**Figure S2. Bacterial Ile-tRF-5X and mature Ile-tRNA levels under different experimental conditions**. (**A**) *E. coli* MG1655 bacteria were grown at 37°C up to the exponential (Reference, R) and stationary phases of growth in either complete (rich) LB or minimal M63 medium. (**B**) *E. coli* MG1655 bacteria were grown in LB medium at exponential phase (R) in different temperatures: 30, 37 and or 44°C. (**C**) Bacterial mRNA or protein synthesis was inhibited by addition of chloramphenicol or rifampicin, respectively, to cultures of *E. coli* MG1655 grown at exponential phase (R). The level of bacterial Ile-tRF-5X level is not modulated by transcription or translation activity (**D**) *E. coli* strains carrying heat-sensitive (hs) mutations in the essential genes rne-3071-hs (EM1277) and rnpA-hs (KP1036) were grown in LB medium at 30°C and then heat-shocked (44°C) to transiently inhibit ribonuclease (RNase) P or RNase E, which are involved in tRNA maturation. Bacteria RNase E contributes to Ile-tRF-5X biogenesis. For more details, see **Supplementary Table S1**. In all conditions, the level of Ile-tRF-5X and Ile-tRNA were measured by LNA RT-qPCR. A spike-in (UniSp6) and reference genes (23S and/or 16S) were used as control and for normalization. The results are reported in fold change compared to the reference condition. **Statistical analysis**. Data were calculated from three biological replicate measurements (n=3; mean ± SD). Two-way analysis of variance (ANOVA) and Dunnett's multiple comparisons (fold change vs. reference test) or Šídák’s multiple comparisons test (Ile-tRF-5X vs. Ile-tRNA) were used for statistical analysis. Statistically significant differences (fold change vs. reference or Ile-tRF-5X vs. Ile-tRNA shown in purple) are indicated by stars (*), * p < 0.05; ** p < 0.01; *** p < 0.001; **** p < 0.0001; ns, not significant.

**Reviewer #1, comment 5:**

Page 9: Whether the effects of additional ribonucleases has been assessed to get insight into the generation of tRF?

**Response of the Authors:**

In this project we did not test the effect of other ribonucleases on bacterial tRF processing. In E. coli, a minimum of 8 to 10 enzymes with a high degree of functional overlap is involved in just converting the primary transcript to the mature tRNA molecule [28,29]. This complicates greatly complicated the efforts to elucidate the tRF generation mechanism.

We consider that there are biochemical modifications that will condition the cleavage at particular sites. tRNA processing into 13 nt tRFs seems to involve cleavage after the sequence motif “TAGC” located on the D-arm, at the beginning of the D-loop, where the 13th nt usually carried a pseudouridine (Ψ) highly conserved among species [30,31] and important to structure, stability and ribosome binding [32]. We believe that this pseudouridylation (performed by pseudouridine synthase, ΨS, here TruD) may be crucial in the generation of the Ile-tRF.

The application of aminoglycoside antibiotic neomycin (bind specifically to the 30S ribosomal subunit and inhibit translation [33]) also offers some insights (while raising new questions) into the mechanisms that support the generation of tRFs.

For unaccountable reasons, we were not able to fully answer all of our questions yet. To be continued.

**Reviewer #1, comment 6:**

Page 27: It will be interesting to explore the impact of endogenous tRF on MAPK expression and proliferation as compared to OMVs-derived tRF investigated in this study. The possible interference between endogenous tRF and OMVs-derived tRF should be considered.

**Response of the Authors:**

Indeed, it is not excluded that endogenous tRFs act on MAPK expression and proliferation. This has been reported in the literature. For instance, it is well known that the eukaryotic tRF-1001 (functionally distinct from miRNA/siRNA.) is expressed highly in a wide range of cancer cell lines and was tightly correlated with cell proliferation [34]. However, our proliferation experiments also clearly demonstrate that Ile-tRF-5X is distinctively a promoter of cell proliferation. The 13 nt control of an unrelated sequence showed no effect and served as a normalizer. And the link with MAP3K4 is also clearly established the " on-board system " experiment using dual luciferase assay. Furthermore, as the bacterial Ile-tRF-5X induces sequence-specific upregulation of MAP3K4 mRNA expression, this could be a manifestation of the non-interference with endogenous eukaryotic tRFs (which we do not exclude the possible effect on MAP3K4). It is over and beyond any endogenous effect that our observations occur.

**Minor comments**

**Reviewer #1, comment 7:**

The total number of figures is too much (15 main figures). Some of them should be combined as panels of the same figure or moved to the supplementary material.

**Response of the Authors:**

Adjusted as recommended. We have transferred some of the figures to the supplementary files.

**Reviewer #1, comment 8:**

- Figures but not their titles and legends are included in the main text, please correct.

**Response of the Authors:**

Adjusted as recommended. It was the journal's instruction that required such a configuration.

**Reviewer #1, comment 9:**

- Page 3 : « For instance » should be removed.

**Response of the Authors:**

Corrected as requested.

**Reviewer #1, comment 10:**

- Page 4. « Bacterial OMVs are produced and released in all domains of life » please check, seems contradictory « bacterial »/ “all domains of life “
- Page 4 Escherichia coli (E. coli) : « (E. coli) » to remove, please indicate the full name the first time and the « E. coli » from the second time it appears in the text.
- Page 4 : « five other bacterial strains » please specify which strains ?
- Page 4 « Gram-positive »

**Response of the Authors:**

Corrected as requested.

**Reviewer #1, comment 11:**

- Page 5 Not clear for the name of Ile-tRF-5X

**Response of the Authors:**

Reajusted as requested. We said that the tRF in this study belong to the 5’ end of the mature Isoleucine (Ile)-tRNA (so it is a class 5 tRF : tRF-5). They are mainly of three specific lengths and are therefore further divided into subclasses: tRF-5a (14–16 nts), tRF-5b (22–24 nts) and tRF-5c (28–30 nts) [35]. Our tRF-5 of interest being 13-nt long, so it was tentatively named Ile-tRF-5X.

Illustration of a mature tRNA showing the cut sites that would generate the different subclasses of tRF-5s and tRF-3s. Extract from the reference [36].

**Reviewer #1, comment 12:**

- Page 6 I wondered whether 2-fold modulation observed is sufficient to have an impact on further regulatory processes?

**Response of the Authors:**

We did not explore in this project the effect of Ile-tRF-5X at the intracellular level (this is something we are considering). We took advantage of the tools at our disposal to explore their potential functions in the host. The fold change of 2 may seem small indeed, but if it is put in perspective with respect to the number of reads or the number of copies of vsRNA, it is reasonable to consider a potential effect, in addition to the stability of the secondary structure and the preferential packaging in OMVs that theoretically guarantee a longer half-life.

**Reviewer #1, comment 13:**

- Page 8 “RT-qPCR (data not shown)”. It is important to show the data. Please specify “tRNA” in general or Ile-tRNA ?

**Response of the Authors:**

Corrected as requested. We have revised this part of the manuscript.

**Reviewer #1, comment 14:**

- Page 9: On my opinion it is difficult to conclude that “Ile-tRF-5X is a product of a specific process” without providing additional mechanistic details

**Response of the Authors:**

We have reworded the sentence. We understand this and that's why we don't assert it in a categoric way, we suggest it.

In our RNA-Seq data [37], the mRNA and rRNA fragments randomly mapped to different positions on their original genes in contrast to the bacterial tRFs fragments, which were more consistently localized to unique positions (stacked-type matching vs. ladder-type matching). These observations suggest that bacterial tRFs may originate from specific processing events rather than random degradation. Our results in the present study show that Ile-tRF-5X is specifically modulated, sometimes irrespectively of its tRNA source.

The genetic material encoding tRNA is most frequently found in clusters on the chromosome and the presence of multiple gene copies for a single species is common [38]. This configuration, as discussed in our article, makes complex the experiments of genetic manipulation aiming at deleting/mutating/ overexpressing the tRF for functional studies.

But through the different experimental conditions tested here, and by combining our observations on this article and the previous one [37], we are able to make this suggestion.

To complete with the answer of the comment #5 (**Reviewer #1, comment 5**)

**Reviewer #1, comment 15:**

- Page 10: The choice of cell line/markers should be explained.

**Response of the Authors:**

We address this issue in the first paragraph of the discussion but also in the introduction. It is the preferential storage of some vsRNAs of interest in OMVs that led us to further consider functions in the host-pathogen relationship. We assume that the RNA of extracellular vesicles may have the potential to shape microbial communities and host–microbe interactions [39]. We investigated the dynamics of possible interactions between bacterial vsRNAs (of the tRF family) and human cells on large exchange surfaces such as the colon, which has a bacterial load (10^11^/ml) exceeding by at least two orders of magnitude all other organs [40]. To simulate the intestinal microbiota environment, we chose the intestinal bacterium E. coli, which remains the most studied organism in modern biology [41], and the colonic epithelial HCT116 cell line as human host cells [42].

**Reviewer #1, comment 16:**

- Page 11: The differences in the delivery for OMVs-associated and synthetic Ile-tRF-5X could affect the observations, please emphasize this point.

**Response of the Authors:**

Absolutely. We mentioned (p28) that the approach had some limitations: “*The Ile-tRF-5X transfection and OMV incubation approaches that we have used with human host cells have their own limitations…*[…]”

Adjusted as requested (discussion section).

**Reviewer #1, comment 17:**

- Figure 10B: Significant effect of synthetic Ile-tRF-5X in Dicer-deleted background?

**Response of the Authors:**

When comparing the effect of Ile-tRF-5X in the absence or presence of Dicer, it was statistically significant. But when comparing the effect of 5X in the Dicer -/- condition it is not very significant, hence our proposal of vsRNAs as modulators of gene expression regulation by site competition rather than as direct regulatory functions. At this stage, we do not understand all of the functionality of vsRNAs and aim, according to the availability of funding, to go in depth.

**Reviewer #1, comment 18:**

- Page 19/25: “Dicer-derived miRNA” please specify which miRNA?

**Response of the Authors:**

It is not technically possible to specify the miRNAs involved in the context of this experiment. In the current dogmatic model of canonical miRNA pathway, deletion of Dicer results in 96% loss of miRNAs [43]. There are an anecdotal number of miRNAs that are known to be Dicer-independent but are not involved in MAP3K4 regulation nor produced by the cell line used here (e.g. miR-451[44]).

The cooperation of miRNAs and their synergy [45] also makes useless any specific interaction experiment between ONE vsRNA and ONE miRNAs since in vivo there will certainly be multiple actors involved.

**Reviewer #1, comment 19:**

- Page 26: “contribute to stabilize” please check “contribute to stabilization”, however, the stability of MAP3K4 has not been assessed.

**Response of the Authors:**

Corrected as requested. The abundance of the MAP3K4 transcript (quantified by qPCR) without affecting the production of the MAP3K4 protein (quantified by WB) could be due to the stabilization of the transcript initiated by the vsRNAs binding to it. Moreover, the binding of vsRNAs on MAP3K4 could conceivably affect MAP3K4 conformation and thus prevent its cleavage by ribonucleases. We approached the stabilization of MAP3K4 in this context.

**Reviewer #1, comment 20:**

- Figure 15: This is a general figure more suitable for a review article. It should be removed or replaced by a schematic view of the model for Ile-tRF-5X effects.

**Response of the Authors:**

We understand that this figure is rather general and is more suitable for a journal article. By the way, we published a similar figure in a journal in the prelude to this project. The difference here is that we have more information about what could happen and the actors potentially involved.

However, to the extent that we say this : *“As an illustrative overview, we propose a schematic diagram representing how bacterial OMVs and their RNA content may be involved in the preservation of the delicate, dynamic and complex balance of homeostasis”*

We believe that this figure, insofar as we have reduced some of them, can remain in the publication and offer a stimulating perspective to the host-pathogen relationship rarely envisioned from the perspective of small RNAs. Furthermore, it does not over-interpret or mislead the reader about our results. Our ambition is to improve it as the results come in and to move from a more general mode to a more specific and detailed figure from a mechanical point of view.

You be the judge.

**Reviewer #1, comment 21:**

- Page 34 “strategy)” bracket to remove

**Response of the Authors:**

Corrected as requested.

**Reviewer #1, comment 22:**

- Page 36/38 GEO accession number to specify

**Response of the Authors:**

Corrected as requested. The datasets presented in this study can be found in online repositories: GEO accession: **GSE200758**, Bioproject accession: **PRJNA826503**. Information can also be found in our previous report [37].

**Reviewer #1, comment 23:**

- Page 38 “Pseudomonas strains” Please check. It is E. coli that was used in this paper if I am right.

**Response of the Authors:**

Corrected as requested. This is indeed a mistake. The strains of pseudomonas we refer to were mentioned in the previous manuscript ([37] where we sequenced 6 strains including the two pseudomonas) and did not need to be included here.

**Reviewer #1, comment 24:**

- Page 43 References: the species names should be in italic

**Response of the Authors:**

Corrected as requested in the whole list of references. The Zotero software seems to resume the information without formatting.

**Reviewer #1, comment 25:**

- Figure S3. Only one biological replicate is presented, please check?

**Response of the Authors:**

Here is how the starting material was prepared for these data (Figure S3). For each biological condition, an RNA sample was prepared by pooling equivalent amounts of total RNA isolated from 2 or 3 biological replicate samples. In order to obtain the most representative data, we opted for a pooling strategy so to minimize the influence of interindividual variability. Previous studies have shown it to be a valid alternative to biological replicates at much reduced cost for large-scale gene expression approaches [46–48].

**We wish to thank Reviewer #1 for his/her assessment of our manuscript and hope that the revised version of our manuscript is now suitable for publication in *PLOS PATHOGENS.***

**Reviewer #2**

Reviewer #2: Diallo and colleagues have investigated the role of a very small bacterial RNA (vsRNA) produced by the commensal bacterium E. coli in the context of bacteria- host interaction. This vsRNA named Ile-tRF-5X originates from the fragmentation of a tRNA, Ile-tRNA, and is transported by OMVs produced by E. coli MG1655. Specifically, they have shown that Ile-tRF-5X production can be modulated by bacterial growth conditions but not by inhibition of transcription and translation and that RNaseE is involved in its production. Ile-tRF-5X can be transferred via OMVs to HCT116 human cells where it remains relatively stable. The abundance of MAP3K4 mRNA that contain several Ile-tRF-5X binding sites is induced by Ile-tRF-5X, while OMV that contain Ile-tRF-5X also induce MAP3K4 at the protein level. The authors also evaluated the impact of the miRNA pathway on the Ile-tRF-5X-dependent modulation of MAP3K4 expression to decipher the underlying regulatory mechanism. Finally, the impact of OMV and Ile-tRF-5X on HCT116 cell proliferation and expression of various genes involved in inflammation and apoptosis have been investigated. Since the role of OMV and its RNA cargo in mediating host microbiota interactions is a relatively new area, this manuscript makes a meaningful contribution to the field that is likely to be of broad interest. Overall, the manuscript is well written, the experiments sound, and the conclusions reasonable. However, there are some areas where the manuscript should be improved before publication, including additional controls in experiments.

**Reviewer #2, comment 1:**

In many place in the MS, the authors refer to data obtained in a previous work and submitted recently to justify some experiments and/or to strengthen their results. For example:
P5: “The loading of E. coli vsRNAs, especially tRFs, in OMVs seemed to be selective. We also observed that tRFs are probably produced upon specific processing of tRNA species, form thermodynamically stable hairpin structures, and are predicted to target several host mRNAs with diverse functions (Diallo et al., manuscript submitted).”
P6: “In a rich and complete LB medium, E. coli Ile-tRF-5X level
seemed not to be affected by the growth phase, as suggested by our RNA-Seq data (see
Supplementary Figure S3). »
P8:” These RT-qPCR results confirm our RNA-Seq data suggesting that the level of bacterial Ile-tRF-5X is not affected by inhibition of transcription or translation (Supplementary Figure S3). »
P10:” We have previously shown that a group of thermodynamically stable bacterial vsRNAs, including Ile-tRF-5X (Figure 1), were selectively enriched and loaded into OMVs (Diallo et al., manuscript submitted; Supplementary Figure S4), suggesting a potential role in bacteria-host cell communications. “
It is difficult for the reviewer to deal with this information provided by the authors as a fact while it has not been still reviewed elsewhere. At times, the results also appear as validations of previous results to which we do not yet have access.

**Response of the Authors:**

See response to **Reviewer #1, comment 1:**

Indeed, this present manuscript, proof of concept of the functionality of small RNAs below 16 nt, is a continuation of the story we published on April 07, 2022, in the Journal Frontiers in Molecular Biosciences (entitled “[RNA sequencing unveils very small RNAs with potential regulatory functions in bacteria](https://www.frontiersin.org/articles/10.3389/fmolb.2022.914991/full?&utm_source=Email_to_authors_&utm_medium=Email&utm_content=T1_11.5e1_author&utm_campaign=Email_publication&field=&journalName=Frontiers_in_Molecular_Biosciences&id=914991)”; accepted on May 02, 2022, published on June 03, 2022).

We absolutely understand the necessity of having the previous manuscript available because it explains some of our choices for the experimental strategy and especially where some preliminary data (RNA-Seq) come from.

**Abstract:**

RNA sequencing (RNA-seq) is the gold standard for the discovery of small non-coding RNAs. Following a long-standing approach, reads shorter than 16 nucleotides (nt) are removed from the small RNA sequencing libraries or datasets. The serendipitous discovery of an eukaryotic 12 nt-long RNA species capable of modulating the microRNA from which they derive prompted us to challenge this dogma and, by expanding the window of RNA sizes down to 8 nt, to confirm the existence of functional very small RNAs (vsRNAs <16 nt). Here we report the detailed profiling of vsRNAs in Escherichia coli, E. coli-derived outer membrane vesicles (OMVs) and five other bacterial strains (Pseudomonas aeruginosa PA7, P. aeruginosa PAO1, Salmonella enterica serovar Typhimurium 14028S, Legionella pneumophila JR32 Philadelphia-1 and Staphylococcus aureus HG001). vsRNAs of 8 to 15 nt in length (RNAs(8-15nt)) were found to be more abundant than RNAs of 16 to 30 nt in length (RNAs(16-30nt)). vsRNA biotypes were distinct and varied within and across bacterial species and accounted for one third of reads identified in the 8 to 30 nt window. The tRNA-derived fragments (tRFs) have appeared as a major biotype among the vsRNAs, notably Ile-tRF and Ala-tRF, and were selectively loaded in OMVs. tRF-derived vsRNAs appear to be thermodynamically stable with at least 2 G-C basepairs and stem-loop structure. The analyzed tRF-derived vsRNAs are predicted to target several human host mRNAs with diverse functions. Bacterial vsRNAs and OMV-derived vsRNAs could be novel players likely modulating the intricate relationship between pathogens and their hosts.

<https://www.frontiersin.org/articles/10.3389/fmolb.2022.914991/full>

**Reviewer #2, comment 2:**

The aim of the present study is to demonstrate that Ile-tRF-5X which is transported by OMV modulates host response. The authors have clearly demonstrated the modulatory role of Ile-tRF-5X in the host using a transfection approach. However, when OMVs are used, the vehicle carrying Ile-tRF-5X, the results are sometimes different. This is understandable since OMVs contain many other molecules than Ile-tRF-5X such as other RNAs and also proteins... Therefore, it is not clear whether Ile-tRF-5X when present in OMV are able to modulate host response, the aim of the study. To demonstrate the regulatory role of Ile-tRF-5X contained in OMVs it would be necessary to compare WT OMVs with OMVs lacking Ile-tRF-5X. For that an E. coli mutant lacking Ile-tRF-5X or ILE-tRNA should be used. Such a mutant strain is probably not possible to obtain as discussed by the authors. Nevertheless, one can suppose that OMVs produced by a RNase E mutant, in which the level of ILE-tRNA is negatively affected could be used as OMVs with a decreased ILE-tRNA content. In addition, the authors could also use OMV-derived sRNA and OMVs treated with Ile-tRF-5X antisense, notably in experiments corresponding to figures 10/13/14 and S8.

**Response of the Authors:**

As discussed in the manuscript, we would have needed a mutant strain of the isoleucine tRNA and another of thIle-tRF-5X to better endorse the precise contribution at either the intracellular level or in OMVs. However, given the location of the genes, the removal of the three source sites would have led to adverse effects that would have biased data interpretation.

We believe that this is also the same problem with the use of OMVs from an E. coli culture where RNase E is deactivated. It is important to remember that RNase E is one of the three essential endoribonucleases in E. coli [49] and that its mutation can only be transient. There is a great risk of ending up with a mixed population of OMVs produced before and after the effect of transient inhibition. E. coli RNase E and its degradosome being at the interface of bacterial RNA processing and decay [49] can also generate byproducts that completely alter the profile of packaged RNAs in OMVs. In E. coli with inhibited RNase E, the intracellular RNA showed a general decrease in small fragments of 8-15nt and increase in larger fragments of 16-30nt (GEO accession: GSE200758, Bioproject accession: PRJNA826503). We did not analyze OMVs from E. coli RNase -.

We have unambiguously demonstrated the abundant presence of Ile-tRF-5X in intracellular RNA samples from E. coli and OMVs. We have shown clearly the specific contribution of Ile-tRF-5X in the modulation of host genes through interaction with MAP3K4 1) without ruling out the possibility of synergy with other vsRNAs, 2) without ruling out the possibility that these vsRNAs may be secreted in addition to delivery via OMVs. In the title we have highlighted the functionality of Ile-tRF-5X and the presence of this tRF in OMVs. We didn't define the functionality of Ile-tRF-5X by their presence in the OMVs.

Concerning the use of antisense in the pool of OMV-derived sRNAs (various population), the question is whether the observed effect on the level of MAP3K4 (increase or decrease) can definitely be attributed to the specific neutralization of Ile-tRNA ? In the unique pool consisting exclusively of Ile-tRNA in an equal ratio or with excess antisense it is easier to conclude.

**Minor comments**

**Reviewer #2, comment 3:**

Page 4: Only one example of trans-kingdom activity of regulatory sRNAs contained in OMVs is provided in the introduction section (Pseudomonas spp). Since the scientific literature is scarce in the field, the authors should provide more examples, notably introduce the recent work of Sahr and colleagues (DOI: 10.1038/s41467-022-28454-x) that studied the role of tRNA-Phe contained in OMVs.

**Response of the Authors:**

We acknowledge the remark and adjust the text of the introduction to include this work that we had read and recognize its significance. We had excluded this example because of the potential confusion about the nature of the functional tRNA in question: tRF vs. tRNA. Insofar as Sahr and colleagues highlighted a tRNA (Phe) and eliminated in their sequencing the reads inferior to 20nt (thus in theory our target of interest) we considered that there is a difference in the strategies although the objectives were similar.

**Reviewer #2, comment 4:**

Figure2A. Please indicate whether the Ile-tRF-5X level is significantly or not different in M63 Expo vs. Stat. In the discussion section (page 22), it is mentioned that the Ile-tRF-5X level remains unchanged during the Expo or Stat phase. Results presented in figure 2 seems to indicate otherwise.

**Response of the Authors:**

The statement was correct. We previously stipulated and defined the normal growth condition as the exponential phase at 37*C in LB culture and it is in this condition that we said there was no variation on the level of Ile-tRF-5X (Expo vs. Stat.). In the M63 medium, the difference between the exponential and stationary phase was significant (***p=0.0006). We have clarified it.

Statistics Figure 2A

**Reviewer #2, comment 5:**

- Evaluation of Ile-tRF-5X expression under different conditions (Figure2-3-4). Ile-tRF-5X expression was evaluated by LNA RT-qPCR. It is mentioned in the Figure 2 caption and M&M section that UniSp6 was used as spike-in control for cDNA synthesis and PCR amplification as well as reference gene for normalization. For gene expression normalization in RT-qPCR we used internal genes known to be (or supposed to be) constitutively expressed in the tested conditions. Spike-ins can be used to control RNA isolation and/or to monitor cDNA synthesis and/or amplification efficiency. I do not understand how this exogeneous spike-in “gene” can be used as an internal reference gene. Please clarify this crucial point.

**Response of the Authors:**

We refer to the answer to comment 4 of reviewer 1.

**Reviewer #1, comment 4:**

Since we did not know the exact mechanism of Ile-tRF-5X generation, we were unable to use the original tRNA (Ile-tRNA) as a control. However, we measured it under certain conditions as mentioned on page 8.

We mention on page 8: “A complementary RT-qPCR analysis (data not shown) showed that the level of tRNAs was affected by inhibition of RNA and protein synthesis, suggesting a lack of correlation between the level of tRNAs and that of tRFs, such as Ile-tRF-5X”. We discussed this issue on page 22.

In the experiments performed with HCT116 cells, ACTB and GAPDH were used as reference genes (internal standard) for gene monitoring such as MAP3K4. In the experiments performed only with bacteria, 23S and 16S were used as reference genes (tRNA-Ile be part of the 16S-23S rRNA ITS [19]).

In both situations UniSp6 served as a spike-in and thus as a normalizer as well. In table S2 (Document S2), we mentioned the sequences of these genes (ACTB, GAPDH, 23S, 16S) that were used for qPCR. When required, the Ile-tRF-5X is used in parallel with its negative control of the same length. Finally, the relative quantitation was calculated using the ∆∆Ct method (removing differences due to sampling and quality of the samples) [20].

We are aware of the importance of controls and standard setting, and we took the necessary precautions recommended by the literature [21]. The miRCURY LNA RT Kit used in this study include the RNA spike-in template for monitoring performance. Besides being a cDNA synthesis efficiency marker for small non coding RNA, Unisp6 is also used as endogenous control [22–24]. We believe that the use of spike-in included in the kits for cDNA synthesis is a reasonable option. Even the 16S and 23S in case of rifampicin treatment for example may be subjected to huge variations [25].

The other bacterial reference genes for gene expression studies by RT-qPCR have also not received the necessary validation or are equally subject to variations according to the treatment [26].There is no endogenous reference transcript which is known to be suitable for comparing the cellular levels of tRF/tRNA (or any other RNA species) during starvation to the levels during steady-state growth [27]. Sørensen et al., [27] propose to use a spike-in cells from a separate culture, which will be added to the experimental samples prior to RNA extraction. For the dual processing of our samples in RNA-Seq and RT-qPCR this method does not fit our primary objectives. We have clarified and detailed the information in the concerned sections to avoid any confusion.

**Extract from Table S2 (Document S2)**

| 23S rRNA | GTTAAGCGACTAAGCGTACAC  QIAGEN Universal reverse primer | EG30078 (EcoCyc) |
| --- | --- | --- |
| tRNA-Ile | CTTGTAGCTCAGGTGGTTAGAG  QIAGEN Universal reverse primer | EG30043 (EcoCyc) |
| 16S rRNA | TCTCTGAGATGTTCGCAAGC  QIAGEN Universal reverse primer | EG30089 (EcoCyc) |

**Reviewer #2, comment 6:**

P9, analysis of Ile-tRF-5X expression according to RNaseE/P availability: The quantification of Ile-tRF-5X expression in the RNaseE mutant measured at 44°C is compared to the level of Ile-tRF-5X at 37°C (reference condition). In this context, the reference condition should be 44°C??? Please comment. What is known about the modulation of RNAseE activity by environmental conditions such as LB vs. M63 and 37 vs 44°C? To make a link between the modulation of Ile-tRF-5x amount and RNaseE activity it would be interesting to determine Ile-tRF-5x expression in M63 medium in the WT and RNAse mutant strain.

**Response of the Authors:**

Here we used other strains, namely EM1277 (rne-3071 temperature sensitive) for RNase E and KP1036 (rnpA temperature sensitive) for RNase P. Therefore, the temperature is a leverage for the transient and temporally deactivation of the RNase E. The reference condition is the condition where RNase E is fully functional, i.e. at 30*C (not at 37*C, see Table S1 of document S2, see materials and methods). Most functional studies of RNase E and RNase P use these two types of strains or at least the heat-sensitive mutation approach to probe the contribution of the two endoribonucleases.

Indeed, the suggested experiment would bring a useful piece to the puzzle. RNase E is known to be involved in the glycolytic pathway of the bacteria [50] and hence we can speculate that its modulation may vary depending on the culture condition (LB vs. M63) more specifically on the parameter of carbon source availability in the M63 medium. The accumulation of tRF observed in M63 could therefore be, among other effects, the result of a decrease in RNase E efficiency due to its nutritional dependence.

We have integrated this point in the new version of the discussion. We will consider this in our further in situ functional studies of Ile-tRf-5X, as until now we have focused more on extracellular and inter-kingdom functionality.

**Reviewer #2, comment 7:**

Bacterial OMV and Ile-tRF-5X. It is mentioned that MG1665-derived OMVs contain Ile-tRF-5x. However, this information is not shown. It is shown that Ile-tRF-5X level is modulated by environmental conditions encountered by the bacterium. It would also have been interesting to determine whether the level of OMV-derived Ile-tRF-5X is also modulated by environmental conditions.

**Response of the Authors:**

Adjusted as recommended. We mentioned in the manuscript the references GEO (GSE200758) and Bioproject (PRJNA826503) where all the data of the study come from as well as the initial manuscript that describes the vsRNAs including in the OMVs. Delays in the publication process, beyond our control, have caused a gap. It was anticipated that at the time of this submission, the initial manuscript [37] would already be peer-reviewed and available online. Moreover, Figure S4 like Figure 6 shows the presence of Ile-tRF-5X in OMVs.

Indeed, we were curious about the levels of Ile-tRF-5X in OMVs as a function of the experimental conditions although we did not isolate OMVs from these conditions for this manuscript. De facto, the level of intracellular Ile-tRF-5X has an impact on the extracellular level (including OMVs) although the shortage of vsRNAs in OMVs remains poorly understood. We have some preliminary results that indicate variations in the level of Ile-tRf-5X. We hope to discuss and document this in a future study.

**Reviewer #2, comment 8:**

P12 “Mitogen-activated protein kinase 3, which carries no less than 10 potential binding sites”. Is the protein, the gene or the mRNA that contains the Ile-tRF-5X binding sites??? Please clarify the sentence.

**Response of the Authors:**

Adjusted as recommended. We had already mentioned in line 3 on page 12 that these are messenger targets.

**Reviewer #2, comment 9:**

Figure 7. It is mentioned that the size of the seed sequence ranked as 1 on MAP3K3 RNA (ie the Ile-tRF-5X binding site) is a 13-mer. Why only a 12-mer sequence is reported on the corresponding RNAhybrid box?

**Response of the Authors:**

Because this is how the RNAhybrid software presents it to us, but if we look at the 5'-end of MAP3K4 transcript just before the AGCTAAGCCT there is a "**G**" and similarly if we look at the 3’end of the Ile-tRF-5X before the UCGAUGUUCGA there is a **C**. So, it is indeed a perfect seed sequence of 13 nt. We did not want to modify the raw data of the software, hence the star mentioned in the box ***** and the explanation in the caption.

Ile-tRF-5X : 3’-**C**UCGAUGUUCGGA-5’

**Reviewer #2, comment 10:**

P14 “hinted to the presence of synergistic small RNAs”. At this stage, this part is very speculative and close to discussion. One can also suppose that the level of transfected Ile-tRF-5X is lower compared to the Ile-tRF-5X amount associated to OMV. Please modify the sentence.

**Response of the Authors:**

Corrected as suggested.

**Reviewer #2, comment 11:**

Figures 8 and 9. A variability of the impact of Ile-tRF-5X on MAP3K4 expression can be observed between experiments: 6-fold induction in figure 9A vs. 3-fold induction in figure 9C. It would have been interesting to compare the impact of OMV, OMVs-derived sRNAs and Ile-tRF-5X on MAP3K4 expression from the same experiments.

**Response of the Authors:**

Variability is observed but the significance is unambiguous. The ddCT is relative to the normalized control condition. Since OMVs and Ile-tRF-5X have different controls, combining them in a single experiment would not make a difference.

Figure 10B depicts the impact of OMVs vs. IletRF-5X on MAP3K4 from a single experiment.

**Reviewer #2, comment 12:**

P15 and figure 10: it is postulated that MAP3K4 expression is downregulated by miRNAs and that Ile-tRF-5X competes with miRNAs to upregulate MAP3K4. To demonstrate this hypothesis, the effect of Ile-tRF-5X on MAP3K4 expression was evaluated in a cell line (Dicer -/- ) in which miRNAs are mostly depleted and compared to the wild type cell line. MAP3K4 expression was evaluated both in WT and Dicer strains but from figure 10B we cannot directly compare the result of this comparative analysis since data were normalized to mock. Indeed, if the hypothesis is correct the expression of MAP3K4 should be higher in the Dicer strain even in absence of Ile-tRF-5X.

In addition, the authors showed that the positive impact of OMV and Ile-tRF-5X on MAP3K4 is reduced in the Dicer strain. This result does not support the initial hypothesis on a competition between miRNA and Ile-tRF-5X… Moreover, this result is contradictory with that presented in Figure 12C, which shows a loss of the inhibition of the compared Rluc reporter expression in the Dicer strain (100 % RLuc) when compared to the WT strain (40 % RLuc). Please clarify these apparent inconsistencies. Finally, I am wondering why the authors used OMV instead OMV-derived sRNAs in this experiment as OMVs and not OMV-derived sRNAs as a positive effect on the MAP3K4 protein level?

**Response of the Authors:**

We have mentioned these points in the discussion.

The (down/up)regulation of MAP3K4 may obviously not be under the exclusive control of miRNAs hence the use of luciferase assays to study the interactions in a more controlled environment. In figure 10 we deal the full “natural” MAP3K4 with n potential binding sites for Ile-tRF-5X while in the luciferase assays (Figure 12), we only observe (in an “on-board” system: psiCHECK2 plasmid) what is occurring in the 3'UTR.

As you pointed out, the data in Figure 10B are normalized not to the WT HCt116 vs. HCT116 Dicer -/- data but each data set has been normalized with the controls/mocks of the respective cell line. In both cases (with HCT116 WT or HCT116 Dicer -/-) there is an increase of MAP3K4 in the presence of OMVs or Ile-tRF-5X. OMVs seem to induce a stronger effect on MAP3K4. We modified the presentation to avoid any confusion. What the experiment also points out is the absence of a direct regulatory function of the Ile-tRF-5X (Ile-tRF-5X, in the Dicer -/- strain induces a slight upregulation of MAP3K4 but not significant). These observations led us to consider modulatory functions for vsRNAs

**Reviewer #2, comment 13:**

Figure 7 and Figure 11. Why are none of the Ile-tRF-5X binding sites shown in Figure 11 present in Figure 7?

**Response of the Authors:**

This is not the case.

Figure 7 shows the potential binding sites of Ile-tRF-5X that have been identified on the MAP3K4 transcript. For practical reasons, we have mentioned the 10 most important which are found on the ORF, on the 5'UTR and on the 3'UTR. While figure 11 is an illustrative figure that shows exclusively the Ile-tRF-5X binding sites found in the 3'UTR (as we are here in a context of comparison with miRNAs which targets are mainly located in the 3'UTR, we mention this fact in the manuscript).

In figure 7, the position numbers integrate each nucleotide of the transcript sequence since we are talking about the whole MAP3K4 whereas in figure 11 the position numbers start with the beginning of the 3'UTR of MAP3K4 since this is the section we are talking about. The N*2 (5453, 3'UTR), N*3 (5211, 3'UTR), N*8 (5104, 3'UTR), and N*10 (5235) in Figure 7 correspond to positions P426; P183; P77 and P208 in Figure 11, respectively. We have also specified that in figure 11 for obvious reasons of space we represent only the 5 binding sites of Ile-tRF-5X, which in theory largely exceed 5.

We believe that figures 7 and 11 are a valuable complimentary illustration in our manuscript and help in the comprehension of our strategy.

**Figure 7. Human MAP3K4 mRNA harbors potential binding sites for bacterial Ile-tRF-5X.**

Three bioinformatics tools (microRT, blastN and RNAhybrid) used in combination predict several Ile-tRF-5X (5X) binding sites in the human MAP3K4 mRNA. The base pairing of the top three Ile-tRF-5X binding sites is shown on the left. The number of nucleotides (mer) involved in the interaction (seed sequences), their minimal free energy (MFE, according to RNAhybrid) and their position (5’UTR, ORF or 3’UTR) are listed in the table. * Indicates that the 13th nt of Ile-tRF-5X is a C, thus allowing perfect base pairing with MAP3K4 mRNA in its ORF.

X

**Figure 11. Schematic representation of the predicted Ile-tRF-5X and miRNA base pairing in the MAP3K4 mRNA 3’UTR**. The base pairing interactions between bacterial Ile-tRF-5X and human MAP3K4 mRNA (RNAhybrid) are shown in red, with the minimum free energy (Mfe) and position (nucleotides). The base pairing interactions between human miRNAs and MAP3K4 mRNA (Targetscan) are highlighted in grey. The nucleotides involved in the Watson–Crick base pairing are in bold. The numbers in square brackets ([]) correspond to the number of non-displayed nucleotides in the 3’UTR of MAP3K4 mRNA. The full MAP3K4 mRNA 3′ UTR sequence was cloned downstream of the humanized Rluc (hRluc) gene, in the dual-luciferase reporter gene expression vector psiCHECK-II, with hFluc as a normalization control. hRluc, humanized *Renilla luciferase* gene; hluc+, humanized *Firefly luciferase* gene.

**Reviewer #2, comment 14:**

Figure8, 9, and 10. Please indicate in figure captions the quantity of OMV and Ile-tRF-5X used in the different experiments (as reported for figures 12 and 13).

**Response of the Authors:**

Corrected as requested.

**Reviewer #2, comment 15:**

A_ Figure 12AB and page 18: The author mentioned that the Rluc activity is less reduced when Ile-tRF-5X binding sites are mutated (35% of reduction) when compared to WT Ile-tRF-5X binding sites (60%). Are these differences really significant? If no, it seems difficult to suggest that mutations in Ile-tRF-5X binding sites affect the inhibitory elements of the 3’UTR as indicated by the authors.

B_ Page19 it is mentioned that “ Ile-tRF-5X was still able to restore Rluc activity, but at a reduced intensity…”. The Rluc activity in presence of Ile-tRF-5X (50 ng) appears similar with the WT (90%, Fig12A) and the mutated Ile-tRF-5X binding sites (90%, Fig12B) and not reduced when Ile-tRF-5X binding sites are mutated. Please correct.

C_ Moreover, how can we explain that Ile-tRF-5X (50 ng) still continues to activate the Rluc activity when Ile-tRF-5X binding sites are mutated (Fig12B)?

**Response of the Authors:**

**A**_ We should agree on the definition of significance. We judge it here only on the basis of statistics. We use almost systematically dual luciferase assays in all our experiments [8,9,51–54]. We have been careful to set up all the necessary controls to run the experiment and to perform the appropriate statistical tests to judge the significance of the data. One percent would have been significant if from a statistic standpoint this was the result we obtained. The addition of Ile-tRF-5X effectively (statistically) significantly restored the luciferase level.

**B**_ The sentence is correct. In Figure 12A we have an initial 40% luciferase activity and the addition of Ile-tRF-5X increased it to 90% (50nM) and then 130% (100nM). In figure 12B, the luciferase activity increases from 65% to 90%.

There is a recovery of the luciferase activity but with a reduced intensity :

WT= Amplitude of 50% vs. 25% between the initial activity and the addition of 50nM of Ile-tRF-5X.

Mut = Amplitude of 40% vs. 0% between the addition of 50nM vs. 100nM of Ile-tRF-5X

**C**_ Activated yes, but at a different percentage compared to the WT. The mutations concerned only 1/2 nucleotides of the sites (4.4% of total MAP3K4 3'UTR), so we do not annihilate all the interaction sites. This type of mutation allows us to probe the strength of interaction considering that the mutation of a few nucleotides with the impact on the level of luciferase activity is more significant than any other impact obtained by the total annihilation of the interaction site by the mutation.

**Reviewer #2, comment 16:**

A_ Figure 13. Why the cell proliferation level is 6-fold higher in absence of OMV (panel A, point 0) than in absence of Ile-tRF-5X (panel B, point 0)? This is the same condition? It is mentioned in the figure caption that data were normalized with its corresponding control. The controls are similar or different between the two panels? (Which are the control conditions?). Moreover, why two different statistical tests were applied between panel A (two-way ANOVA) and B (one-way ANOVA) with different post-hoc test, while their corresponding experimental design looks similar?

B_ I am not sure to really understand the experiment and the format used to present the data obtained is confusing. The XTT is used to quantify viable cells. Therefore, for example, 3.E3 cells are found viable after 48 h of incubation with the control, while 10.E3 cells are viable after 48 h of incubation in presence of 10 ng of OMV (Figure13 panelA). These raw data can be compared if the initial number of cells is strictly the same (this is rarely the case, since there is some variability from one well to another within a plate in cellular culture). To overcome this, the data are often expressed in a relative way by dividing the number of cells obtained by the number of initial cells and expressed as a percentage, eg 100% viable cells after 48h in the control conditions, vs. 200% after 48h in presence of OMV.
C_ Since it has been shown that OMV and sRNA OMV can have a different impact on the MAPK pathways (Figure 8 vs Figure 9), an antisense Ile-tRF-5X has no impact on OMV-induced cell proliferation (Fig S7), and OMV can induce cell mortality (but not Ile-tRF-5X), It would be interesting and necessary to see and compare the effect of OMV-derived sRNA on cell proliferation.

D_ Finally, why the authors decided to measure cell proliferation after 48h of interaction (incubation). This time is surprising as they showed a drastic drop (74%) of Ile-tRF-5X level in HCT116 cells at 48 h when compared to its level at 24h (figure 6)?

**Response of the Authors:**

**A**_ We have explicitly mentioned that HCT116 cells were incubated with increasing amount of bacterial OMVs (panel A) or transfected with increasing amount of Ile-tRF-5X (panel B). So, it’s two independent and different experiences. The controls can indeed not be identical, and we detail it throughout the manuscript in the sense that all experiments with Ile-tRF-5X are performed at the same time as the transfection of a negative control of the same length and for OMVs, PBS and LPS are used as controls.

If the hypothesis is the following: Ile-tRF-5X, on the one hand, and OMVs, on the other hand, have a significant effect on the proliferation of HCT116, we would like to specify that our data remain significant whatever the statistical test used. We did not choose the test that suits us for significance purposes. All statistical tests were performed with GraphPad which suggests the most appropriate correction test. After normalization with the respective controls, in panel A, we had not only OMVs as parameter/group (but also OMVs + As-5X) while in panel B ile-tRF-5X was the only parameter/group. In this case GraphPad suggests the two-way and the one-way respectively. Nevertheless, to avoid any confusion and to answer the request, we resubmitted the data of panel A and B to a unique statistical test.

**B**_ No, starting from the same amount of initial HCT116 cells at T=0, the data set presented in panels A and B represent what was measured/observed after 48h of incubation with OMVs or after 48h of transfection with Ile-tRF-5X. All followed by a 3h XTT treatment.

Data displayed in panels A and B were first normalized with their respective controls before relative deduction (based on the absorbance) of cell number. In percentage or in number of cells, we found that there was less growth in the absence of OMVs or Ile-tRF-5X.

**C**_ In this study, for reasons beyond our control, we focused on the role and function of Ile-tRF-5X. We have the complete repertoire of OMV-derived sRNAs (by sequencing) and expect to investigate their respective functions according to their biotypes.

**D**_ We have conducted preliminary experiments showing no impact at 24h. The levels of Ile-tRF-5X are certainly more important at 24h but the amplitude of the modulations that it can induce could take more time to be observed. There is certainly a decrease in the level of Ile-tRF-5X at 48h but the question is to ascertain whether it is a degradation because we can also presume integration/sequestration in protein complexes making it inaccessible.

**Reviewer #2, comment 17:**

Figure 14. The ability to induce the expression of various HCT116 genes differs between OMVs and Ile-tRF-5X. To claim that “bacterial OMVs and their Ile-tRF-5X content are
involved in the controlled regulation of human cell proliferation and death”, It should also have been determined the effect of OMV-derived sRNA OMVs on gene expression, notably Casp-1, Bax and Bcl-2. In addition, Ile-tRF-5X induces MAP3K4 expression without inducing the upregulation of MAP3K4 at the protein level. In this context, how to explain the impact of Ile-tRF-5X on P38a expression?

**Response of the Authors:**

We rephrased the sentence.

The quantification performed here was done at the messenger level and as such, the accumulation of MAp3K4 can effectively trigger a cascade of stimulation of messengers located in the same pathway. Here, we have highlighted the link between MAP3K4 and Ile-tRF-5X, but it is also conceivable that Ile-tRf-5X may also directly target P38a.

Throughout the manuscript, we attempted to highlight the global effect of OMVs and the isolated effect of Ile-tRF-5X with the ultimate goal of reconsidering the biological function of vsRNAs (<16 nt) and OMVs as the optimal vector in a host-pathogen context.

**Reviewer #2, comment 18:**

Page 22: “However, under normal growth conditions, either during…”. Can we consider that a growth in LB medium corresponds to a normal growth condition for an intestinal bacterium? I think the most appropriate term is “normal laboratory conditions.”

**Response of the Authors:**

Corrected as requested.

However, we would like to point out that:

LB is indeed used to mimic the growth in normal condition of an intestinal bacterium [55] and that the strain used in this study (K-12 MG1655) is a non-pathogenic laboratory strain with minimal genetic manipulation and therefore considered to be one of the most similar to wild E. coli [56].

**Reviewer #2, comment 19:**

A_ Page 24: “The selective enrichment of vsRNAs in OMVs and their effective transfer to human host cells were previously evidenced by other teams (34,40,81,82).” In the present MS, the authors never show an enrichment of RNA into OMV. This part is not appropriate.

B_ In addition, the comparison of the effect of OMV in conventionalized vs. germ-free mice is out of the scope of the MS.

**Response of the Authors:**

**A_** We refer to the answer given in comment 7 (**Reviewer #2, comment 7:** )

We mentioned in the manuscript the references GEO (GSE200758) and Bioproject (PRJNA826503) where all the data of the study come from as well as the initial manuscript that describes the vsRNAs including in the OMVs. Delays in the publication process, beyond our control, have caused a gap. It was anticipated that at the time of this submission, the initial manuscript [37] would already be peer-reviewed and available online. Moreover, Figure S4 and Figure 6 shows the presence of Ile-tRF-5X in OMVs.

**B_** We have only suggested the comparison of the effect of OMV in conventionalized vs. germ-free mice. We did not formulate any argument about it. However, we agree to remove the sentence.

**Reviewer #2, comment 20:**

Page 25: I am not sure that the potential adjuvant effect of OMV can be an argument to explain why OMV can induce MAP3K4 at the protein level protein and not it sRNA content…

**Response of the Authors:**

We have removed the sentence. Before reaching this argument, we described other properties that could explain the result.

**Reviewer #2, comment 21:**

Page 27: “which is required for cell proliferation and survival…”. It means that Ile-tRF-5X displays proto-oncogene activity. I think this part of the discussion (including the end) should be weighted. Indeed, the data generated were obtained from a colon cancer cell line.

**Response of the Authors:**

We have reworded the sentence because here we were talking about p38 which is indeed shown to be involved in cell proliferation in colorectal cancer cells [57].

We strongly believe in these perspectives and wish to keep them as long as we do not mislead our readers and we do not over-interpret our results. We deemed to have weighed the text through these passages:

“Given the discrepancy observed in the induction of Bax (pro-death) and Bcl-2 (pro-survival) transcripts by OMVs, on one hand, and the vsRNA Ile-tRF-5X on the other hand, we can speculate on an in-balance bacterial modulation between cell proliferation and host cell death program”

“It is necessary, however, to see at the protein level if all these patterns and phenotypes are confirmed and more precisely how they are affected in an in vivo experiment.”

“Thus, fluctuation in proliferation may be related to mitochondrial abundance and cell viability (vs. death).”

Furthermore, we have used 35 times "may", 10 times "seem" and 5 times "could" as a precaution in the manuscript even with statistically significant data. We remain open to your suggestions on which parts of the text should be removed or reworded.

**We wish to thank Reviewer #2 for his/her assessment of our manuscript and hope that the revised version of our manuscript is now suitable for publication in *PLOS PATHOGENS.***

**References**

1.	Ghosal, A.; Upadhyaya, B.B.; Fritz, J.V.; Heintz-Buschart, A.; Desai, M.S.; Yusuf, D.; Huang, D.; Baumuratov, A.; Wang, K.; Galas, D.; et al. The Extracellular RNA Complement of Escherichia Coli. *Microbiologyopen* **2015**, *4*, 252–266, doi:10.1002/mbo3.235.

2.	Koeppen, K.; Hampton, T.H.; Jarek, M.; Scharfe, M.; Gerber, S.A.; Mielcarz, D.W.; Demers, E.G.; Dolben, E.L.; Hammond, J.H.; Hogan, D.A.; et al. A Novel Mechanism of Host-Pathogen Interaction through SRNA in Bacterial Outer Membrane Vesicles. *PLOS Pathogens* **2016**, *12*, e1005672, doi:10.1371/journal.ppat.1005672.

3.	Choi, J.-W.; Kim, S.-C.; Hong, S.-H.; Lee, H.-J. Secretable Small RNAs via Outer Membrane Vesicles in Periodontal Pathogens. *J Dent Res* **2017**, *96*, 458–466, doi:10.1177/0022034516685071.

4.	Blenkiron, C.; Simonov, D.; Muthukaruppan, A.; Tsai, P.; Dauros, P.; Green, S.; Hong, J.; Print, C.G.; Swift, S.; Phillips, A.R. Uropathogenic Escherichia Coli Releases Extracellular Vesicles That Are Associated with RNA. *PLOS ONE* **2016**, *11*, e0160440, doi:10.1371/journal.pone.0160440.

5.	Mu, W.; Jia, Y.; Chen, X.; Li, H.; Wang, Z.; Cheng, B. Intracellular Porphyromonas Gingivalis Promotes the Proliferation of Colorectal Cancer Cells via the MAPK/ERK Signaling Pathway. *Front Cell Infect Microbiol* **2020**, *10*, 584798, doi:10.3389/fcimb.2020.584798.

6.	Zhang, H.; Zhang, Y.; Song, Z.; Li, R.; Ruan, H.; Liu, Q.; Huang, X. SncRNAs Packaged by Helicobacter Pylori Outer Membrane Vesicles Attenuate IL-8 Secretion in Human Cells. *International Journal of Medical Microbiology* **2020**, *310*, 151356, doi:10.1016/j.ijmm.2019.151356.

7.	Garcia-Silva, M.R.; Cabrera-Cabrera, F.; Cura das Neves, R.F.; Souto-Padrón, T.; de Souza, W.; Cayota, A. Gene Expression Changes Induced by Trypanosoma Cruzi Shed Microvesicles in Mammalian Host Cells: Relevance of TRNA-Derived Halves. *BioMed Research International* **2014**, *2014*, e305239, doi:10.1155/2014/305239.

8.	Plante, I.; Plé, H.; Landry, P.; Gunaratne, P.H.; Provost, P. Modulation of MicroRNA Activity by Semi-MicroRNAs. *Front Genet* **2012**, *3*, 99, doi:10.3389/fgene.2012.00099.

9.	Lambert, M.; Benmoussa, A.; Diallo, I.; Ouellet-Boutin, K.; Dorval, V.; Majeau, N.; Joly-Beauparlant, C.; Droit, A.; Bergeron, A.; Têtu, B.; et al. Identification of Abundant and Functional DodecaRNAs (DoRNAs) Derived from Ribosomal RNA. *International Journal of Molecular Sciences* **2021**, *22*, 9757, doi:10.3390/ijms22189757.

10.	Lambert, M.; Guellal, S.; Ho, J.; Benmoussa, A.; Laffont, B.; Bélanger, R.; Provost, P. An Expanded Landscape of Unusually Short RNAs in 11 Samples from Six Eukaryotic Organisms. *Non-Coding RNA* **2022**, *8*, 34, doi:10.3390/ncrna8030034.

11.	Söderbom, F.; Svärd, S.G.; Kirsebom, L.A. RNase E Cleavage in the 5’ Leader of a TRNA Precursor. *J Mol Biol* **2005**, *352*, 22–27, doi:10.1016/j.jmb.2005.07.003.

12.	Ow, M.C.; Kushner, S.R. Initiation of TRNA Maturation by RNase E Is Essential for Cell Viability in E. Coli. *Genes Dev* **2002**, *16*, 1102–1115, doi:10.1101/gad.983502.

13.	Li, Z.; Deutscher, M.P. RNase E Plays an Essential Role in the Maturation of Escherichia Coli TRNA Precursors. *RNA* **2002**, *8*, 97–109.

14.	Palazzo, A.F.; Lee, E.S. Non-Coding RNA: What Is Functional and What Is Junk? *Frontiers in Genetics* **2015**, *6*.

15.	Kennell, D. Titration of the Gene Sites on DNA by DNA-RNA Hybridization: II. The Escherichia Coli Chromosome. *Journal of Molecular Biology* **1968**, *34*, 85–103, doi:10.1016/0022-2836(68)90236-2.

16.	Baracchini, E.; Bremer, H. Determination of Synthesis Rate and Lifetime of Bacterial MRNAs. *Analytical Biochemistry* **1987**, *167*, 245–260, doi:10.1016/0003-2697(87)90160-6.

17.	Thankaswamy-Kosalai, S.; Sen, P.; Nookaew, I. Evaluation and Assessment of Read-Mapping by Multiple next-Generation Sequencing Aligners Based on Genome-Wide Characteristics. *Genomics* **2017**, *109*, 186–191, doi:10.1016/j.ygeno.2017.03.001.

18.	Li, H.; Homer, N. A Survey of Sequence Alignment Algorithms for Next-Generation Sequencing. *Brief Bioinform* **2010**, *11*, 473–483, doi:10.1093/bib/bbq015.

19.	Sekiya, T.; Nishimura, S. Sequence of the Gene for Isoleucine TRNA1 and the Surrounding Region in a Ribosomal RNA Operon of Escherichia Coli. *Nucleic Acids Res* **1979**, *6*, 575–592.

20.	Livak, K.J.; Schmittgen, T.D. Analysis of Relative Gene Expression Data Using Real-Time Quantitative PCR and the 2(-Delta Delta C(T)) Method. *Methods* **2001**, *25*, 402–408, doi:10.1006/meth.2001.1262.

21.	Bustin, S.A.; Benes, V.; Garson, J.A.; Hellemans, J.; Huggett, J.; Kubista, M.; Mueller, R.; Nolan, T.; Pfaffl, M.W.; Shipley, G.L.; et al. The MIQE Guidelines: Minimum Information for Publication of Quantitative Real-Time PCR Experiments. *Clin. Chem.* **2009**, *55*, 611–622, doi:10.1373/clinchem.2008.112797.

22.	Lak, R.; Yaghobi, R.; Garshasbi, M. Importance of MiR-125a-5p and MiR-122-5p Expression in Patients with HBV Infection. *Cell Mol Biol (Noisy-le-grand)* **2020**, *66*, 1–8.

23.	Lunavat, T.R.; Cheng, L.; Kim, D.-K.; Bhadury, J.; Jang, S.C.; Lässer, C.; Sharples, R.A.; López, M.D.; Nilsson, J.; Gho, Y.S.; et al. Small RNA Deep Sequencing Discriminates Subsets of Extracellular Vesicles Released by Melanoma Cells – Evidence of Unique MicroRNA Cargos. *RNA Biol* **2015**, *12*, 810–823, doi:10.1080/15476286.2015.1056975.

24.	Ganji, S.M.; Saidijam, M.; Amini, R.; Mousavi-Bahar, S.H.; Shabab, N.; Seyedabadi, S.; Mahdavinezhad, A. Evaluation of MicroRNA-99a and MicroRNA-205 Expression Levels in Bladder Cancer. *Int J Mol Cell Med* **2017**, *6*, 87–95, doi:10.22088/acadpub.BUMS.6.2.3.

25.	Hamouche, L.; Poljak, L.; Carpousis, A.J. Ribosomal RNA Degradation Induced by the Bacterial RNA Polymerase Inhibitor Rifampicin. *RNA* **2021**, *27*, 946–958, doi:10.1261/rna.078776.121.

26.	Rocha, D.J.P.; Santos, C.S.; Pacheco, L.G.C. Bacterial Reference Genes for Gene Expression Studies by RT-QPCR: Survey and Analysis. *Antonie van Leeuwenhoek* **2015**, *108*, 685–693, doi:10.1007/s10482-015-0524-1.

27.	Sørensen, M.A.; Fehler, A.O.; Lo Svenningsen, S. Transfer RNA Instability as a Stress Response in Escherichia Coli: Rapid Dynamics of the TRNA Pool as a Function of Demand. *RNA Biol* **2018**, *15*, 586–593, doi:10.1080/15476286.2017.1391440.

28.	Mohanty, B.K.; Kushner, S.R. Ribonuclease P Processes Polycistronic TRNA Transcripts in Escherichia Coli Independent of Ribonuclease E. *Nucleic Acids Res* **2007**, *35*, 7614–7625, doi:10.1093/nar/gkm917.

29.	Kelly, K.O.; Deutscher, M.P. The Presence of Only One of Five Exoribonucleases Is Sufficient to Support the Growth of Escherichia Coli. *Journal of Bacteriology* **1992**, *174*, 6682–6684, doi:10.1128/jb.174.20.6682-6684.1992.

30.	Chen, P.; Jäger, G.; Zheng, B. Transfer RNA Modifications and Genes for Modifying Enzymes in Arabidopsis Thaliana. *BMC Plant Biology* **2010**, *10*, 201, doi:10.1186/1471-2229-10-201.

31.	Chan, C.M.; Huang, R.H. Enzymatic Characterization and Mutational Studies of TruD – the Fifth Family of Pseudouridine Synthases. *Archives of Biochemistry and Biophysics* **2009**, *489*, 15–19, doi:10.1016/j.abb.2009.07.023.

32.	Yarian, C.S.; Basti, M.M.; Cain, R.J.; Ansari, G.; Guenther, R.H.; Sochacka, E.; Czerwinska, G.; Malkiewicz, A.; Agris, P.F. Structural and Functional Roles of the N1- and N3-Protons of Psi at TRNA’s Position 39. *Nucleic Acids Res* **1999**, *27*, 3543–3549, doi:10.1093/nar/27.17.3543.

33.	Mehta, R.; Champney, W.S. Neomycin and Paromomycin Inhibit 30S Ribosomal Subunit Assembly in Staphylococcus Aureus. *Curr Microbiol* **2003**, *47*, 0237–0243, doi:10.1007/s00284-002-3945-9.

34.	Lee, Y.S.; Shibata, Y.; Malhotra, A.; Dutta, A. A Novel Class of Small RNAs: TRNA-Derived RNA Fragments (TRFs). *Genes Dev.* **2009**, *23*, 2639–2649, doi:10.1101/gad.1837609.

35.	Kumar, P.; Anaya, J.; Mudunuri, S.B.; Dutta, A. Meta-Analysis of TRNA Derived RNA Fragments Reveals That They Are Evolutionarily Conserved and Associate with AGO Proteins to Recognize Specific RNA Targets. *BMC Biology* **2014**, *12*, 78, doi:10.1186/s12915-014-0078-0.

36.	Kumar, P.; Anaya, J.; Mudunuri, S.B.; Dutta, A. Meta-Analysis of TRNA Derived RNA Fragments Reveals That They Are Evolutionarily Conserved and Associate with AGO Proteins to Recognize Specific RNA Targets. *BMC Biology* **2014**, *12*, 78, doi:10.1186/s12915-014-0078-0.

37.	Diallo, I.; Ho, J.; Lalaouna, D.; Massé, E.; Provost, P. RNA Sequencing Unveils Very Small RNAs With Potential Regulatory Functions in Bacteria. *Frontiers in Molecular Biosciences* **2022**, *9*.

38.	Shepherd, J.; Ibba, M. Bacterial Transfer RNAs. *FEMS Microbiol Rev* **2015**, *39*, 280–300, doi:10.1093/femsre/fuv004.

39.	Tsatsaronis, J.A.; Franch-Arroyo, S.; Resch, U.; Charpentier, E. Extracellular Vesicle RNA: A Universal Mediator of Microbial Communication? *Trends Microbiol* **2018**, *26*, 401–410, doi:10.1016/j.tim.2018.02.009.

40.	Sender, R.; Fuchs, S.; Milo, R. Revised Estimates for the Number of Human and Bacteria Cells in the Body. *PLOS Biology* **2016**, *14*, e1002533, doi:10.1371/journal.pbio.1002533.

41.	Hayashi, K.; Morooka, N.; Yamamoto, Y.; Fujita, K.; Isono, K.; Choi, S.; Ohtsubo, E.; Baba, T.; Wanner, B.L.; Mori, H.; et al. Highly Accurate Genome Sequences of Escherichia Coli K-12 Strains MG1655 and W3110. *Mol Syst Biol* **2006**, *2*, 2006.0007, doi:10.1038/msb4100049.

42.	Barrila, J.; Crabbé, A.; Yang, J.; Franco, K.; Nydam, S.D.; Forsyth, R.J.; Davis, R.R.; Gangaraju, S.; Ott, C.M.; Coyne, C.B.; et al. Modeling Host-Pathogen Interactions in the Context of the Microenvironment: Three-Dimensional Cell Culture Comes of Age. *Infection and Immunity* *86*, e00282-18, doi:10.1128/IAI.00282-18.

43.	Kim, Y.-K.; Kim, B.; Kim, V.N. Re-Evaluation of the Roles of DROSHA, Exportin 5, and DICER in MicroRNA Biogenesis. *Proc Natl Acad Sci U S A* **2016**, *113*, E1881–E1889, doi:10.1073/pnas.1602532113.

44.	Yang, J.-S.; Maurin, T.; Robine, N.; Rasmussen, K.D.; Jeffrey, K.L.; Chandwani, R.; Papapetrou, E.P.; Sadelain, M.; O’Carroll, D.; Lai, E.C. Conserved Vertebrate Mir-451 Provides a Platform for Dicer-Independent, Ago2-Mediated MicroRNA Biogenesis. *Proc Natl Acad Sci U S A* **2010**, *107*, 15163–15168, doi:10.1073/pnas.1006432107.

45.	Saetrom, P.; Heale, B.S.E.; Snøve, O.; Aagaard, L.; Alluin, J.; Rossi, J.J. Distance Constraints between MicroRNA Target Sites Dictate Efficacy and Cooperativity. *Nucleic Acids Res* **2007**, *35*, 2333–2342, doi:10.1093/nar/gkm133.

46.	Kendziorski, C.M.; Zhang, Y.; Lan, H.; Attie, A.D. The Efficiency of Pooling MRNA in Microarray Experiments. *Biostatistics* **2003**, *4*, 465–477, doi:10.1093/biostatistics/4.3.465.

47.	Kendziorski, C.; Irizarry, R.A.; Chen, K.-S.; Haag, J.D.; Gould, M.N. On the Utility of Pooling Biological Samples in Microarray Experiments. *Proc Natl Acad Sci U S A* **2005**, *102*, 4252–4257, doi:10.1073/pnas.0500607102.

48.	GLASS, Ä.; HENNING, J.; KAROPKA, T.; SCHEEL, T.; BANSEMER, S.; KOCZAN, D.; GIERL, L.; ROLFS, A.; GIMSA, U. Representation of Individual Gene Expression in Completely Pooled MRNA Samples. *Bioscience, Biotechnology, and Biochemistry* **2005**, *69*, 1098–1103, doi:10.1271/bbb.69.1098.

49.	Mackie, G.A. RNase E: At the Interface of Bacterial RNA Processing and Decay. *Nat. Rev. Microbiol.* **2013**, *11*, 45–57, doi:10.1038/nrmicro2930.

50.	Tamura, M.; Moore, C.J.; Cohen, S.N. Nutrient Dependence of RNase E Essentiality in Escherichia Coli. *J Bacteriol* **2013**, *195*, 1133–1141, doi:10.1128/JB.01558-12.

51.	Lambert, M.; Pépin, G.; Peralta-Zaragoza, O.; Matusiak, R.; Ly, S.; Landry, P.; Provost, P. TWEAK Negatively Regulates Human Dicer. *Noncoding RNA* **2016**, *2*, 12, doi:10.3390/ncrna2040012.

52.	Diallo, I.; Husseini, Z.; Guellal, S.; Vion, E.; Ho, J.; Kozak, R.A.; Kobinger, G.P.; Provost, P. Ebola Virus Encodes Two MicroRNAs in Huh7-Infected Cells. *International Journal of Molecular Sciences* **2022**, *23*, 5228, doi:10.3390/ijms23095228.

53.	Boissonneault, V.; St-Gelais, N.; Plante, I.; Provost, P. A PCR-Based Cloning Strategy Applicable to Functional MicroRNA Studies. *Anal Biochem* **2008**, *381*, 166–168, doi:10.1016/j.ab.2008.06.026.

54.	Perron, M.P.; Provost, P. Protein Interactions and Complexes in Human MicroRNA Biogenesis and Function. *Front Biosci* **2008**, *13*, 2537–2547.

55.	Wan, C.; Wu, K.; Lu, X.; Fang, F.; Li, Y.; Zhao, Y.; Li, S.; Gao, J. Integrative Analysis of the Gut Microbiota and Metabolome for In Vitro Human Gut Fermentation Modeling. *J Agric Food Chem* **2021**, *69*, 15414–15424, doi:10.1021/acs.jafc.1c04259.

56.	Blattner, F.R.; Plunkett, G.; Bloch, C.A.; Perna, N.T.; Burland, V.; Riley, M.; Collado-Vides, J.; Glasner, J.D.; Rode, C.K.; Mayhew, G.F.; et al. The Complete Genome Sequence of Escherichia Coli K-12. *Science* **1997**, *277*, 1453–1462, doi:10.1126/science.277.5331.1453.

57.	Comes, F.; Matrone, A.; Lastella, P.; Nico, B.; Susca, F.C.; Bagnulo, R.; Ingravallo, G.; Modica, S.; Lo Sasso, G.; Moschetta, A.; et al. A Novel Cell Type-Specific Role of P38 α in the Control of Autophagy and Cell Death in Colorectal Cancer Cells. *Cell Death & Differentiation* **2007**, *14*, 693–702, doi:10.1038/sj.cdd.4402076.
