# Supplementary material for: A tRNA-derived fragment present in E. coli OMVs regulates host cell gene expression and proliferation
Source: PLoS Pathog. 2022 Sep 15;18(9):e1010827. doi: 10.1371/journal.ppat.1010827 (PMC9514646; doi:10.1371/journal.ppat.1010827)
Supplement: Attachment — Submitted filename: 220802_Point-by-point response_2nd round_minor.docx [file ppat.1010827.s017.docx]

**Our responses :** **in red**

Dear Dr. Provost,

Thank you very much for submitting your manuscript "A tRNA-derived fragment present in E. coli OMVs regulates host cell gene expression and proliferation" for consideration at PLOS Pathogens. As with all papers reviewed by the journal, your manuscript was reviewed by members of the editorial board and by several independent reviewers. The reviewers appreciated the attention to an important topic. Based on the reviews, we are likely to accept this manuscript for publication, providing that you modify the manuscript according to the review recommendations.

Please carefully read over the manuscript and modify the text as requested by the referees. Then, prepare and submit your revised manuscript within 30 days. If you anticipate any delay, please let us know the expected resubmission date by replying to this email.

When you are ready to resubmit, please upload the following:

[1] A letter containing a detailed list of your responses to all review comments, and a description of the changes you have made in the manuscript.

Please note while forming your response, if your article is accepted, you may have the opportunity to make the peer review history publicly available. The record will include editor decision letters (with reviews) and your responses to reviewer comments. If eligible, we will contact you to opt in or out

[2] Two versions of the revised manuscript: one with either highlights or tracked changes denoting where the text has been changed; the other a clean version (uploaded as the manuscript file).

Important additional instructions are given below your reviewer comments.

Thank you again for your submission to our journal. We hope that our editorial process has been constructive so far, and we welcome your feedback at any time. Please don't hesitate to contact us if you have any questions or comments.

Sincerely,

Mathieu Coureuil

Guest Editor

PLOS Pathogens

Xavier Nassif

Section Editor

PLOS Pathogens

Kasturi Haldar

Editor-in-Chief

PLOS Pathogens

orcid.org/0000-0001-5065-158X

Michael Malim

Editor-in-Chief

PLOS Pathogens

orcid.org/0000-0002-7699-2064

**Reviewer Comments (if any, and for reference):**

Reviewer's Responses to Questions

**Part I - Summary**
Please use this section to discuss strengths/weaknesses of study, novelty/significance, general execution and scholarship.

Reviewer #1: The authors responded to the major comments and modified the manuscript accordingly.

Reviewer #2: The authors have responded point by point to all my previous remarks and modified the text accordingly. I strongly recommend the publication of the present MS in the PLOS pathogens journal.

**Response:** Thank you for agreeing to review our manuscript.

**Part II – Major Issues: Key Experiments Required for Acceptance**
Please use this section to detail the key new experiments or modifications of existing experiments that should be absolutely required to validate study conclusions.

Reviewer #1: The major concern was the availability of the previous data cited in the manuscript. The paper has been now published in Frontiers in Mol Biosciences and the authors included this citation throughout the manuscript.

Reviewer #2: No new experiments are required

**Part III – Minor Issues: Editorial and Data Presentation Modifications**
Please use this section for editorial suggestions as well as relatively minor modifications of existing data that would enhance clarity.

Reviewer #1:

The minor concerns have been mainly addressed. Few points like the names of the bacterial species in italic in the text need to be checked in final version. Some remaining typos should be corrected like Fig 4 legend "Bacteria RNase E" to be changed to "Bacterial RNase E" etc.

**Response:** corrected as requested

Reviewer #2:

Page 2: abstract section : Check the bracket in italic after E. coli : (E. coli)

**Response:** corrected as requested
Page 4: Please check “Bacterial Extracellular vesicles”

**Response:** corrected as requested

Page 4: “L. pneumophila”. For the first time, please mention the species with its full name and in italic.

**Response:** corrected as requested

Page 5: “Pseudomonas… HG001”, please check the typo.

**Response:** corrected as requested

Page5: “These vsRNAs were highly abundant in bacteria and their derived OMVs”. It seems to me that this has only been demonstrated for E. coli derived OMV… Please modify the sentence.
Figure 1 caption: Please modify “isoleucin” by isoleucine and indicate genes in italic and without upper case for the first letter.

**Response:** corrected as requested

Page 7 (lane2): E. coli in italic.

**Response:** corrected as requested

Page 13: Please change Figure S54 by Figure S5. In addition, in Figure S5 caption two panels are mentioned (A for 2h and B for 18h). However, only one panel is shown in Fig. S5. Please modify.

**Response:** corrected as requested

Figure 6 legend: check “withE. coli”. Please also indicate the quantity of OMV and synthetic Ile-trf-5X used like in Figure 7 and 8

**Response:** corrected as requested

Figure 7 legend: change “10ng” by “10 ng”

**Response:** corrected as requested

Page 17: I didn’t find in the MS any information about the mutated Ile-tRF-5X (sequence, ..)

**Response:** corrected as requested : see Document S2, table S2

Figure 9 legend: please modify “10ng” and “100nM”

**Response:** corrected as requested

Page 25: Check the double bracket after reference 60

**Response:** corrected as requested

Page 25: please modify “may seems counterintuitive”

**Response:** corrected as requested

PLOS authors have the option to publish the peer review history of their article ([what does this mean?](https://journals.plos.org/plospathogens/s/editorial-and-peer-review-process#loc-peer-review-history)). If published, this will include your full peer review and any attached files.


If you choose “no”, your identity will remain anonymous but your review may still be made public.


**Do you want your identity to be public for this peer review?** For information about this choice, including consent withdrawal, please see our [Privacy Policy](https://www.plos.org/privacy-policy).

Reviewer #1: No

Reviewer #2: **Yes:** Éric GUÉDON
